# Fishing for millennia: Effects and impacts of prehistoric fishing in the Syltholm Fjord, Denmark

**DOI:** 10.1371/journal.pone.0347863

**Published:** 2026-05-13

**Authors:** Daniel Groß, Sofie Folsach Hellerøe, Satu Koivisto, Ulrich Schmölcke

**Affiliations:** 1 Museum Lolland-Falster, Nykøbing F., Denmark; 2 Department of Archaeology and Heritage Studies, Aarhus University, Højbjerg, Denmark; 3 Leibniz-Zentrum für Archäologie, Schloss Gottorf, Schleswig, Germany; Israel Antiquities Authority, ISRAEL

## Abstract

This study investigates the long-term impacts of human subsistence strategies on the fauna and ecosystem of Syltholm Fjord, Denmark, from the Late Mesolithic to the Bronze Age (c. 4500–800 cal BCE). Drawing on data from 17 archaeological excavations, we examine how long-term stationary wooden fishing structures in a lagoon-like environment and terrestrial resource exploitation influenced species composition, biodiversity, and human subsistence strategies at coastal settlements on a relatively small island (c. 1200 km^2^). Faunal analyses reveal that while dominant fish species remained consistent across periods, terrestrial fauna exhibited shifts, particularly around 3000 cal BCE, with an increased reliance on wild game coinciding with a decline in the use of fish weirs. Diversity indices indicate a significant reduction in species richness from the Early Neolithic to the Bronze Age, suggesting a more homogeneous ecosystem potentially reflecting intensified anthropogenic influence and perhaps increased social complexity. Salinity and sediment reconstructions, together with prey choice models (PCM), highlight the persistence of aquatic resources in the diet and suggest that human foraging strategies continued to optimize energetic returns without substantially altering fishing practices. Our findings challenge the notion of an abrupt Neolithic dietary transition toward domesticates’ dominance, illustrating instead a mosaic subsistence pattern that integrates wild aquatic and terrestrial resources over millennia. The diachronic stability of the faunal composition, coupled with reduced but persistent biodiversity, implies a long-term anthropogenic shaping of the landscape, possibly linked to communal management and later hierarchical structures. This case study underscores the importance of integrating archaeological, ecological, and theoretical perspectives to understand local trajectories of human-environment interaction and social change in prehistoric southern Scandinavia.

## Introduction

Human contribution to climate and ecosystems has affected natural environments to such a degree that is irreversible. The presence of human traces in geological archives have therefore led to the introduction of the term Anthropocene, albeit a debated one, for the recent couple of millennia [e.g., 1–3].

While the global consequences of human impact on the environment are critical for planetary development, also superregional and regional anthropogenic impacts do play a relevant role. Coastal regions have always been more strongly influenced by local human activities than other areas of the planet – with corresponding ecological and ultimately societal consequences [2]. In this contribution we therefore discuss the effects of several millennia of fishing with stationary wooden structures in the prehistoric Syltholm Fjord, Denmark [4] with a focus on its impacts on the local fauna and subsistence strategies.

**Fig 1:**
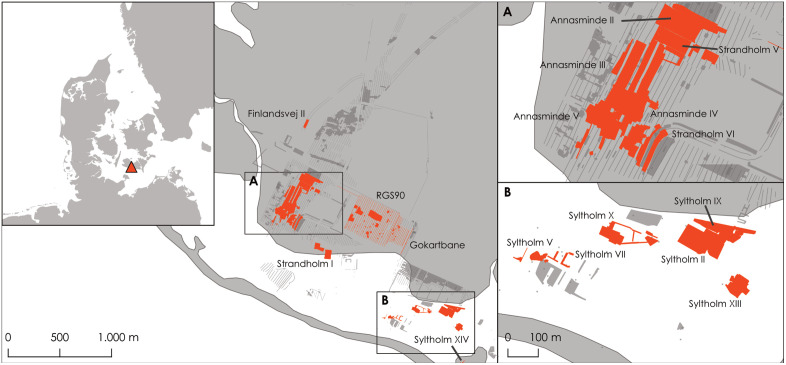
Location of the investigated sites. Investigated sites are shown in red. The background image illustrates the coastline in 1777 and indicates the approximate location of land (grey) and water (white) before the area was diked and reclaimed.

The Syltholm Fjord is a former fjord system on the Danish island of Lolland, which has been extensively excavated in the context of the Femern project, i.e., the archaeological investigation in connection with the construction of a submerged tunnel between Denmark and Germany [5,6] (Fig 1). A total excavation area of c. 57 ha in wet- and dryland areas revealed large archaeological assemblages spanning from the Late Palaeolithic to modern times, and which enable a wide range of analytical approaches [cf. 7]. Koivisto et al. [4,8,9] recently published analyses of stationary fishing structures used for many generations.

Studies of environmental effects on and exploitation of fish are numerous [e.g., 10–16]. Also from an archaeological perspective, similar topics have been addressed, even though these are generally focussed either on single sites [e.g., 17–22], national or superregional studies [e.g., 23–27], methodological aspects [e.g., 28–36], or specific artefacts connected to fishery [4, e.g., 37–40].

Here, we focus on the effects the sustained fishing pressure has had on the animal populations and potential consequences for human subsistence strategies in Syltholm Fjord between 4500–800 cal BCE, i.e., between the Late Mesolithic and Bronze Age. Hence the investigated timeframe covers the introduction of agriculture in Denmark around 4000 cal BCE and therewith a fundamental transformation of subsistence strategies. For contextualization, terrestrial fauna is included in the analysis and models are developed to evaluate the potential effect of fishing and animal husbandry for the subsistence strategies of the prehistoric population in the area.

## Materials and methods

All find assemblages under consideration were excavated in the context of developer-led archaeology. This means they are subject to selection biases with respect to representativity of excavated areas and the completeness of materials, as the building activity determined the location of trenches. Since a significant number of soil-samples were recovered and wet-sieved (mesh sizes: 2 and 0.5 mm), also bones of smaller species such as fish and rodents were recorded. All investigated assemblages may therefore be considered comparable in terms of preservation and representation of species (see below). Site specific differences due to preservation conditions and recovery methods cannot be excluded, not least because a part of the materials were recovered from wetland sites and some large volume sampling (big bags) of sediments has been done [cf. [5]]. Information on all sites and associated faunal assemblages is available in supplementary information (S1 Table).

The statistical analyses in this study are done in RStudio [41] using the packages lawstat [42], rcarbon [43], and vegan [44]. Additionally used packages can be found in the source codes added as supplementary information to this paper (S1 and S2 File).

Single date calibrations are done with OxCal 4.4 [45] with the IntCal20 calibration curve [46]. For sorting the sites chronologically, we use the median of the sites’ calibrated ages and k-means clustering from rcarbon and the R-core package, respectively (S3 Table for details).

### Faunal assemblages

Large parts of the faunal assemblages were found in waterlogged sediments in the former Syltholm Fjord [4,47,48]. Particularly finds discovered far from the former coastline of the Syltholm Fjord could therefore belong to a natural thanatocoenosis, whereas finds originating from the former coastal zone are likely to be refuse from nearby settlements. This is evident in many cases from their characteristic degree of fragmentation, cut and chop marks [49,50]. In younger periods, especially the Bronze Age, most investigated animal bones are recovered directly from settlement features, e.g., pits, and thus can be considered subject to human selection.

Since many fish bones from the waterlogged areas have been found in association with the excavations of stationary fishing structures or other anthropogenically deposited finds, like butchered animal bones, lithic and ceramic artefacts [4,50], we understand them as representative for the potentially targeted fish species. Similarities in the fish species composition between settlement areas and coastal zones can thus provide insight into selectivity or similarity in fishing practices. Bones from terrestrial animals are entirely interpreted as a result of human interaction.

We compiled data from 17 archaeological excavations from the Syltholm Fjord area ranging from the Late Mesolithic to the Bronze Age with a total number of identified specimens (NISP) of 8767 and a minimum number of individuals (MNI) of 472 animals (Table 1; see S1 Table for details). The assemblages were curated based on dating and availability of faunal data, as well as size of the faunal assemblages. Sites with NISPs below 30 were not regarded in this study and unidentified bones were excluded. The calculation of MNI is based on the identification information provided by the zooarchaeologists who performed the primary analysis under consideration of skeletal element, body side, and age, if MNIs were not calculated during the species identification. Due to the large deviations in skeletal element sizes and different numbers of skeletal elements, all fish MNI are set to 1, whenever detailed analyses were lacking and/or lateralization of singular elements was not possible.

**Table 1:** Overview of MNIs and NISPs per site.

SITE NAME	SITE ID	CHRONOLOGICAL GROUP (in this study)	MNI (excl. fish)	NISP	NISP Amphibians	NISP Birds	NISP Crustaceans	NISP Fish	NISP Mammals	NISP Marine mammals	NISP Reptiles
Annas Minde II	MLF01352	Late Neolithic	15	352		4		5	343		
Annas Minde III	MLF01353	Bronze Age	19	409				6	403		
Annasminde IV	MLF01354	Bronze Age	28	2216	8	1		1469	736		2
Annasminde V	MLF01355	Bronze Age	17	274				7	265	2	
Finlandsvej II	MLF02548	Bronze Age	13	120	2			7	111		
Gokartbane	MLF01333	Middle Neolithic	5	706				615	91		
RGS90	MLF00952	Bronze Age	9	50					50		
Strandholm I	MLF00909	Late Neolithic	30	251		15		10	221	5	
Strandholm V	MLF01182	Middle Neolithic	7	24					22	2	
Strandholm VI	MLF01232-I	Bronze Age	50	763				733	30		
Syltholm II	MLF00906	Late Mesolithic/Early Neolithic	151	2768	2	173	1	1007	1557	27	1
Syltholm IX	MLF00935	Middle Neolithic	47	372	1	11		99	257	4	
Syltholm V	MLF00910	Late Mesolithic/Early Neolithic	3	24				20	4		
Syltholm VII	MLF00933	Middle Neolithic	11	78		1		24	53		
Syltholm X	MLF00936	Late Mesolithic/Early Neolithic	21	168	1	21		27	101	18	
Syltholm XIII	MLF00939	Late Mesolithic/Early Neolithic	32	110	4	38	1	19	33	15	
Syltholm XIV	MLF00940	Middle Neolithic	14	82		2		2	78		
**SUM**			**472** **(≥ 518 with fish)**	**8767**	**18**	**266**	**2**	**4050**	**4355**	**73**	**3**

Minimum number of individuals (MNI) and numbers of identified specimens (NISP) for the faunal assemblages in this study.

Because the assemblages originate from different contexts, such as settlement features and littoral areas, this study primarily uses the NISP as the main quantitative measure, accounting for both the highly fragmentary nature of fish remains and differences in skeletal element representation. While MNIs are usually preferable for demographic analyses, the high degree of redundancy in fish skeletal elements as well as size differences of, for instance, vertebrae can render such calculations unreliable. Using NISP as a basis for calculations enables us to provide a replicable and comparable dataset across contexts and periods.

### Analyses

#### Salinity and Sediment.

Changes in the salinity of the Syltholm Fjord due to sea level changes [47] may have affected the species composition in the area of investigation independently of human influence. We therefore employed the FERI-index developed by Schmölcke and Ritchie [51, based on 52] with the aim of reconstructing salinity and sedimentary conditions. As archaeological fish assemblages can reflect both environmental conditions and selective fishing practices, we opted for a mixed model instead of using single indicator species for environmental reconstructions, since their occurrence may be influenced by human selection or scarcity in the assemblages [c.f. 53]. Since both salinity and prevailing sediments may have influenced species composition, we examine whether any long-term trends can be identified, as these may reflect ecological changes rather than shifts in fishing practices, which are likely to cause more rapid alterations in species composition [c.f. 54]. The FERI-values are translated into a binary matrix (S3 Table) to be able to apply a detrended correspondence analysis from the R-package vegan.

#### Diversity of faunal assemblages.

To evaluate potential ecological or cultural influences on assemblage composition, diversity indices are employed to distinguish random accumulation from patterned selection, as deviations from expected diversity may reflect underlying selective processes. For the diversity index analysis, we use the diversity index calculation from the R-package vegan with Simpson’s Index [55–57]. The results are displayed as values between 0 and 1 with higher values indicating a higher diversity. The index is calculated as


$$D\;=\;\text{1}-\sum_{i=\text{1}}^{S}\overset{\text{2}}{\underset{i}{p}}$$

where *p_i_* is the proportion of species *i*, *S* the number of species. The diversity index in the following is commonly given on a species group level (*D_group_*), i.e., ducks, cattle, roe deers, etc. Note that ovicaprids form a separate group from sheep such as goats if the species is undetermined as is true for domestic pigs and wild boar when it is unclear if they are domesticated or not. If not otherwise indicated, the NISPs are used as a calculation basis.

#### The Prey Choice Model.

Changes in subsistence patterns and ecological conditions are analyzed on the basis of diachronic datasets, which are also applied for establishing baseline species compositions in all investigated periods. To infer the relevance of wild prey in the human diet, we use prey choice models (PCMs) that incorporate all exploited species in the archaeological assemblages from the study area. This is of special relevance against the background of recent studies on the potentially masked contribution of fish to the diet of Early Neolithic societies [58,59].

The PCMs describe the relationship between prey energetic profitability and species representation at the sites, with a focus on the habitats from which these species are sourced. The models, loosely based on an optimal foraging theoretical framework [cf. 60], are used to evaluate the effects of hunting and fishing in different periods on resource availability and species composition, thereby improving our understanding of their contribution to human diets. Since PCMs originate in foraging theory they are designed to evaluate which resources a forager would select from a range of available prey types, under the assumption that foragers aim to maximize energetic returns.

Specifically, our model posits that decisions are based on post-encounter return rates (PERR), defined as net caloric gain per unit of handling time. Under optimal foraging conditions, when all prey types are equally accessible, higher-profitability species, i.e., those with greater caloric returns relative to handling costs, are expected to be selected more often and to dominate the archaeological assemblage [61–65].

We apply two approaches: one analysing continuous values of post-encounter return rate (PERR) and proportional abundance (%NISP; ‘*model a’*), and another one comparing their ranks (NISP rank; *‘model b’;* S2 File). The PCMs, especially *model b*, provide important insights into general trends of prey selection across sites and time periods. By focusing on the ordinal consistency between prey profitability and abundance, rather than exact quantities, this approach reveals broad subsistence patterns while minimizing the impact of outliers and sampling biases.

Due to long-term human activity in the Syltholm Fjord, including millennia of habitation and exploitation, the PCM is best applied as a flexible heuristic rather than a strict behavioral model. Many of the assemblages include domestic animals, whose presence reflects husbandry or provisioning rather than encounter-based foraging [e.g., 66], meaning the energetic optimization logic of the PCM does not fully apply to all taxa [67]. Instead, we use the model to rank taxa by profitability, providing a framework for assessing how exploitation patterns align with or diverge from efficiency-driven expectations. This approach helps to identify broader trends in human behaviour related to environmental pressure, habitat shifts, or cultural and economic preferences. For example, disproportionate representation of low-ranked taxa may indicate overexploitation of preferred habitats or a broadening of the resource base, whereas sustained reliance on high-ranked taxa could reflect abundance, ecological targeting, or selective cultural choice.

Although prey size and return rate are often positively correlated [68,69], large-bodied species can carry higher processing or handling costs, which in effect reduces their profitability [70–72]. Profitability in this study is calculated as:


$$PERR\;=\;\frac{e}{h}\;\times \;\text{6}\text{0}\;minutes$$

where *e* is the estimated caloric return (in kilocalories) and *h* is the handling time (in minutes). Following Yaworsky et al. [72] and excluding pursuit failure, we use nutrition values from published sources [71,73], as detailed in S1 Table.

Additionally to examining energetic profiles, we visualize species’ habitat preferences (labelled “biome” in the dataset; S1 Table) to better understand spatial dimensions of animal exploitation. Taxa are colour-coded according to their primary habitat – marine (“Marine,” “Mixed water,” “Mixed saltwater”), coastal (“Coastal,” “Freshwater,” “Wetland”), or terrestrial (“Forest,” “Mixed Land,” “Land”) – to illustrate where wild *and* domestic species were likely exploited within the broader landscape. While the PCM cannot capture all the complexities of a multi-strategy subsistence economy influenced by environmental change and human agency, it provides a valuable framework for interpreting faunal exploitation in terms of energetic return and habitat targeting.

### Dating

A wide range of sampled materials and radiocarbon dates allows for extensive temporal coverage, from c. 10000 cal BCE (the Late Palaeolithic) to 200 CE (the Roman Iron Age). Therefore, in our ecological analysis, it is possible to take a diachronic approach, considering the uncertainties and representativeness constraints inherent in samples selected primarily for archaeological dating. Consequently, some sites cover significant time spans, as they represent palimpsests or have been occupied over longer periods of time. In this analysis we use median dates to mitigate these dating uncertainties, assuming that the majority of the faunal assemblage is contemporaneous with the primary period of activity at a given site. This permits to order the assemblages chronologically, whereas detailed archaeological analysis remains necessary for more focused studies of settlement features, finds and structures [4, 49, 74, 75].

## Results

**Fig 2:**
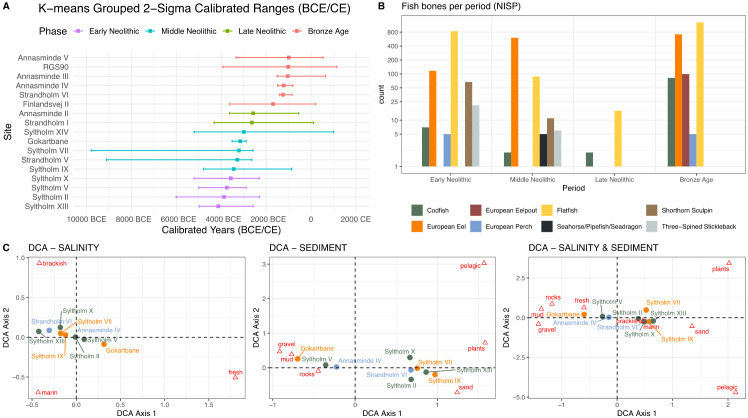
Diachronic overview of sites and salinity indexes. A: Chronological groups based on median values of radiocarbon dates. The four phases represent the Mesolithic/Neolithic transition and the Early Neolithic (“Early Neolithic”) and subsequent phases. B: Recorded fish species per time slice (NISP). Note that only species with a NISP> 4 are shown and that the y-axis is logarithmic in scale. C FERI analysis for salinity (left) and sediment (middle) and both combined (right) (eigenvalues salinity: 0.45 / < 0.01; sediment: 0.34 / 0.10).

We separated four different time slices which roughly represent (1) the Mesolithic/Neolithic transition and the Early Neolithic (“Early Neolithic”), c. 4500−3500 cal BCE, (2) the Middle Neolithic, c. 3500−2800 cal BCE, (3) the Late Neolithic, c. 2800−1800 cal BCE, and (4) the Bronze Age, c. 1800−800 cal BCE (Fig 2A).

### FERI analysis

For reconstructing the ecology of the fjord, we used assemblages with NISP > 14 as an iteratively selected threshold (Fig 2C). The sites do not show a pronounced trend of changing environmental conditions, despite expectations of a shift from brackish to marine conditions due to rising sea levels and changing salinity [47]. Instead, most sites cluster at intermediate conditions, without a tendency towards more salt- or freshwater conditions. The high number of eels in the Gokartbane assemblage causes a strong loading on the x-axis towards a freshwater signal. Generally, the results stand in line with coastal ecosystems with varying local influxes of freshwater.

The sedimentary analysis indicates differences in preferred fishing territories. A chronological trend cannot be identified, however, two clear clusters are visible: Some assemblages contain fish species that are more common in rocky and muddy sediments, whereas the others tend towards vegetation and sand-rich habitats. It is noteworthy for all sites of the latter group, but Strandholm VI, that they are found in the context of stationary fishing structures in the fjord, whereas the other assemblages originate from dryland settlement areas. Only the small Syltholm V-assemblage (n = 20) was recovered in the fjord.

The settlement assemblages, i.e., Gokartbane, Annasminde IV, Strandholm VI, display partly different sediment compositions than the assemblages from the fjord. The analysis is insofar inconclusive, as no clear trends based on location or dating can be seen in the dataset.

### Faunal assemblage composition

European eels (*Anguilla anguilla*; hereafter eel) and flatfishes (Pleuronectidae) are the dominant fish species in all assemblages (Fig 2B). While the numbers of other species vary over time, the dominant species remain unchanged. Among these are demersal species (Pleuronectidae, Cottidae) but also more pelagic fish (above all Gadidae; hereafter gadids).

Eels have been recovered from Neolithic and Bronze Age contexts with a marked dominance during the Middle Neolithic. This underscores the importance of anadromous fish for human subsistence during these periods and suggests that the shallow, lagoon-like environment likely provided favorable conditions for their capture. The marked decrease in fish bones during the Late Neolithic is best explained by a reduction in settlement activity at Syltholm Fjord, possibly due to environmental change [cf. 4]. Nevertheless, our dataset also demonstrates the continued or revived exploitation of fish during the Bronze Age. As most faunal remains from this period derive from settlement contexts, the identified fish species likely represent the catch composition.

For all periods, the relatively low number of pelagic fishes like gadids is worth noting. Since gadids are usually considered a species more adapted to deeper waters when they mature [cf. 34], their low presence might be a result of an unfavorable biotope in the shallow fjord, with water depths of < 3 m [48]. Although this taxonomic group reaches its highest numbers and relative proportions in the Bronze Age assemblages, the greater abundance of eel bones and the even higher absolute counts of flatfishes and eelpouts (Zooarcidae) suggest that gadids were not a primary targeted species. For three gadids the approximate lengths could be estimated, one specimen from the Late Mesolithic and Early Neolithic site Syltholm II and two from the Bronze Age site of Strandholm IV. All three specimens had reached c. 77 cm, c. 70 cm and c. 80 cm in total length, respectively. Since gadis of this size only rarely appear in small bays, these bones indicate fishing activity in deeper waters outside the fjord.

**Fig 3:**
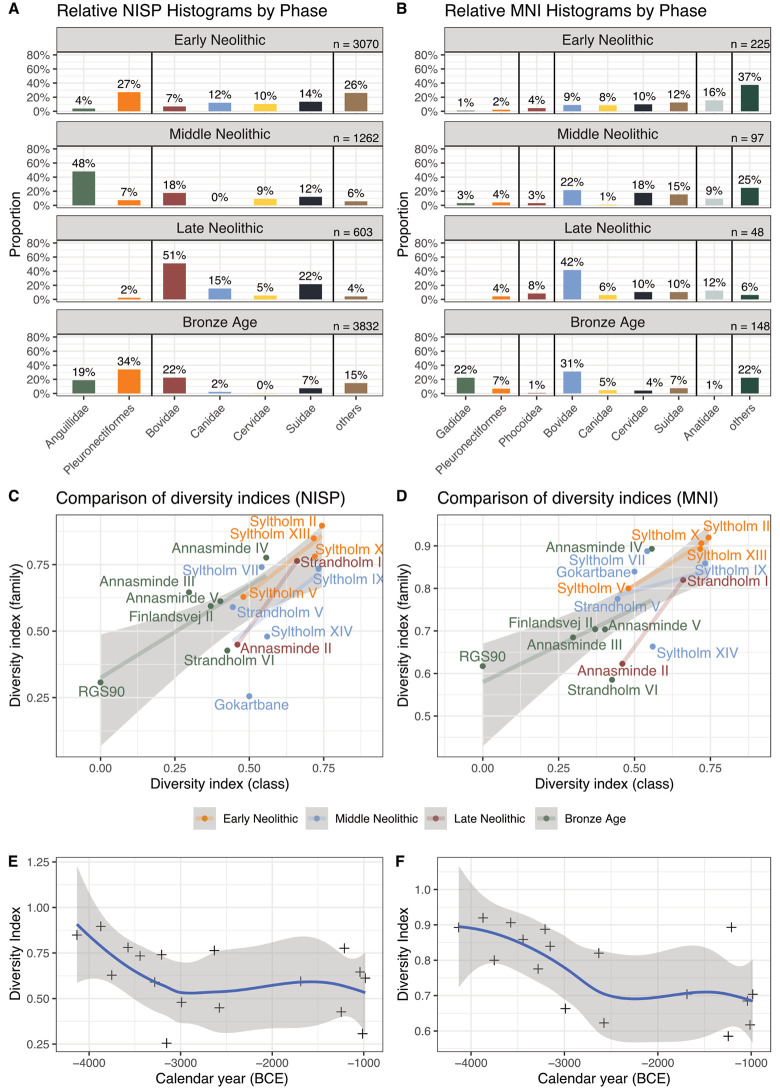
Proportion of identified animal bones per time-slice. Only species with an abundance ≥ 2.5% are shown with taxonomic group names. A: Based on NISP; B: based on MNI. Note that MNI for fish is set to 1 due to methodological reasons. C-D: Linear regression models with 95% confidence interval (grey ribbon) illustrate which site inventories show imbalances between taxonomic families and classes. Panel C shows calculation based on NISP and D based on MNI values. E-F: Changes in diversity indices over time. Crosses represent the median 14C-dates of the sites with a non-parametric regression curve (LOESS). Panel E for NISP and F for MNI.

But it is evident that fishing demersal species using stationary wooden structures has played a more significant role for the Bronze Age populations at Syltholm Fjord than potential deep-sea fishing with hook and line. In any case, fishing appears to have been an economically important activity during the Bronze Age, when cattle, sheep and goat dominated the terrestrial faunal assemblages. The fish fauna from this period is dominated by flatfishes and eels, whereas gadids represent only the fourth most abundant group (Figs 3A,B). Remains of seals and whales, in contrast, are only known from sites dating to the Early Neolithic time slice.

**Fig 4:**
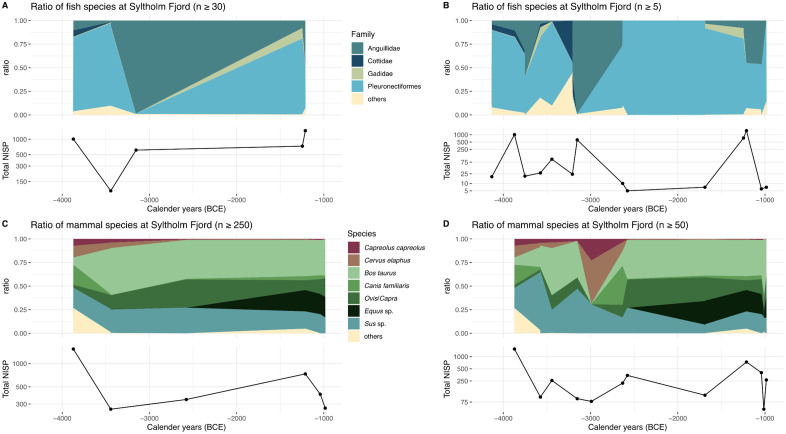
Diachronic overview of fish and mammal species in Syltholm Fjord. Only sites are included where the NISP for fish bones is **(A)** ≥ 5 and **(B)** ≥ 30 and for mammal bones **(C)** ≥ 250 and **(D)** ≥ 50. Figs 3A, B show the proportion of identified animal bones (NISP) and minimum number of individuals (MNI) per taxonomic family, including only species that represent ≥ 2.5% of the respective assemblage in each time slice. It is evident that fish consistently played a relevant role throughout the periods. The Late Neolithic assemblages are the only exception, with insufficient numbers of fish bones and a clear dominance of bovids.

As already mentioned, the number of fish bones in the assemblages decreases markedly in the Late Neolithic, mirroring the lower number of archaeological sites (cf. Figs 2A,B). Yet clear trends emerge in the composition of dominant species: Fig 4B illustrated the changing proportions of the four dominant fish taxa through time, based on taxonomic families recorded at least five times – that is, present in five or more assemblages during the study period. Although the low NISPs at some sites limit the reliability of the data, general patterns are apparent. The earlier sites are dominated by flatfishes, while eels become more frequent around 3800 cal BCE, followed by an increase in sculpins around 3400 cal BCE. Shortly thereafter, eels appear to dominate the assemblages until c. 2800 cal BCE, when flatfish, and to a lesser extent cod, again constitute about half of the total NISP. Overall, gadids never played a significant role in the Syltholm Fjord assemblages, whereas flatfishes remained the most prevalent species throughout most periods.

The pronounced dominance of eels around 3200 cal BCE warrants further investigation, especially since two other assemblages reinforce this trend. The dominance of eel in the Gokartbane assemblage likely skews the overall picture and may partly account for the species’ apparent overrepresentation. Generally, eel bones seem to occur in higher proportions in smaller assemblages (Fig 4B). When considering assemblages with more representative samples size (n ≥ 30) (Fig 4A), it becomes evident that the eel maximum is heavily driven by the extreme values from Gokartbane. Nevertheless, the small assemblages between c. 2600 and 1500 cal BCE suggest increased variability in the species composition.

The mammal bone assemblage shows a similarly dynamic pattern around 3000 cal BCE (Fig 4D). Wild species such as cervids (*Cervus elaphus*, *Capreolus capreolus*) increase noticeably at the expense of sheep/goats and cattle, while pigs decline slightly. It should be noted that wild and domestic pigs are often difficult to distinguish. By around 2550 cal BCE, horses emerge as a recurrent species, while cattle re-establish as the prevalent species in the assemblages. The rapid increase in wild game is primarily visible in smaller assemblages, whereas larger inventories mainly reflect an increase in ovicaprid bones until 4500 cal BP, after which their proportion decreases in favor of horses.

However, direct comparisons of fish and mammals are hardly useful due to the different nature and numbers of skeletal elements and potential selection biases during excavation. This becomes most apparent when the NISPs and MNIs are compared between both groups. While the proportions of NISP and MNI in the mammal group are roughly comparable, fish species differ clearly. This is to a large degree caused by methodological constraints, as all fish species were set to MNI = 1. We consider the NISP values therefore as better suited for comparisons.

To investigate the apparent variation in the exploited species spectrum, we compared diversity indices of taxonomic classes (i.e., mammals, birds, etc.) and taxonomic families (i.e., Suidae, Bovidae, etc.), based on the MNI and NISP per site (Figs 3C,D). Most sites are located within the 95% confidence interval of our regression model. Hence, they provide an expectable relation between taxonomic families and categories, indicating that the faunal spectrum is distributed according to statistical expectations. A linear relationship between sites from the same time slice shows that no relevant changes in the fauna spectrum can be observed on an inter-site level.

All sites from the Early Neolithic time slice fall within the expected diversity index range, whereas from the Middle Neolithic onwards, faunal assemblages become more diverse among themselves, as indicated by assemblages falling outside the confidence interval. It is also apparent that most Early Neolithic sites exhibit high diversity values, reflecting generally greater diversity in the animal bone assemblages. In contrast, Bronze Age sites are generally less diverse at both the family and class levels based on MNI, with the exception of Annasminde IV.

Some sites show a mismatch in their diversity values, suggesting that the assemblages represent a selective exploitation of animals. With respect to NISP, Gokartbane, Strandholm VI, Annasminde II, and Syltholm XIV stand out, as they all exhibit lower diversity at the family level, indicating dominance by a single taxonomic family. At the same time, their class-level diversity is higher than expected, reflecting a varied animal spectrum but intensive use of only a few species. In contrast, three sites (Syltholm VII, Annasminde III and IV) show slightly higher values, indicating a tendency toward more diverse species compositions than expected.

The diversity indices based on the MNI show a similar pattern but favour terrestrial fauna due to the inherent methodological constraints. This is clearly illustrated by the Gokartbane dataset, where the reduced dominance of fishbones in the faunal assemblage leads to a higher family diversity index.

When examining the changes in diversity over time (Figs 3E-F), it becomes evident that species diversity declined during the Early and Middle Neolithic and stabilized around 3000 cal BCE, based on NISPs. MNIs reached a relatively stable state from ca. 2500 cal BCE onwards, probably slightly delayed due to the influence of fish MNIs.

Taken together with the variety of fish and mammal species at Syltholm Fjord, these diversity changes are striking. After 3000 cal BCE, the relatively shallow diversity curve indicates a stable species composition, whereas the faunal inventories reveal a broader range of species.

### Prey choice models

**Fig 5:**
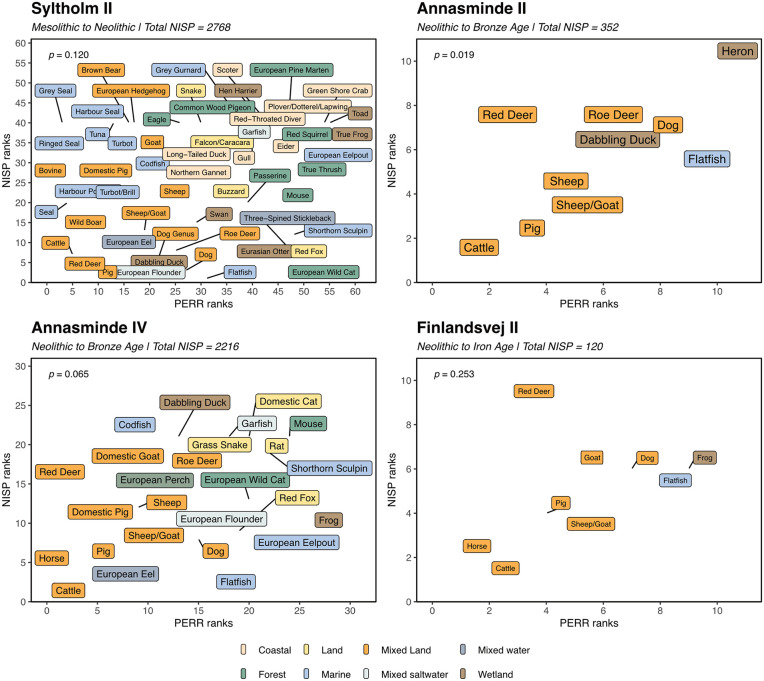
Prey Choice Models. Four selected sites from the Syltholm Fjord dataset are shown. Label colors indicate each species’ preferred general habitat or biome type. For Syltholm II, see Supplementary Information (S2 File) for an alternative version with terrestrial, coastal, and marine habitats separated into three distinct graphs to reduce overlap present in this combined view.

Building on the overall ecological and economic analyses, selected sites are highlighted to illustrate the results of the PCM analysis (Fig 5), while the full dataset and analysis is presented in S2 File. Site Syltholm II, spanning the Mesolithic–Neolithic transition, contains the largest and most taxonomically diverse assemblage in the dataset. Species originate from forest, wetland, marine, and coastal environments, and include both wild and domesticated species. Despite this diversity (*D_group_* = 0.896), the correlation between PERR and NISP ranks is weak and statistically non-significant (*p* = 0.120), indicating no clear structuring of prey choice based on energetic return. The presence of higher-ranked domestic species alongside a wide range of wild taxa instead shows a continued use of multiple ecological zones across the transition.

At Annasminde II, dating to the Late Neolithic cluster, the prey assemblage shows stronger structuring by profitability, with statistically significant correlation between PERR and NISP ranks (*p* = 0.019; *D_group_* = 0.449). While high-ranked domesticates such as cattle and sheep/goat dominate the assemblage, wild species from aquatic and wetland habitats, including fish and birds, are also present, though in low numbers.

At Annasminde IV, although model correlations are weaker (*p* = 0.053), the site presents a highly integrated economy combining intensive animal husbandry, extensive fishing, and opportunistic hunting (*D_group_* = 0.776). This site is a testament to a complex Bronze Age subsistence system which continued to exploit both terrestrial and marine resources alongside agriculture. A different pattern is seen at Bronze Age site Finlandsvej II, where the assemblage is overwhelmingly composed of terrestrial domestic species, including cattle, pig, sheep/goat, and dog (*D_group_* = 0.594). Aquatic exploitation is minimal, limited to occasional flatfish. The correlation between PERR and NISP ranks is not significant (*p* = 0.253), and the applicability of encounter-based return rates may be limited in such contexts due to the local availability of domestic resources.

These four sites illustrate diachronic variation in prey selection patterns at Syltholm Fjord. We can identify an early broad-spectrum exploitation of coastal, marine, and terrestrial habitats, followed by increasing reliance on high-ranked domestic taxa in later periods. At agricultural sites, where many key resources were managed and present at the settlements, the relevance of energetic profitability – especially in the sense of encounter-based decision-making – is conceptually different than in earlier contexts that are predominantly relying on a foraging lifestyle. However, our data also shows that even generations after adopting agriculture, the Syltholm Fjord and its hinterlands were exploited intensively for fish and terrestrial mammals. This is of special interest at the younger sites where red deer rank among the top species (Syltholm VII and XIV) in the model b-analysis and thus indicate a relevant contribution to the local diet through wild game hunt.

Only three sites (Annasminde II, Annasminde III, Syltholm X) produced statistically significant *p*-values for the correlations between PERR and NISP ranks, and thus a balanced number of animal bones and subsistence value. This shows that these other sites were keeping livestock not only based on economic, i.e., subsistence, values. Consequently, optimal foraging or production needs to be rejected as an explanatory model for these sites. Noteworthy are, however, the sites Syltholm II, X, and XIII, due to their comparably high numbers of seal bones, as well as porpoise in the case of Syltholm II. Nine directly dated specimens from these sites cluster between 4350 and 2910 cal BCE [see data in:  5,76]. Due to the fairly long span from the Late Mesolithic up until the Late Neolithic, hunting for marine mammals was potentially also done at Syltholm Fjord, however likely on an occasional but not regular basis.

## Discussion

Identifying prehistoric human impact on ecosystems and species compositions is a challenging task, as archaeological datasets are usually incomplete and non-representative. Examining faunal distributions, domestication processes, landscape changes, and hunting-strategies can reveal direct human impact on prehistoric fauna [e.g., 77–80]. By contrast, indirect human impacts like long-term biotope encroachment or loss, are more difficult to trace as the intentionality of anthropogenic activity must be evaluated against natural developments and successions [c.f. 81]. Therefore, reconstructing the local ecological baseline documenting the conditions in their natural state is a prerequisite for understanding potential impacts of human populations in a given area. Likewise, it must be noted that a stable ecological balance in nature only exists as long as important abiotic or biotic factors do not change – which can also happen naturally.

### Ecological indicators

The results of the FERI analysis indicated no significant changes in salinity, however, the sedimentary analysis points towards a difference of species in the assemblages recovered from the dryland (settlements) and waterlogged (fjord) contexts. Because the two assemblages originate from different areas (see Fig 1: area A and B), the possibility of localized fishing activity at the settlements cannot be entirely excluded. Such activity could account for the observed differences in sedimentary signals. Argumentatively, the location of the settlement sites on a small promontory further to the west potentially with a narrow confluence to the fjord might have even fostered fishing for specific species, like eels. Likewise, the confluence might have caused more erosion, so that the underwater habitats had a slightly different form. Fishing at more remote locations could also account for this pattern, but this is unlikely given the coastal location of the sites. Likewise, a difference in preservation conditions appears improbable, considering the comparability of the species compositions.

As of yet, no clear differences in the ecological conditions for the timeframes under question can be identified. While the changes in salinity and sea-level affected the Syltholm Fjord in the centuries before the Neolithic [47], our data indicates that changes in the aquatic milieu in the area were relatively stable from then on [cf. [48]].

A direct ecological impact of prehistoric human fishery, as species depletion or reduction in sizes, could not be proven in the available dataset. This shows that the millennia-long exploitation of aquatic resources and human presence in the area, can be understood as a long-standing relationship with the waterscape of the Syltholm Fjord [4,49]. The consistency in the targeted fish species and fishing methods indicates a well-developed and potentially sustainable fishery practice for several millennia.

### Use of wild and domestic resources

Although the animal species ratios between the different time slices show small changes over time, no substantial deviations have been observed. The most dominant fish species range between c. 30 and 50% in most periods, and bovid bones always dominate the terrestrial fauna. Comparing the proportions of species families shows a decline in faunal diversity over time, resulting in a significant difference between the Early Neolithic and Bronze Age time slices (t-test, *p* = 0.038). On the one hand, it appears that Bronze Age fishing was targeting similar species as in the earlier periods, with no recognisable change in species composition or fishing strategies [cf. 8]. On the other hand, the terrestrial fauna underwent changes in at least two periods. The increase of wild resources around 3000 cal BCE points to crucial changes in the subsistence strategy of people at Syltholm Fjord. The apparent reduction in the species spectrum coincides with an increased hunting and fishing, leading to noticeable deviations within the data. This short but pronounced resurgence of hunting fits chronologically with the presence of the Pitted Ware Culture in Denmark from c. 3100–2200 cal BCE [82–84] and the final Funnel Beaker ‘Store Valby phase’ between 3000 and 2600 cal BCE [85]. While the Pitted Ware Culture has not yet been attested on the southern Danish islands, a few isolated finds at Syltholm Fjord indicate wide-ranging contact networks during this period [82,86–88]. Current evidence is too sparse to suggest actual presence of Pitted Ware Culture elements at Syltholm Fjord, yet the increase of game in the faunal record around 3000 cal BCE coincides well with the apparent decline in the use of stationary fishing structures [4], hence indicating a shift in subsistence patterns. The following quite stable composition of terrestrial fauna and increasing frequency of horse bones after 2000 cal BCE is a good chronological fit with the start of the Bronze Age, when horses acquired a relevant socio-cultural role and became widespread in the local societies [89,90].

Contextualized human remains from the area are lacking, which complicates the reconstruction of human diet. Recent studies highlight challenges in isotope-based diet reconstructions [34,58], and evidence from Syltholm fjord pottery indicates the use of milk during the Final Mesolithic alongside with culinary practices that mixed aquatic and terrestrial foodstuffs [59,88]. The faunal assemblages analyzed here further support a mosaic-like subsistence pattern in the area during the Stone and Bronze Age. Diachronically, the dataset shows no trend toward the replacement of specific species, with marine resources continuing to contribute substantially to the diet. This finding challenges the recurrent notion that with “the onset of the Neolithic in Denmark, diet shifted abruptly to a dominance of terrestrial sources” [91]. The so-called Vittrup man [92], dating to ca. 3300–3100 cal BCE, is a good example for this: he consumed marine resources (fish and marine mammals) towards the end of his life, underscoring that aquatic resources played a role in the diet of Neolithic people.

**Fig 6:**
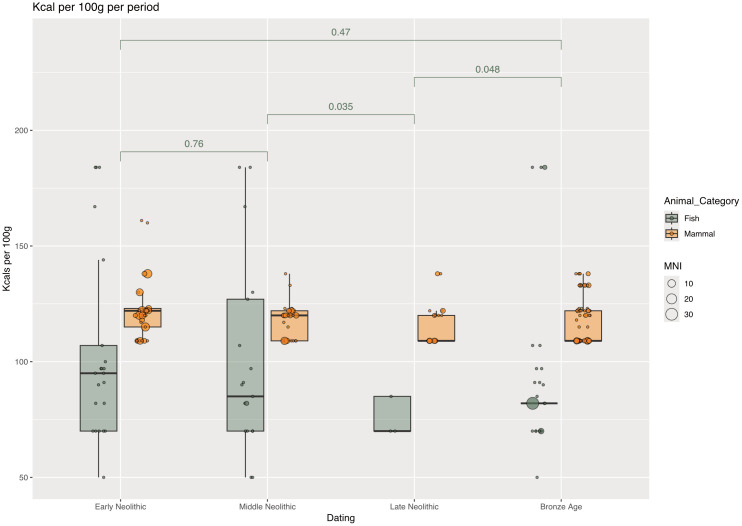
Diachronic comparison of nutritional values. Over the course of the Neolithic, the nutritional value of caught fish species slightly reduced (Basis MNI). *p*-values (t-test) are displayed for the energetic differences of the caught fish species between the time slices.

Regarding the nutritional value of fish caught in Syltholm Fjord, a statistically significant decline is observed toward the Late Neolithic, followed by an increase in the Bronze Age. This pattern is likely explained by the absence of eels, by far the most energy-dense species in the assemblages, at the end of the Neolithic. The only species present in substantial numbers are flatfish and cod, both of which are relatively lean. Overall, the species composition between the Early Neolithic and Bronze Age does not differ significantly (Fig 6). Consequently, there is no evidence for a shift in fishing strategies toward more energy-rich or lean species [see also [8]], nor for a substantial difference between Neolithic and Bronze Age assemblages. Together, these findings suggest a continuity in food acquisition practices in the area.

Recently Hinz [93] discussed Syltholm Fjord with respect to the Neolithization as a potential refugium [cf. 94], challenging the idea of a unilateral process for the introduction of agriculture. Our results support that subsistence strategies prevailed across the Neolithic transition. After agriculture was introduced to the area, fish, but also marine mammals, continued to play a substantial role for several millennia [4,8,9]. They underline that the unilateral focus on domestic crops as key-species for cultural change needs to be questioned. The prey choice models furthermore promote diversified perspectives on subsistence models with domestic and wild species, through consideration of handling costs and return rates of the exploited resources. Pollen and extensive macro-botanical analysis are still lacking for Syltholm Fjord [cf. 48, 95], so that the role of vegetable resources needs to remain another subject for future studies.

### Human Impact

Diversity in ecological communities is often enhanced by moderate disturbances in an ecosystem [96] and can thus potentially be used as an indirect measure for landscape changing effects in prehistory. In our example, the diversity index remains relatively stable after 3000 cal BCE, after a clear drop from ca. 4000 cal BCE, which can hence be understood as a decrease in moderate disturbances in a still forested landscape [48]. On the contrary, we assume that a more ‘stable ecosystem’ was established around 3000 cal BCE, potentially due to a millennium of human impact, resulting in a slightly reduced but maintained species spectrum in the faunal materials [96]. The less diverse and overall more homogeneous faunal assemblages (cf. Figs 3E, 4D), might in turn serve as an indicator of a more regulated social structure. *Sensu* Singleton and Taylor [97], we might see the transformation of the human communities at Syltholm Fjord reflected through the compositions of the faunal assemblages: Initially, the construction and maintenance of larger fish weirs provided mutual gains for the community. Hence the collective action of using the stationary fishing structures potentially regulated against overexploitation through mutual restraint or regulatory means [97]. The marked decrease in the use of fish weirs at Syltholm Fjord after c. 3000 cal BCE [4], coincides with a lower but rather stable diversity.

This pattern may be interpreted as an increase in political centralization against the backdrop of a transformed landscape, where moderate disturbances no longer affected the biological composition at a significant scale. In this context, the previously communal systems of resource acquisition and production likely became integrated into broader political–economic structures [96], leading to a homogenization of exploited fauna at the expense of biodiversity and local, community-based subsistence practices.

In short, the more diverse faunal assemblages around 4000 cal BCE may reflect both a more varied ecosystem and the presence of effective mechanisms to prevent overexploitation, particularly of fish resources, as exemplified by the larger stationary fishing structures. Over the following millennium, the landscape appears to have become increasingly homogenized, leading, on the one hand, to a reduction in the (wild) faunal spectrum and, on the other, to a potential rise in social complexity, together resulting in a more homogenic subsistence spectrum [cf. 97, 98]. Whether these developments are a result of overexploitation and a disentanglement of local communal management practices, or represent coincidental processes, remains a question for further research.

Nonetheless, they underline the interpretative challenges inherent in disentangling ecological change from social transformation in the archaeological record. Addressing such complexities requires a closer look at how human impact is evaluated and the methodological frameworks that underpin these assessments. Most indices for evaluating human impact are based on comparisons with a relative baseline state [cf. 99]. This poses a major challenge for reconstructing prehistoric human impact, given the scarcity and selectiveness of the archaeological record. Defining baseline state is equally demanding, as it should reflect the characteristics of an ecosystem or overall biodiversity before conclusions can be drawn about anthropogenic environmental change or human selection [cf. 100]. Consequently, employing several indicators and reconstructions, all with their specific scales, values, and statistical challenges, is a precondition for reliable evaluations [99, e.g., 100–103]. Evaluating human impact in archaeological assemblages requires careful consideration of the fragmentary nature of the datasets. Both the quality of preservation and the representativeness of individual periods vary considerably, affecting the comparability of the data [cf. 104]. Archaeological assemblages commonly represent different stages of exploitation and deposition and hence lack a uniform character. Additionally, the depositional and excavation contexts can significantly affect the composition of recovered materials, as both preservation conditions and selective discard processes influence what eventually enters the archaeological record [105].

The selectiveness of excavation methods, techniques and strategies play an additional substantial role in this context, henceforth influencing the recovery chances of specific finds but also their explanatory potential. Additionally, while archaeological data is pretty good with and focussed on identifying change [106,107], the identification of maintenance or ‘curation’ of wild resources is slightly more difficult – especially for aquatic species. Since monitoring human impact is challenging even in modern contexts, and because the aggregation of different and diverse indices or levels of information “would result in a hybrid and probably meaningless factor” [99], future studies on prehistoric human impact should adopt interdisciplinary frameworks that balance methodological coherence with analytical applicability.

Our combined approach, integrating an aquatic baseline signal with faunal inventories and PCMs, provides an initial insight into the long-term consequences of fisheries and landscape change at Syltholm Fjord. While no clear evidence of human impact on the fjord environment can be detected, changes in the terrestrial fauna suggest a reduction in biodiversity and, hypothetically, the emergence of more hierarchical or politically complex social structures.

### The relevance of local trajectories

Iversen [97] emphasizes with respect to material culture that “the 3rd millennium BC was a very heterogeneous period in south Scandinavian prehistory”, a pattern that appears to be reflected in the subsistence systems. The relevance of food and food wares as identifying elements in social groups must be considered when discussing the changes in the way-of-life of past societies [97,108,109]. Our data further suggest that faunal remains may carry traces of broader social transformations during the Mid- and Late Neolithic on Lolland.

The Neolithization at the turn of the millennium around 4000 cal BCE is assumed to have been an event of major scale that transformed societies suddenly and at large, so that the continued foraging lifeways at Syltholm Fjord are attributed to the “retreat of Mesolithic people into refugia” [93] and a “potential relict population of Mesolithic hunter-gatherers [... that …] had engaged on the periphery of communities of practise with the neighbouring agrarian groups” [94]. While the genetic (and to some extent) dietary change is interpreted by some as “robust evidence of demic diffusion” [91] our data supports a more nuanced perspective for changes within the Neolithic on Lolland [see also 4]. Similar arguments have been provided for the Netherlands, where Brusgaard et al. [110] recommend “that broad statements about the Neolithisation process north of the LBK zone should be reassessed and replaced with approaches that consider local trajectories”. We concur with this assessment, and our analysis underscores the importance of diversified, multithetic explanatory models for understanding social change in the past. The century-long effect of anthropogenic landscape change from the beginning of the Neolithic, and possibly earlier [but see 111], lead to a reduction in species diversity which might be sought in a more homogeneous landscape structure around Syltholm Fjord. Around 3000 cal BCE the faunal spectra were stable but exhibited low diversity, despite variations in species composition between sites. We interpret this as an additional indicator of the cultural transformations documented in eastern Denmark [98] – indirectly reflected in the faunal assemblages at Syltholm Fjord. Since our results suggest that ecological diversity was already declining prior to the influx of steppe ancestry at the onset of the Late Neolithic, we infer that extensive landscape modification and homogenization had likely occurred on Lolland before the arrival of new genotypes [91].

## Conclusion

In this contribution, we investigated whether the long-term use of the Syltholm Fjord system left detectable traces in the marine fauna. Analysis of aquatic and terrestrial faunal assemblages from the Late Mesolithic to the Bronze Age revealed clear temporal changes in faunal composition and diversity, but no evidence that sustained fishing pressure led to diversification of the fish fauna. Our results further indicate a shift from broad-spectrum foraging across coastal, marine, and terrestrial habitats toward increased reliance on domestic species over the course of the Neolithic, while wild resources, particularly aquatic fauna and red deer, remained important components of subsistence.

Although long-term fishing practices in the fjord did not produce archaeologically detectable ecological signals, the terrestrial fauna displayed more noticeable divergences. Notably, episodic increases in wild game around 3000 cal BCE, coinciding with reduced use of stationary fishing structures, may reflect broader regional developments in the Danish Neolithic, including the emergence of the Pitted Ware culture. We suggest that this period saw increased landscape homogenization, leading to reduced faunal diversity and potentially more centralized resources or social control. These patterns indicate that socio-economic developments during the Late Neolithic are indirectly reflected in the ecological conditions at Syltholm Fjord.

While future studies are needed to further explore the socio-economic dimensions of animal husbandry and material exchange in the area, our approach illustrates how multi-method analyses can reveal the subtle, long-term effects of prehistoric human activity on landscapes, offering a valuable tool for future studies of past human–environment dynamics.

## Supporting information

S1 Table Faunal data. (CSV)

S2 Table Binary matrix used for FERI analysis. (CSV)

S3 Table 14C data used for the chronological model. (CSV)

S1 File R-markdown of the ecological analyses. (HTML)

S2 File R-markdown of the Prey Choice models. (HTML)

S3 File Reference list for supporting information. (DOCX)
