# Supplementary material for: Fishing for millennia: Effects and impacts of prehistoric fishing in the Syltholm Fjord, Denmark
Source: PLoS One. 2026 May 13;21(5):e0347863. doi: 10.1371/journal.pone.0347863 (PMC13170857; doi:10.1371/journal.pone.0347863)
Supplement: S1 File — (HTML) [file pone.0347863.s004.html]

Supplementary Information S1 File - Ecological analysis for the prehistoric Syltholm Fjord


# Supplementary Information S1 File - Ecological analysis for the prehistoric Syltholm Fjord

#### Daniel Groß (https://orcid.org/0000-0002-1328-1134)

#### `2025

## R Markdown

This R Markdown file (**Supplementary Information S1
File**) contains a step-by-step guide to how we created the
ecological models for the paper **Fishing for millennia: Effects
and impacts of prehistoric fishing in the Syltholm Fjord,
Denmark**.

The HTML as well as the models (Figures 2-4 and 6 in the paper) are
made by co-author Daniel Groß.

# R Set up

## Used libraries

```
library(tidyverse)
library(vegan)
library(janitor) #crosstab function
library(ggpubr) #for statistics
library(lawstat)
library(ggsci) #use certain colour palette
library(ggrepel) #repel option for labels in ggplots
library(cowplot)
library(scales)
library(rcarbon)
```

## Setting up the workspace

```
rm(list = ls()) # remove global lists
gc()            # clean R memory

### SET WORKING DIRECTORIES ###
# getwd()
# setwd()

### Set up of additional vectors ###
#basic colour scheme
mlf_col <- c("#55725C", "#F98000", "#964b49", "#799ED1", "#FFD045", 
             "#252B33", "#967857", "azure3", "#264B41","#825671",  
             "bisque", "#35465C", "brown4", "darkgoldenrod",  "#D647A0", 
             "black")

#phase labels
phase_labels <- c(
  "EarlyNeo" = "Early Neolithic",
  "MiddleNeo" = "Middle Neolithic",
  "LateNeo" = "Late Neolithic",
  "BronzeAge" = "Bronze Age")
```

## loading the data and setting up the dataframes for the analysis

First we load the dataset and combine the radiocarbon dates with it.
All values in the main dataframe are changed to “1” if they are not
identified/not counted, to be able to calculate with them later on. The
modified dataframe is stored accordingly as “data\_MNI” and “data\_NISP”.
Note that the “cal\_data”-dataframe is already loaded here, so run
14C-analysis below first. Storing “cal\_data” already here is simply done
for reasons of clarity and comprehensibility of the code.

```
#load data and set up of dataframes
data <- read.csv2("S1_table.csv", header = TRUE)
cal_data <- read.csv2("site_summaries_cal.csv", header = TRUE)

#combine data with radiocarbon dates
data <- data %>%
  left_join(cal_data, by = c("Site_name" = "Site"))

# Replace all "indet" with "1" and ensure MNI is numeric
data_MNI <- data %>%
  mutate(across(where(is.character), ~ case_when(
    . == "indet" ~ "1",  # Replace "indet" with "1"
    TRUE ~ .            # Keep other values as is
  )),
  MNI = as.numeric(MNI),# Convert MNI to numeric
  MNI = ifelse(is.na(MNI), 1, MNI)  # Replace NA MNI with 1
  )

data_NISP <- data %>%
  mutate(across(where(is.character), ~na_if(., "indet")),
         NISP = as.numeric(NISP))
```

# 14C analysis

**RUN THIS FIRST** after setting up the workspace.

In this paper we use median values of radiocarbon ages for
chronological sorting of the sites. The “subset” stage is introduced if
larger datasets are used as basedata. If S3 Table is used, it is not
necessary.

```
data14C <- read.csv2("S3_table.csv", header = TRUE, , fileEncoding = "latin1")
data14C <- data14C[, -c(30:31)] #drop last two columns

#Subset only data that is relevant for the project.
data14C <- data14C %>% 
  subset(Site %in% c("Annasminde II", "Annasminde III", "Annasminde IV", "Annasminde V", "Finlandsvej II",
                     "Gokartbane", "RGS90", "Rødbyhavn Gokartbane", "Strandholm I", 
                     "Strandholm V", "Strandholm VI", "Syltholm II", "Syltholm IX", "Syltholm V",            
                     "Syltholm VII", "Syltholm X", "Syltholm XIII", "Syltholm XIV"))

site_ranges <- list()


# Get unique site names
unique_sites <- unique(data14C$Site)

# Loop through each site
for (site in unique_sites) {
  site_data <- subset(data14C,
                      Site == site &
                        !is.na(X14C.age) &
                        !is.na(X14C.STD) &
                        X14C.age > 200)  # filter young/invalid
  
  if (nrow(site_data) > 0) {
    cal <- calibrate(site_data$X14C.age,
                     site_data$X14C.STD,
                     calCurves = 'intcal20')
    
    sum_cal <- summary(cal)
    
    # Extract all TwoSigma ranges
    # Grab all numeric values from TwoSigma columns
    two_sigma_ranges <- unlist(regmatches(unlist(sum_cal[, grep("TwoSigma_BP_", names(sum_cal))]), gregexpr("\\d+", unlist(sum_cal[, grep("TwoSigma_BP_", names(sum_cal))]))))
    two_sigma_ranges <- as.numeric(two_sigma_ranges)
    
    if (length(two_sigma_ranges) >= 2) {
      lower <- min(two_sigma_ranges, na.rm = TRUE)
      upper <- max(two_sigma_ranges, na.rm = TRUE)
      median_date <- median(sum_cal$MedianBP, na.rm = TRUE)
      
      site_ranges[[site]] <- data.frame(
        Site = site,
        Median_CalBP = round(median_date),
        Lower_2sigma = round(lower),
        Upper_2sigma = round(upper)
      )
    } else {
      message("⚠️ No valid 2σ range for site: ", site)
    }
  } else {
    message("⚠️ No valid data for site: ", site)
  }
}

# Combine into a single data frame
result_df <- do.call(rbind, site_ranges)
#print(result_df)

# Make sure Site is treated as a factor and ordered (optional)
result_df$Site <- factor(result_df$Site, levels = result_df$Site[order(as.numeric(result_df$Median_CalBP, decreasing = TRUE))])

# Create a new phase column based on Median_CalBP
result_df$Phase <- cut(
  result_df$Median_CalBP,
  breaks = c(0, 3800, 4800, 5500, Inf),
  labels = c("Bronze Age", "Late Neolithic", "Middle Neolithic", "Early Neolithic"),
  right = FALSE
)
```

Now the sites are clustered into four chronological periods based on
their median dates.

```
## K MEANS CLUSTERING OF SITES
# Decide on number of clusters (e.g., 3 for Early, Middle, Late)
set.seed(17)  # For reproducibility
k <- 4

# Run k-means on the Median_CalBP column
kmeans_result <- kmeans(result_df$Median_CalBP, centers = k)

# Add cluster assignment to dataframe
result_df$Cluster <- as.factor(kmeans_result$cluster)
```

Additionally, we add the BCE/CE ages to the dataframe

```
#calculate BCE/CA dates
result_df$Median_CalBCAD <- 1950 - result_df$Median_CalBP
result_df$Lower_2sigma_BCAD <- 1950 - result_df$Lower_2sigma
result_df$Upper_2sigma_BCAD <- 1950 - result_df$Upper_2sigma
write.csv2(result_df, "site_summaries_cal.csv", row.names = FALSE)
```

## 14C plot

```
## `height` was translated to `width`.
```

## References for 14C dates

Additional references for 14C dates used in this paper.

- Bennike, O., Philippsen, B., Groß, D., Jessen, C., 2022. Holocene
  shore-level changes, southern Lolland and Femern Belt, Denmark. Journal
  of Quaternary Science 38, 440–451. https://doi.org/10.1002/jqs.3479
- Chaudesaigues-Clausen, S., 2023. Mesolithic persistence and
  Neolithic emergence at Syltholm II (MLF00906-III). Osseous artefacts
  before and after 4000 BCE on the coast of Lolland, Denmark, in: Groß,
  D., Rothstein, M. (Eds.), Changing Identity in a Changing World. Current
  Studies on the Stone Age in Northern Europe around 4000 Cal BC.
  Sidestone, Leiden, pp. 149–164.
- Dekker, J., 2025. Burnt to a crisp. Disentangling the taxonomic
  composition of foodcrusts via palaeoproteomics (Dissertation).
  University of Copenhagen, Copenhagen.
- Glykou, A., Lõugas, L., Piličiauskienė, G., Schmölcke, U.,
  Eriksson, G., Lidén, K., 2021. Reconstructing the ecological history of
  the extinct harp seal population of the Baltic Sea. Quaternary Science
  Reviews 251, 106701. https://doi.org/10.1016/j.quascirev.2020.106701
- Groß, D., Presslee, S., Schmölcke, U., Nikulina, E.A., Hendy, J.,
  2024. Danmark’s Not-So-Oldest Sheep: An Update on Domestic Animals from
  the Femern Project. Danish Journal of Archaeology 13, 1–10. https://doi.org/10.7146/dja.v13i1.145009
- Jensen, L.E., Jensen, S., Knöchel Christensen, A.V., Kring
  Mortensen, N.M., Deichmann, P.C.M., Måge, B., Mathiesen, A.-L.M.,
  Stafseth, T., 2016. Syltholmudgravningerne - jagten på stenalderens
  jægere, fiskere og bønder i et druknet landskab. Aarbøger for Nordisk
  Oldkyndighed og Historie 2015, 33–62.
- Jensen, T.Z.T., Niemann, J., Iversen, K.H., Fotakis, A.K.,
  Gopalakrishnan, S., Vagene, A.J., Pedersen, M.W., Sinding, M.S.,
  Ellegaard, M.R., Allentoft, M.E., Lanigan, L.T., Taurozzi, A.J.,
  Nielsen, S.H., Dee, M.W., Mortensen, M.N., Christensen, M.C., Sorensen,
  S.A., Collins, M.J., Gilbert, M.T.P., Sikora, M., Rasmussen, S.,
  Schroeder, H., 2019. A 5700 year-old human genome and oral microbiome
  from chewed birch pitch. Nature Communications 10, 5520. https://doi.org/10.1038/s41467-019-13549-9
- Jensen, T.Z.T., Sjöström, A., Fischer, A., Rosengren, E.,
  Lanigan, L.T., Bennike, O., Richter, K.K., Gron, K.J., Mackie, M.,
  Mortensen, M.F., Sørensen, L., Chivall, D., Iversen, K.H., Taurozzi,
  A.J., Olsen, J., Schroeder, H., Milner, N., Sørensen, M., Collins, M.J.,
  2020. An integrated analysis of Maglemose bone points reframes the Early
  Mesolithic of Southern Scandinavia. Scientific Reports 10. https://doi.org/10.1038/s41598-020-74258-8
- Koivisto, S., Robson, H.K., Philippsen, B., Stafseth, T., Brinch,
  M., Schmölcke, U., Astrup, P.M., Casati, C., Henriksen, M.B., Uldum, O.,
  Lundbye, M., Maring, R., Kanstrup, M., Måge, B.T., Groß, D., 2024.
  Fishing with stationary wooden structures in Stone Age Denmark: new
  evidence from Syltholm Fjord, southern Lolland. Proceedings of the
  Prehistoric Society 90, 147–176. https://doi.org/doi.org/10.1017/ppr.2024.15
- Måge, B., 2019. Syltholm VII: En atypisk beliggende
  Ahrensburglokalitet fra Syd-Lolland. Gefjon: arkæologi og nyere tid 4,
  176–193.
- Måge, B.T., Groß, D., Kanstrup, M., 2023. The Femern project: a
  large-scale excavation of a Stone Age landscape, in: Groß, D.,
  Rothstein, M. (Eds.), Changing Identity in a Changing World. Current
  Studies on the Stone Age in Northern Europe around 4000 Cal BC.
  Sidestone, Leiden, pp. 21–32.
- Philippsen, B., 2018. Reservoir Effects in a Stone Age Fjord on
  Lolland, Denmark. Radiocarbon 60, 653–665. https://doi.org/10.1017/rdc.2018.6
- Sørensen, S.A., 2020. Ritual depositions in the coastal zone: A
  case from Syltholm, Denmark, in: Schülke, A. (Ed.), Coastal Landscapes
  of the Mesolithic. Human Engagement with the Coast from the Atlantic to
  the Baltic Sea. Routledge, London / New York, pp. 394–414.

# Descriptive statistics

## FISH SPECIES PER PERIODS

```
# summarize different species into groups #
data_div_Cods <- data %>%
  filter(Animal_Category == "Fish", NISP > 4) %>%
  complete(Phase, Common_name, fill = list(NISP = 0)) %>%  # Ensure every combination is present
  mutate(
    Common_name = recode(Common_name, 
                         "Atlantic Cod" = "Codfish",   # Replace "Atlantic Cod" with "Codfish"
                         "European Flounder" = "Flatfish",  # Replace "European flounder" with "Flatfish"
                         "Turbot/Brill" = "Flatfish"),  # Replace "Turbot/Brill" with "Flatfish"
    NISP = ifelse(NISP == 0, 1, NISP)  # Replace NISP value of 0 with 1 for log scale compatibility
  )%>%
  group_by(Phase, Common_name) %>%   # <-- Summarise duplicates
  summarise(NISP = sum(NISP), .groups = "drop")

#add chronological phases to the dataframe
data_div_Cods$Phase <- factor(
  data_div_Cods$Phase,
  levels = c("Early Neolithic",  "Middle Neolithic", "Late Neolithic","Bronze Age")
)
```

## FERI analysis

The FERI analysis is used for identifying changes in fauna and
sediment.

### data preparation

We created a template for the different fish species after Schmölcke
and Ritchie (2010; based on Froese and Pauly, 2000). After loading the
FERI values, we combine them with the faunal dataset and rename it to
“site\_species\_data” which will subsequently be used.

```
env_data <- read.csv2("S2_table.csv", header = TRUE)
colnames(env_data)[colnames(env_data) == 'Species'] <- 'Latin_name'

# Environmental data (from merged dataset)
env_data[is.na(env_data)] <- 0
env_data[, -1] <- lapply(env_data[, -1], as.numeric) # Ensure all environmental columns are numeric

site_species_data <- data %>%
  filter(Animal_Category == 'Fish') %>%   # Use `filter()` instead of `subset()`
  select(Common_name:Family, Site_name:Period, NISP, MNI, Median_CalBP:Cluster) %>%  
  mutate(Latin_name = ifelse(Latin_name %in% c("P. platessa/P. flesus/L. limanda","Platichthys sp./L. limanda", "Psetta maxima/S. rhombus"),  #rename flatfishes
                             "Pleuronectoideo", 
                             Latin_name)) %>%
  left_join(env_data, by = "Latin_name")  # Match species by name

#set all MNI that are "indet" to 1 (as integer)
site_species_data <- site_species_data %>%
  mutate(MNI = if_else(MNI =="indet", 1L, as.integer(MNI)))
```

```
## Warning: There was 1 warning in `mutate()`.
## ℹ In argument: `MNI = if_else(MNI == "indet", 1L, as.integer(MNI))`.
## Caused by warning in `if_else()`:
## ! NAs introduced by coercion
```

```
# Convert site-species data into a species abundance matrix
site_species_matrix <- site_species_data %>%
  group_by(Site_name, Latin_name) %>%
  summarise(NISP = sum(NISP, na.rm = TRUE), .groups = "drop") %>%  # <- drop grouping!
  spread(Latin_name, NISP, fill = 0) %>%
  mutate(row_sum = rowSums(across(where(is.numeric)))) %>%   # # safer numeric-only selection
  filter(row_sum > 14) %>%                              #KEEP ONLY NISP >=15
  select(-row_sum) %>%                                
  column_to_rownames("Site_name") 

# Matrix multiplication to get weighted mean trait values per site
rownames(env_data) <- env_data$Latin_name  # Set row names as Latine names (assuming first column is site_ID)
env_data <- env_data[,-c(1:3)]   # Remove the site_ID column (and the two FERI index columns) from the dataframe

env_data <- na.omit(env_data[colnames(site_species_matrix), , drop = FALSE])
```

In the FERI analysis we only keep sites which have a NISP >= 15.
And calculate the environmental parameters

```
#Step 1: Convert Data to Numeric Matrices
site_species_matrix <- as.matrix(site_species_matrix)  # Convert to numeric matrix
env_data <- as.matrix(env_data)  # Convert to numeric matrix

#Step 2: CHECK if datasets are comparable
all(colnames(site_species_matrix) %in% rownames(env_data))  # Should return TRUE
all(rownames(env_data) %in% colnames(site_species_matrix))  # Should return TRUE
site_env_data <- site_species_matrix %*% env_data  # Matrix multiplication

#Step 3: Compute Site-Level Environmental Data
site_env_data <- site_species_matrix %*% env_data  # Matrix multiplication

site_env_data <- as.data.frame(site_env_data)#convert to dataframe
rownames(site_env_data) <- rownames(site_species_matrix)  # Ensure site names remain
```

Now we merge datasets to be able to display site names, periods, and
cluster number.

```
#Step 0: Add the corresponding fields to data_sites
data_sites <- as.data.frame(table(data$Site_name, data$Period_red, data$Cluster)) %>%  
  magrittr::set_colnames(c("Site", "dating", "cluster","count")) %>%  # Rename columns
  filter(count > 0) %>%                                    # Remove zero rows
  group_by(Site, cluster) %>%
  summarize(Dating_Info = paste(unique(dating), collapse = ", "), .groups = "drop") # Drop groups to avoid warnings

# Step 1: Ensure "Site" is an explicit column in site_env_data
site_env_data <- site_env_data %>%
  tibble::rownames_to_column(var = "Site") %>%  
  left_join(data_sites, by = "Site") %>%  # Left join with data_sites
  mutate(cluster = replace_na(as.character(cluster), "99")) %>%  # Replace NA in 'cluster' with "99"
  tibble::column_to_rownames(var = "Site")  # Convert "Site" back to rownames
```

Finally we create a function to apply a Detrended Correspondence
Analyses and include a command to display the analyses. This way we can
just call the function with according parameters to reduce
repetition.

```
# Function to perform DCA analysis and plotting
perform_dca_analysis <- function(data, env_cols, species_map, title, mlf_col_ca) {
  
  # Step 1: Detrended Correspondence Analysis (DCA)
  res_ca <- decorana(data[, env_cols]) 
 
  # Step 2: Extract site scores and ensure Site is an explicit column
  dca_scores <- as.data.frame(scores(res_ca, display = "sites")) %>%
    tibble::rownames_to_column(var = "Site")
  
  # Step 3: Extract species scores, rename species, and ensure species is a column
  dca_scores_species <- as.data.frame(scores(res_ca, display = "species")) %>%
    tibble::rownames_to_column(var = "species") %>%
    mutate(species = recode(species, !!!species_map))  # Rename species
  
  # Step 4: Merge "Dating_Info" from site_env_data into dca_scores
  dca_scores <- dca_scores %>%
    left_join(data %>% tibble::rownames_to_column(var = "Site") %>%
                select(Site, cluster), by = "Site")      ### CHANGE HERE FOR Dating_Info for CLUSTER

  # Step 5: Create ggplot visualization
 p <- ggplot(data = dca_scores, aes(x = DCA1, y = DCA2, label = Site, color = cluster, fill = cluster)) +
    geom_point(size = 3, shape = 21) +  # Use shape 21 (filled circles)
    #stat_ellipse(aes(group = cluster), level = 0.95, linetype = "solid", size = 0.8) +  # Confidence ellipse
    geom_text_repel(size = 3, box.padding = 0.3, point.padding = 0.2, max.overlaps = Inf) +  
    geom_hline(yintercept = 0, color = "black", linetype = "dashed") +  
    geom_vline(xintercept = 0, color = "black", linetype = "dashed") +  
    theme_bw() +  # Apply cleaner theme
    scale_fill_manual(values = mlf_col_ca, breaks = c("1" , "2", "3" , "4", "5", "99")) +  # Apply custom fill colors
    scale_color_manual(values = mlf_col_ca, breaks = c("1" , "2", "3" , "4", "5", "99")) +  # Ensure color matches fill
    labs(
      title = paste("DCA -", title),
      x = "DCA Axis 1",
      y = "DCA Axis 2"
    ) +
    geom_point(data = dca_scores_species, aes(x = DCA1, y = DCA2), inherit.aes = FALSE, 
               color = "red", size = 2, shape = 2) +  
    geom_text_repel(data = dca_scores_species, aes(x = DCA1, y = DCA2, label = species), 
                    inherit.aes = FALSE, color = "red", size = 3)+
   theme(legend.position = "none")
 return(p)  # Return the plot
 
 }
```

And now we perform the analysis and safe the results for later
display (for the figures see main article).

```
# Define species renaming maps
FERI_map <- c("SAL_marine" = "marin", "SAL_brackish" = "brackish", "SAL_fresh" = "fresh",
"SED_pelagic" = "pelagic", "SED_mud" = "mud", "SED_rocks" = "rocks",
                  "SED_sand" = "sand", "SED_gravel" = "gravel", "SED_plants" = "plants")

# Define color palette
mlf_col_ca <- c( "1" = "#55725C", "3" = "#F98000", 
                 "2" = "#964b49", "4" = "#799ED1", "5" = "#252B33", "99"="#825671")  

# Run the function for SALINITY
p_FERIsal <- perform_dca_analysis(site_env_data, 1:3, FERI_map, "SALINITY", mlf_col_ca)

# Run the function for SEDIMENT
p_FERIsed <-perform_dca_analysis(site_env_data, 4:9, FERI_map, "SEDIMENT", mlf_col_ca)

# Run the function for both SALINITY and SEDIMENT
p_FERI <- perform_dca_analysis(site_env_data, 1:9, FERI_map, "SALINITY & SEDIMENT", mlf_col_ca)
```

# Faunal assemblage composition

First we create a function to easily filter the faunal assemblages
based on their total NISP number per site. We start with fish. See main
article for figures.

## Fish

```
#group species by median bp
create_filtered_data <- function(min_total_nisp = 1) {
  data %>%
    filter(Animal_Category == "Fish") %>%
    mutate(Family = if_else(Family == "Pleuronectidae", "Pleuronectiformes", Family)) %>%
    add_count(Family, name = "FamilyCount") %>%
    mutate(Family = if_else(FamilyCount < 5, "OTHERS", Family)) %>%
    select(-FamilyCount) %>%
    group_by(Median_CalBCAD, Animal_Category, Family) %>%
    summarise(total_NISP = sum(NISP, na.rm = TRUE), .groups = "drop") %>%
    complete(Median_CalBCAD, Family, fill = list(total_NISP = 0)) %>%     # Fill missing Family–Median_CalBP combinations with 0
    group_by(Median_CalBCAD) %>%
    filter(sum(total_NISP) >= min_total_nisp) %>%
    ungroup() %>%
    mutate(Family = factor(
      Family,
      levels = c("Anguillidae", "Cottidae", "Gadidae", "Pleuronectiformes", "OTHERS")
    ))
}
```

The function is applied to store different NISPs.

```
filtered_data_1 <- create_filtered_data(min_total_nisp = 1)
filtered_data_5 <- create_filtered_data(min_total_nisp = 5)
filtered_data_30 <- create_filtered_data(min_total_nisp = 30)
```

### Figures

```
#Top plot: display ratios 
p_diafish1a <- ggplot(filtered_data_5, aes(x = Median_CalBCAD, y = total_NISP, fill = Family)) +
  geom_area(position = position_fill()) +
  theme_bw()+
  scale_fill_manual(values = c(
    "Anguillidae" = "#488286" , "Cottidae" = "#1B4965","Gadidae"="#BECC9F", "Pleuronectiformes" = "#62B6CB",  
    "OTHERS" = "#ffedc4")
  )+
  labs(
    title = "Ratio of fish species at Syltholm Fjord (n ≥ 5)",
    x = NULL,
    y = "ratio"
  ) + 
  scale_x_reverse()+
  xlim(c(-4200, -950))+
  theme(
    axis.title.x = element_blank(),
    axis.text.x = element_blank(),
    axis.ticks.x = element_blank(),
    panel.border = element_blank(),       # removes box around plot area
  )
```

```
## Scale for x is already present.
## Adding another scale for x, which will replace the existing scale.
```

```
# Bottom plot: absolute total NISP
total_nisp_plot_data <- filtered_data_5 %>%
  group_by(Median_CalBCAD) %>%
  summarise(total_NISP = sum(total_NISP, na.rm = TRUE))

p_diafish1b <- ggplot(total_nisp_plot_data, aes(x = Median_CalBCAD, y = total_NISP)) +
  geom_line(color = "black") +
  geom_point(color = "black") +
  theme_bw() +
  labs(
    x = "Calender years (BCE)",
    y = "Total NISP"
  ) +
  scale_x_reverse()+
  xlim(c(-4200, -950))+
  scale_y_log10(
    breaks = c(5,10,25,75,250,500,1000)
  )+
  theme(panel.border = element_blank(),
        panel.grid.minor.y = element_blank())
```

```
## Scale for x is already present.
## Adding another scale for x, which will replace the existing scale.
```

```
p_diafish2a <- ggplot(filtered_data_30, aes(x = Median_CalBCAD, y = total_NISP, fill = Family)) +
  geom_area(position = position_fill()) +
  theme_bw()+
  scale_fill_manual(values = c(
    "Anguillidae" = "#488286" , "Cottidae" = "#1B4965", "Gadidae"="#BECC9F", "Pleuronectiformes" = "#62B6CB",  
    "OTHERS" = "#ffedc4")
  )+
  labs(
    title = "Ratio of fish species at Syltholm Fjord (n ≥ 30)",
    x = NULL,
    y = "ratio"
  ) + 
  scale_x_reverse()+
  xlim(c(-4200, -950))+
  theme(
    axis.title.x = element_blank(),
    axis.text.x = element_blank(),
    axis.ticks.x = element_blank(),
    panel.border = element_blank(),       # removes box around plot area
    legend.position = "none"
  )
```

```
## Scale for x is already present.
## Adding another scale for x, which will replace the existing scale.
```

```
# Bottom plot: absolute total NISP
total_nisp_plot_data <- filtered_data_30 %>%
  group_by(Median_CalBCAD) %>%
  summarise(total_NISP = sum(total_NISP, na.rm = TRUE))

p_diafish2b <- ggplot(total_nisp_plot_data, aes(x = Median_CalBCAD, y = total_NISP)) +
  geom_line(color = "black") +
  geom_point(color = "black") +
  theme_bw() +
  labs(
    x = "Calender years (BCE)",
    y = "Total NISP"
  ) +
  scale_x_reverse()+
  xlim(c(-4200, -950))+
  scale_y_log10(
    breaks = c(3,5,10,25,50,75,150,300,500,1000)
  )+
  theme(panel.border = element_blank(),
        panel.grid.minor.y = element_blank())
```

```
## Scale for x is already present.
## Adding another scale for x, which will replace the existing scale.
```

## Mammals

Same analysis as before, but for mammals.

```
create_filtered_data_MAMMALS <- function(min_total_nisp = 1) {
  data %>%
    filter(Animal_Category == "Mammal") %>%
    mutate(
      Latin_name = if_else(Latin_name %in% c("Ovis aries", "Ovis/capra", "Capra hircus"), "Ovis/Capra", Latin_name),
      Latin_name = if_else(Latin_name == "Equus caballus", "Equus sp.", Latin_name),
      Latin_name = if_else(Latin_name %in% c("Sus domesticus", "Sus scrofa"), "Sus sp.", Latin_name)
    ) %>%
    filter(Latin_name != "Muridae") %>%
    add_count(Latin_name, name = "FamilyCount") %>%
    mutate(Latin_name = if_else(FamilyCount < 5, "OTHERS", Latin_name)) %>%
    select(-FamilyCount) %>%
    group_by(Median_CalBCAD, Animal_Category, Latin_name) %>%
    summarise(total_NISP = sum(NISP, na.rm = TRUE), .groups = "drop") %>%
    complete(Median_CalBCAD, Latin_name, fill = list(total_NISP = 0)) %>%
    group_by(Median_CalBCAD) %>%
    filter(sum(total_NISP) >= min_total_nisp) %>%
    ungroup()%>%
    mutate(Latin_name = factor(
      Latin_name,
      levels = c( "Capreolus capreolus", "Cervus elaphus", 
                  "Bos taurus", "Canis familiaris","Ovis/Capra", "Equus sp.","Sus sp.", "OTHERS")
    ))
}

filtered_data_1 <- create_filtered_data_MAMMALS(min_total_nisp = 1)
filtered_data_50 <- create_filtered_data_MAMMALS(min_total_nisp = 50)
filtered_data_250 <- create_filtered_data_MAMMALS(min_total_nisp = 250)
```

### Figures

See Fig. 4 in main article.

```
p_diamam1a <- ggplot(filtered_data_50, aes(x = Median_CalBCAD, y = total_NISP, fill = Latin_name)) +
  geom_area(position = position_fill()) +
  theme_bw()+
  labs(
    title = "Ratio of mammal species at Syltholm Fjord (n ≥ 50)",
    x = NULL,
    y = "ratio"
  ) + 
  scale_x_reverse()+
  xlim(c(-4200, -950))+
  theme(
    axis.title.x = element_blank(),
    axis.text.x = element_blank(),
    axis.ticks.x = element_blank(),
    panel.border = element_blank(),       # removes box around plot area
  )+
  scale_fill_manual(values =c("#82324b","#9c755c",  "#97C794", "#5f9c56","#3c6d3d","#0a1f09", "cadetblue","#ffedc4" ))
```

```
## Scale for x is already present.
## Adding another scale for x, which will replace the existing scale.
```

```
# Bottom plot: absolute total NISP
total_nisp_plot_data <- filtered_data_50 %>%
  group_by(Median_CalBCAD) %>%
  summarise(total_NISP = sum(total_NISP, na.rm = TRUE))

p_diamam1b <- ggplot(total_nisp_plot_data, aes(x = Median_CalBCAD, y = total_NISP)) +
  geom_line(color = "black") +
  geom_point(color = "black") +
  theme_bw() +
  labs(
    x = "Calender years (BCE)",
    y = "Total NISP"
  ) +
  scale_x_reverse()+
  xlim(c(-4200, -950))+
  scale_y_log10(
    breaks = c(5,10,25,75,250,500,1000)
  )+
  theme(panel.border = element_blank(),
        panel.grid.minor.y = element_blank())
```

```
## Scale for x is already present.
## Adding another scale for x, which will replace the existing scale.
```

```
p_diamam2a <- ggplot(filtered_data_250, aes(x = Median_CalBCAD, y = total_NISP, fill = Latin_name)) +
  geom_area(position = position_fill()) +
  theme_bw()+
  labs(
    title = "Ratio of mammal species at Syltholm Fjord (n ≥ 250)",
    x = NULL,
    y = "ratio"
  ) + 
  scale_x_reverse()+
  xlim(c(-4200, -950))+
  theme(
    axis.title.x = element_blank(),
    axis.text.x = element_blank(),
    axis.ticks.x = element_blank(),
    panel.border = element_blank(),       # removes box around plot area
    legend.position = "none"
  )+
  scale_fill_manual(values =c("#82324b","#9c755c",  "#97C794", "#5f9c56","#3c6d3d","#0a1f09", "cadetblue","#ffedc4"))
```

```
## Scale for x is already present.
## Adding another scale for x, which will replace the existing scale.
```

```
# Bottom plot: absolute total NISP
total_nisp_plot_data <- filtered_data_250 %>%
  group_by(Median_CalBCAD) %>%
  summarise(total_NISP = sum(total_NISP, na.rm = TRUE))

p_diamam2b <- ggplot(total_nisp_plot_data, aes(x = Median_CalBCAD, y = total_NISP)) +
  geom_line(color = "black") +
  geom_point(color = "black") +
  theme_bw() +
  labs(
    x = "Calender years (BCE)",
    y = "Total NISP"
  ) +
  scale_x_reverse()+
  xlim(c(-4200, -950))+
  scale_y_log10(
    breaks = c(3,5,10,25,50,75,150,300,500,1000)
  )+
  theme(panel.border = element_blank(),
        panel.grid.minor.y = element_blank())
```

```
## Scale for x is already present.
## Adding another scale for x, which will replace the existing scale.
```

# Species ratios and Diversity indexes

The Simpson’s diversity index is calculated based on number of
individuals per species. First we create a function to be able to run
the analysis easily for different groups of data.

## Diversity index function

```
# Function to calculate Simpson's Diversity Index based on mentions of a species
 # If no weighting column is provided, default to a column of ones

calculate_diversity <- function(data, grouping_column, weighting_column=NULL) {
  if (is.null(weighting_column)) {
    data <- data %>%
      mutate(Weight = 1)  # Add a default Weight column
    weighting_column <- sym("Weight")
  } else {
    weighting_column <- sym(weighting_column)
  }
  
  # Convert column names to symbols for tidy evaluation
  grouping_column <- sym(grouping_column)
  weighting_column <- sym(weighting_column)
  
  # Tabulate data using the weighting column for values
  tabulated <- data %>%
    group_by(Site_name, !!grouping_column) %>%
    summarise(Weight = sum(!!weighting_column, na.rm = TRUE), .groups = "drop") %>%
    pivot_wider(names_from = !!grouping_column, values_from = Weight, values_fill = 0)
  
  # Set row names and remove the first column
  site_column  <- tabulated$Site_name
  tabulated <- tabulated[, -1]
  
  # Calculate diversity index using weights
  tabulated$DIV_index <- diversity(as.matrix(tabulated), index = "simpson")
  
  # Add Site_name back to the result
  tabulated <- cbind(Site_name = site_column, tabulated)
  return(tabulated)
}
```

Application of the Diversity Index function to different data
sets.

```
# Calculate diversity for Family
data_MNI.group <- calculate_diversity(data_MNI, grouping_column = "Family", weighting_column =  "MNI")
data_NISP.group <- calculate_diversity(data_NISP, grouping_column = "Family", weighting_column =  "NISP")

# Calculate diversity for Animal Category/Class
data_AniCat.NISP <- calculate_diversity(data_NISP, "Animal_Category")
data_AniCat.MNI <- calculate_diversity(data_MNI, "Animal_Category")

# Create a dataframe for sites and dating information
data_sites <- as.data.frame(table(data$Site_name, data$Phase)) %>%  
magrittr::set_colnames(c("Site", "dating", "count")) %>%  # Rename columns
  filter(count > 0) %>%                                    # Remove zero rows
  group_by(Site) %>%
  summarize(Dating_Info = paste(unique(dating), collapse = ", "), .groups = "drop") # Drop groups to avoid warnings

# Combine rownames_vector and diversity index columns
data_DIV.comb <- data.frame(
  Site = data_MNI.group$Site_name,
  DIV_AniCat.NISP = data_AniCat.NISP$DIV_index,
  DIV_AniCat.MNI = data_AniCat.MNI$DIV_index,
  DIV_MNI.group = data_MNI.group$DIV_index,   
  DIV_NISP.group = data_NISP.group$DIV_index
)

# Join data with site and dating information
data_DIV.comb <- data_DIV.comb %>%
  left_join(data_sites, by = "Site")

#adding a list for later p-value mapping
my_comparisons <- list (
  c("EarlyNeo", "MiddleNeo"), 
  c("MiddleNeo", "LateNeo"), 
  c("LateNeo", "BronzeAge"), 
  c("EarlyNeo", "BronzeAge")
)
```

In the following, we prepare the data for the different plots and
summarize the taxa below a chosen threshold, so they can be summarized
in the plots. The taxa are calculated as relative values per time
frame.

### Function for data preparation

Here we create a function to be able to plot the relative taxa based
on NISP and MNI values.

### Figures

Here we apply the formula to create the plots for showing the
differences in relative species group per time slice. See Figure 3 in
main article.

```
p1 <- plot_relative_taxa(
  data = data_MNI,
  value_col = "MNI",
  phase_labels = phase_labels,
  custom_family_order = c( "Gadidae", "Pleuronectiformes", 
                           "Phocoidea","Bovidae", "Canidae", "Cervidae", 
                           "Suidae",  "Anatidae"),
  fill_colors = mlf_col,
  y_limit = 0.8,
  plot_title = "Relative MNI Histograms by Phase"
)

# Add vertical lines externally
separator_indices <- which(c("Gadidae", "Pleuronectiformes", 
                             "Phocoidea","Bovidae", "Canidae", "Cervidae", 
                             "Suidae",  "Anatidae") %in% c("Pleuronectiformes","Phocoidea",  "Suidae", "Anatidae")) + 0.5

p_relMNI <- p1 + geom_vline(xintercept = separator_indices,
                linetype = "solid",
                color = "black",
                linewidth = 0.5)


 p2 <- plot_relative_taxa(
  data = data_NISP,
  value_col = "NISP",
  phase_labels = phase_labels,
  custom_family_order = c("Anguillidae", "Pleuronectiformes",  
                          "Bovidae", "Canidae", "Cervidae", 
                          "Suidae"),
  fill_colors = mlf_col,
  y_limit = 0.8,
  plot_title = "Relative NISP Histograms by Phase"
)

# Add vertical lines externally
separator_indices <- which(c("Anguillidae", "Pleuronectiformes",  
                             "Bovidae", "Canidae", "Cervidae", 
                             "Suidae") %in% c("Pleuronectiformes","Suidae")) + 0.5

p_relNISP <-p2 + geom_vline(xintercept = separator_indices,
                linetype = "solid",
                color = "black",
                linewidth = 0.5)
```

Now we plot the data based on the diversity index of animal family
and animal class-based on NISP and MNI respectively.

```
#### DIVERSITY INDIEXES ###### 
# Plot diversity indices with dating information
p_divNISP <-  ggplot(data_DIV.comb, aes(DIV_AniCat.NISP, DIV_NISP.group, color = Dating_Info)) +
  geom_point()+
  labs(
    title ="Comparison of diversity indices (NISP)",
    x = "Diversity index (class)",
    y = "Diversity index (family)"
  )+
  geom_text(
    aes(label=Site),
    nudge_x = 0.005, nudge_y = -0.005,
    check_overlap = FALSE
  )+
  xlim(-0.1 , 0.9)+
  stat_smooth(method = "lm", aes(color=NULL), linetype = 0)+
  # add linear regression lines without standard deviation
  geom_line(stat="smooth", method = "lm", se = FALSE, alpha=0.25, linewidth=1.5)+  
  scale_color_manual(values = mlf_col)+
  theme_bw()+
  theme(legend.position = "bottom",
        legend.title = element_blank())

p_divMNI <-  ggplot(data_DIV.comb, aes(DIV_AniCat.MNI, DIV_MNI.group, color = Dating_Info)) +
  geom_point()+
  labs(
    title ="Comparison of diversity indices (MNI)",
    x = "Diversity index (class)",
    y = "Diversity index (family)"
  )+
  geom_text(
    aes(label=Site),
    nudge_x = 0.005, nudge_y = -0.005,
    check_overlap = FALSE
  )+
  xlim(-0.1 , 0.9)+
  stat_smooth(method = "lm", aes(color=NULL), linetype = 0)+
  # addf linear regression lines without standard deviation
  geom_line(stat="smooth", method = "lm", se = FALSE, alpha=0.25, linewidth=1.5)+  
  scale_color_manual(values = mlf_col)+
  theme_bw()+
  theme(legend.position = "bottom",
        legend.title = element_blank()) 

data_DIV.comb <- data_DIV.comb %>%
  left_join(cal_data, by ="Site") %>%
  mutate(Phase = factor(Dating_Info,
                        levels = c("Early Neolithic", "Middle Neolithic", "Late Neolithic", "Bronze Age")))
```

Finally we plot the diversity indexes chronologically and add a
regression curve.

```
p_divNISPchron <- ggplot(data_DIV.comb, aes(x = Median_CalBCAD, y = DIV_NISP.group)) +
  geom_point(size = 2, shape = 3) +
  #  geom_errorbarh(aes(xmin = Lower_2sigma, xmax = Upper_2sigma, height=0.01), alpha = 0.4) +
  labs(x = "Calendar year (BCE)", y = "Diversity Index (DI)") +
  theme_bw()+
  stat_smooth(method ="loess")+
  xlim(-4200,-950)

p_divMNIchron <-ggplot(data_DIV.comb, aes(x = Median_CalBCAD, y = DIV_MNI.group)) +
  geom_point(size = 2, shape = 3) +
  #  geom_errorbarh(aes(xmin = Lower_2sigma, xmax = Upper_2sigma, height=0.01), alpha = 0.4) +
  labs(x = "Calendar year (BCE)", y = "Diversity Index (DI)") +
  theme_bw()+
  stat_smooth(method ="loess")+
  xlim(-4200,-950)
```
