# Supplementary material for: Fishing for millennia: Effects and impacts of prehistoric fishing in the Syltholm Fjord, Denmark
Source: PLoS One. 2026 May 13;21(5):e0347863. doi: 10.1371/journal.pone.0347863 (PMC13170857; doi:10.1371/journal.pone.0347863)
Supplement: S2 File — (HTML) [file pone.0347863.s005.html]

Supplementary Information S2 File - Modelling prey choice in the prehistoric Syltholm Fjord


# Supplementary Information S2 File - Modelling prey choice in the prehistoric Syltholm Fjord

#### Sofie F. Hellerøe (https://orcid.org/0000-0001-9854-1312)

#### 2025

---

This R Markdown/HTML file (Supplementary Information S2 File) contains a
step-by-step guide to how we created the models (Fig. 5) in the paper
**Fishing for millennia: Effects and impacts of prehistoric
fishing in the Syltholm Fjord**.

---

The following section provides a concise overview of how we
implemented the Prey Choice Model (PCM) used in this study. It is
designed to accompany the main article and help readers understand how
the values used in our analyses were derived. This section is meant as a
transparent walkthrough of our modeling decisions, and while the
estimates are necessarily approximate, they are consistent and
comparable across species. Where applicable, we have drawn on published
datasets and previous implementations of the PCM to maintain
methodological consistency and allow for replication (see sections
below).

For further details on our interpretations and broader analytical
framework, please read the main article.

---

## The Prey Choice Model *in brief*

The basic principles of the PCM, which is based on the overarching
Optimal Foraging Theory framework, is explained in the main paper. We
therefore go straight to explaining where the values neccessary to
calculate PERR originate from.

### Calculating PERR

The simplest equation for calculating post encounter return rate is
this:

\[\text{Post encounter return rate (PERR)
=}\frac{e}{h} \times 60 \text{ (minutes)} \]

The post-encounter return rate (PERR) is thus defined as the energy
content of the prey (in kilocalories, *e*) divided by the
handling time (*h*, in minutes), with the resulting rate scaled
to a per-hour basis by multiplying by 60.

### *e* (kcals)

Caloric values expressed as kilocalories per 100 grams (kcal/100g)
were sourced from multiple references to ensure comprehensive coverage
across all species studied. For the majority of species, we utilized
kcal/100g values reported by Morin et al. (2022). To supplement these
data—particularly for avian, fish, and marine mammal species not
included in Morin et al., we incorporated nutritional information
compiled by Kuhnlein and Humphries (2017), which aggregates data from a
wide range of resources consumed by Indigenous peoples of North America.
Additionally, following the approach of Lupo and Schmitt (2016) in their
compilation of African fauna nutritional values (see their Table 3), we
consulted the publicly accessible database at http://www.nutritionvalue.org/ for select species
lacking data in the aforementioned sources.

In order to obtain *e*, the values found in the sources were
calculated by the average weight of each animal species, multiplied by
10 (to have the same unit) times kcal per 100g: \[e = \text{Weight (in kg)} \times 10 \times
\text{Kcal (per 100g)}\]

The sources used for obtaining *e* values for all species in
our dataset are linked directly in the csv file.

### *h* (post encounter handling time)

We mimic the approach and used some of the values for *h* in
the Supplementary Information from Yaworsky et al. 2023a, but without
incorporating pursuit failure. Yaworsky’s values were in part inferred
from Lupo & Schmitt (2016) but adjusted to European fauna instead of
the African species. Their step-by-step model explanation is available
open access via Zenodo (Yaworsky et al 2023b)

The models had issues estimating the handling times (*h*) for
the smaller game, as it is originally fitted to larger game. We
therefore specified the handling costs by inferring an estimate of the
amount of time each species would take to handle post encounter. It is
likely that these assumptions are not entirely accurate, but still
relatively precise, which is satisfactory enough to do the prey ranking
and understand trends in prey selection.

---

##### Literature mentioned above:

Kuhnlein, H. V., & Humphries, M. M. (2017). Traditional Animal
Foods of Indigenous Peoples of Northern North America: Http://traditionalanimalfoods.org/ [Centre for
Indigenous Peoples’ Nutrition and Environment, McGill University,
Montreal.].

Lupo, K. D., & Schmitt, D. N. (2016). When bigger is not better:
The economics of hunting megafauna and its implications for
Plio-Pleistocene hunter-gatherers. *Journal of Anthropological
Archaeology, 44*, 185–197. https://doi.org/10.1016/j.jaa.2016.07.012

Morin, E., Bird, D., Winterhalder, B., & Bliege Bird, R. (2022).
Deconstructing Hunting Returns: Can We Reconstruct and Predict Payoffs
from Pursuing Prey? *Journal of Archaeological Method and Theory,
29*, 561–623. https://doi.org/10.1007/s10816-021-09526-6

Yaworsky, P. M., Hussain, S. T., & Riede, F. (2023a).
Climate-driven habitat shifts of high-ranked prey species structure Late
Upper Paleolithic hunting. *Scientific Reports, 13*(1), 4238. https://doi.org/10.1038/s41598-023-31085-x

Yaworsky, P. M., Hussain, S. T., & Riede, F. (2023b).
Supplemental Information to Climate-driven habitat shifts of high-ranked
prey species structure Late Upper Paleolithic hunting (Version R version
4.2.1 (2022-06-23 ucrt) – ‘Funny-Looking Kid’ [Dataset]. Zenodo. https://doi.org/10.5281/ZENODO.7687831

---

## The two PCM graphs

To explore patterns in species representation, we applied two
complementary modelling approaches that relate species’ energetic
profitability (PERR) to their relative abundance in the assemblage
(NISP): one based on continuous values and one based on ordinal ranks.
These models are not intended to test a predictive relationship between
the two variables, but rather to provide a structured framework for
interpreting faunal exploitation in the context of persistent human
impact and subsistence practices.

The **PERR/PNISP model** retains the continuous values
of both post-encounter return rate (PERR) and proportional NISP. This
model highlights how differences in energetic return may correspond to
variation in species representation, and is useful for identifying
broader trends as well as potential outliers. It supports the
identification of species whose abundance aligns with or diverges from
what would be expected under an efficiency-driven exploitation
strategy.

The **PERR rank/NISP rank** model instead compares the
ordinal ranks of profitability and abundance. This approach does not
fully present the magnitude of differences and instead focuses on
overall consistency in ranking—asking whether species with higher PERR
values tend to be more represented, regardless of exact proportions. It
is less sensitive to outliers and better suited for identifying general
tendencies in prey selection patterns.

Together, these models offer a balanced view: the continuous model
provides granularity, while the rank-based model emphasizes robustness.
Both are used heuristically to evaluate whether patterns of resource use
reflect efficiency-based expectations, or if they suggest other
structuring factors such as ecological stress, cultural preferences, or
the influence of domestic species and managed landscapes.

### Statistical testing

While Pearson’s r (*p*-values) are reported for each model, it
is important to note that these values are provided for transparency
rather than as a central analytical focus. The primary purpose of the
models is not only to demonstrate a statistically significant
correlation between post-encounter return rates (PERR) and taxonomic
abundance (NISP), but to establish a structured baseline against which
patterns of faunal exploitation can be interpreted. In this context,
statistical significance is a secondary consideration, as the PCM is
used heuristically to explore potential deviations from efficiency-based
expectations and to situate resource use within broader environmental
and cultural dynamics.

---

## The Syltholm Fjord assemblages

The basic code chunks for creating the models shown in the paper are
shown for the first site on the list (Annasminde II) These are copied
for all the sites, which explains why they are not shown in the
remaining parts of the markdown. Code chunks appear throughout when they
are different from the standard code structure.

#### Load R packages

First, we load the R packages and set a few criteria for the
colouring of the model labels.

```
# Load required packages
packages <- c("ggplot2", "ggpubr", "ggpmisc", "ggrepel", "plotly", "stats", "RColorBrewer", "cowplot", "tidyverse", "forcats", "patchwork", "ggtrendline", "grid", "knitr", "kableExtra", "glue")

installed <- packages %in% rownames(installed.packages())
if (any(!installed)) install.packages(packages[!installed])

lapply(packages, library, character.only = TRUE)

# Define formulas
my.formula <- y ~ x
my.formulaexp <- y ~ exp(x)

# Define custom color palette

mlf_col_biome <- c(
  "Freshwater" = "#8FA595",     
  "Coastal" = "bisque",        
  "Mixed water" = "#A1B0C3",   
  "Mixed saltwater" = "azure2", 
  "Marine" = "#AFC9E8",         
  "Wetland" = "#B49A78",        
  "Forest" = "#7DAF9C",         
  "Mixed Land" = "#FFB24D",     
  "Land" = "#FFE799")
```

### The Dataset

Next we read the csv file with the information we need to create the
models. The dataset can be found in S1 Table.

```
# Load data
data <- read_csv2("S1_table.csv")


data %>%
 kable() %>%
  kable_styling(bootstrap_options = c("striped", "hover", "condensed", "responsive")) %>%
  scroll_box(width = "100%", height = "400px")
```

| Common\_name | Latin\_name | Family | Group\_Name | Animal\_Category | Class | Biome | DOM\_WILD | Size\_Class | Category | Site\_ID | Site\_name | Site\_type | Environment | Period | Period\_red | Min\_weight | Max\_weight | Avg\_weight | Kcals per 100 g | Kcals (e) | Kcal per 100 g or (e) source | Handling costs (h) | PERR | NISP | MNI | reference |
| --- | --- | --- | --- | --- | --- | --- | --- | --- | --- | --- | --- | --- | --- | --- | --- | --- | --- | --- | --- | --- | --- | --- | --- | --- | --- | --- |
| Atlantic Cod | Gadus morhua | Gadidae | Codfishes | Fish | Fish | Marine | wild | medium | small | Strandholm VI (MLF01232-I) | Strandholm VI | settlement | coastal | Bronze age | BA | 0.5 | 12.0 | 6.3 | 82 | 5125 | https://www.nutritionvalue.org/Fish%2C\_raw%2C\_Atlantic%2C\_cod\_nutritional\_value.html?size=100+g | 40 | 7688 | 60 | 31 | NA |
| Bovine | Bos spec | Bovidae | Cattle/Aurochs | Mammal | Ungulates | Mixed Land | indet | large | large | Syltholm II (MLF00906-III) | Syltholm II | settlement | coastal | Mesolithic-Neolithic | MesoNeo | 600.0 | 900.0 | 750.0 | 109 | 817500 | http://traditionalanimalfoods.org/mammals/hoofed/page.aspx?id=6136 (Kuhnlein & Humphries 2017) | 1600 | 30656 | 8 | 1 | NA |
| Brown Bear | Ursus arctos | Ursidae | Brown bears | Mammal | Carnivores | Mixed Land | wild | large | large | Syltholm II (MLF00906-III) | Syltholm II | settlement | coastal | Mesolithic-Neolithic | MesoNeo | 135.0 | 250.0 | 192.5 | 161 | 309925 | https://www.nutritionvalue.org/Game\_meat%2C\_raw%2C\_bear\_nutritional\_value.html?size=100+g & http://traditionalanimalfoods.org/mammals/bears/ (Kuhnlein & Humphries 2017) | 2100 | 8855 | 1 | 1 | NA |
| Buzzard | Buteo buteo & Pernis apivorus | Accipitridae | Buzzards | Bird | Raptors | Land | wild | medium | small | Syltholm II (MLF00906-II+III) | Syltholm II | settlement | coastal | Mesolithic-Neolithic | MesoNeo | 0.5 | 1.5 | 1.0 | 142 | 1420 | Morin et al 2022 | 30 | 2840 | 13 | 3 | NA |
| Carp | Cyprinidae | Cyprinidae | Carps | Fish | Fish | Freshwater | wild | medium | small | Gokartbane (MLF01333) | Gokartbane | settlement | coastal | Middle Neolithic | Neo | 2.0 | 5.0 | 3.5 | 127 | 4445 | https://www.nutritionvalue.org/Fish%2C\_raw%2C\_carp\_nutritional\_value.html?size=100+g | 25 | 10668 | 3 | indet | NA |
| Cattle | Bos taurus | Bovidae | Cattle | Mammal | Ungulates | Mixed Land | domestic | large | large | Annas Minde II (MLF01352) | Annasminde II | settlement | coastal | Neolithic-Bronze age | NeoBA | 400.0 | 800.0 | 600.0 | 109 | 654000 | http://traditionalanimalfoods.org/mammals/hoofed/page.aspx?id=6136 (Kuhnlein & Humphries 2017) | 2500 | 15696 | 141 | 4 | NA |
| Cattle | Bos taurus | Bovidae | Cattle | Mammal | Ungulates | Mixed Land | domestic | large | large | Annas Minde III (MLF01353) | Annasminde III | settlement | coastal | Neolithic-Bronze age | BA | 400.0 | 800.0 | 600.0 | 109 | 654000 | http://traditionalanimalfoods.org/mammals/hoofed/page.aspx?id=6136 (Kuhnlein & Humphries 2017) | 2500 | 15696 | 149 | 7 | NA |
| Cattle | Bos taurus | Bovidae | Cattle | Mammal | Ungulates | Mixed Land | domestic | large | large | Annasminde IV (MLF01354) | Annasminde IV | settlement | coastal | Neolithic-Bronze age | NeoBA | 400.0 | 800.0 | 600.0 | 109 | 654000 | http://traditionalanimalfoods.org/mammals/hoofed/page.aspx?id=6136 (Kuhnlein & Humphries 2017) | 2500 | 15696 | 282 | 4 | NA |
| Cattle | Bos taurus | Bovidae | Cattle | Mammal | Ungulates | Mixed Land | domestic | large | large | Annasminde V (MLF01355) | Annasminde V | settlement | coastal | Bronze age | BA | 400.0 | 800.0 | 600.0 | 109 | 654000 | http://traditionalanimalfoods.org/mammals/hoofed/page.aspx?id=6136 (Kuhnlein & Humphries 2017) | 2500 | 15696 | 101 | 6 | NA |
| Cattle | Bos taurus | Bovidae | Cattle | Mammal | Ungulates | Mixed Land | domestic | large | large | Finlandsvej II (MLF02548) | Finlandsvej II | settlement | coastal | Neolithic-Iron Age | NeoBA | 400.0 | 800.0 | 600.0 | 109 | 654000 | http://traditionalanimalfoods.org/mammals/hoofed/page.aspx?id=6136 (Kuhnlein & Humphries 2017) | 2500 | 15696 | 42 | 5 | NA |
| Cattle | Bos taurus | Bovidae | Cattle | Mammal | Ungulates | Mixed Land | domestic | large | large | Gokartbane (MLF01333) | Gokartbane | settlement | coastal | Middle Neolithic | Neo | 400.0 | 800.0 | 600.0 | 109 | 654000 | http://traditionalanimalfoods.org/mammals/hoofed/page.aspx?id=6136 (Kuhnlein & Humphries 2017) | 2500 | 15696 | 35 | 1 | NA |
| Cattle | Bos taurus | Bovidae | Cattle | Mammal | Ungulates | Mixed Land | domestic | large | large | RGS90 (MLF00952) | RGS90 | settlement | coastal | Neolithic-Bronze age | BA | 400.0 | 800.0 | 600.0 | 109 | 654000 | http://traditionalanimalfoods.org/mammals/hoofed/page.aspx?id=6136 (Kuhnlein & Humphries 2017) | 2500 | 15696 | 36 | 3 | Bangsgaard 2016 |
| Cattle | Bos taurus | Bovidae | Cattle | Mammal | Ungulates | Mixed Land | domestic | large | large | Strandholm I (MLF00909-I+II) | Strandholm I | settlement | coastal | Neolithic | Neo | 400.0 | 800.0 | 600.0 | 109 | 654000 | http://traditionalanimalfoods.org/mammals/hoofed/page.aspx?id=6136 (Kuhnlein & Humphries 2017) | 2500 | 15696 | 34 | 4 | NA |
| Cattle | Bos taurus | Bovidae | Cattle | Mammal | Ungulates | Mixed Land | domestic | large | large | Strandholm V (MLF01182) | Strandholm V | settlement | coastal | Neolithic-Bronze age | Neo | 400.0 | 800.0 | 600.0 | 109 | 654000 | http://traditionalanimalfoods.org/mammals/hoofed/page.aspx?id=6136 (Kuhnlein & Humphries 2017) | 2500 | 15696 | 7 | 1 | NA |
| Cattle | Bos taurus | Bovidae | Cattle | Mammal | Ungulates | Mixed Land | domestic | large | large | Strandholm VI (MLF01232-I) | Strandholm VI | settlement | coastal | Bronze age | BA | 400.0 | 800.0 | 600.0 | 109 | 654000 | http://traditionalanimalfoods.org/mammals/hoofed/page.aspx?id=6136 (Kuhnlein & Humphries 2017) | 2500 | 15696 | 13 | 2 | NA |
| Cattle | Bos taurus | Bovidae | Cattle | Mammal | Ungulates | Mixed Land | domestic | large | large | Syltholm II (MLF00906-I+II+III) | Syltholm II | settlement | coastal | Mesolithic-Neolithic | MesoNeo | 400.0 | 800.0 | 600.0 | 109 | 654000 | http://traditionalanimalfoods.org/mammals/hoofed/page.aspx?id=6136 (Kuhnlein & Humphries 2017) | 2500 | 15696 | 120 | 7 | Analysis + MNI: Pernille Bangsgaard |
| Cattle | Bos taurus | Bovidae | Cattle | Mammal | Ungulates | Mixed Land | domestic | large | large | Syltholm IX (MLF00935-I+II+III) | Syltholm IX | settlement | coastal | Early Neolithic-Early Bronze Age | Neo | 400.0 | 800.0 | 600.0 | 109 | 654000 | http://traditionalanimalfoods.org/mammals/hoofed/page.aspx?id=6136 (Kuhnlein & Humphries 2017) | 2500 | 15696 | 128 | 9 | NA |
| Cattle | Bos taurus | Bovidae | Cattle | Mammal | Ungulates | Mixed Land | domestic | large | large | Syltholm V (MLF00910) | Syltholm V | settlement | coastal | Late Mesolithic-Neolithic | MesoNeo | 400.0 | 800.0 | 600.0 | 109 | 654000 | http://traditionalanimalfoods.org/mammals/hoofed/page.aspx?id=6136 (Kuhnlein & Humphries 2017) | 2500 | 15696 | 2 | 1 | NA |
| Cattle | Bos taurus | Bovidae | Cattle | Mammal | Ungulates | Mixed Land | domestic | large | large | Syltholm VII (MLF00933-III) | Syltholm VII | settlement | coastal | Late Palaeolithic-middle Neolithic | Neo | 400.0 | 800.0 | 600.0 | 109 | 654000 | http://traditionalanimalfoods.org/mammals/hoofed/page.aspx?id=6136 (Kuhnlein & Humphries 2017) | 2500 | 15696 | 1 | 1 | NA |
| Cattle | Bos taurus | Bovidae | Cattle | Mammal | Ungulates | Mixed Land | domestic | large | large | Syltholm X (MLF00936) | Syltholm X | settlement | coastal | Late Mesolithic-early Neolithic | MesoNeo | 400.0 | 800.0 | 600.0 | 109 | 654000 | http://traditionalanimalfoods.org/mammals/hoofed/page.aspx?id=6136 (Kuhnlein & Humphries 2017) | 2500 | 15696 | 23 | 1 | NA |
| Codfish | Gadidae | Gadidae | Codfishes | Fish | Fish | Marine | wild | medium | small | Annasminde IV (MLF01354) | Annasminde IV | settlement | coastal | Neolithic-Bronze age | NeoBA | 0.5 | 12.0 | 6.3 | 82 | 5125 | https://www.nutritionvalue.org/Fish%2C\_raw%2C\_Atlantic%2C\_cod\_nutritional\_value.html?size=100+g | 40 | 7688 | 1 | 1 | NA |
| Codfish | Gadidae | Gadidae | Codfishes | Fish | Fish | Marine | wild | medium | small | Strandholm VI (MLF01232-I) | Strandholm VI | settlement | coastal | Bronze age | BA | 0.5 | 12.0 | 6.3 | 82 | 5125 | https://www.nutritionvalue.org/Fish%2C\_raw%2C\_Atlantic%2C\_cod\_nutritional\_value.html?size=100+g | 40 | 7688 | 22 | indet | NA |
| Codfish | Gadidae | Gadidae | Codfishes | Fish | Fish | Marine | wild | medium | small | Syltholm II (MLF00906-II+III) | Syltholm II | settlement | coastal | Mesolithic-Neolithic | MesoNeo | 0.5 | 12.0 | 6.3 | 82 | 5125 | https://www.nutritionvalue.org/Fish%2C\_raw%2C\_Atlantic%2C\_cod\_nutritional\_value.html?size=100+g | 40 | 7688 | 6 | NA | NA |
| Codfish | Gadidae | Gadidae | Codfishes | Fish | Fish | Marine | wild | medium | small | Syltholm IX (MLF00935-II) | Syltholm IX | settlement | coastal | Early Neolithic-Early Bronze Age | Neo | 0.5 | 12.0 | 6.3 | 82 | 5125 | https://www.nutritionvalue.org/Fish%2C\_raw%2C\_Atlantic%2C\_cod\_nutritional\_value.html?size=100+g | 40 | 7688 | 1 | indet | NA |
| Codfish | Gadidae | Gadidae | Codfishes | Fish | Fish | Marine | wild | medium | small | Syltholm X (MLF00936) | Syltholm X | settlement | coastal | Late Mesolithic-early Neolithic | MesoNeo | 0.5 | 12.0 | 6.3 | 82 | 5125 | https://www.nutritionvalue.org/Fish%2C\_raw%2C\_Atlantic%2C\_cod\_nutritional\_value.html?size=100+g | 40 | 7688 | 3 | indet | NA |
| Codfish | Gadidae | Gadidae | Codfishes | Fish | Fish | Marine | wild | medium | small | Syltholm XIV (MLF00940) | Syltholm XIV | settlement | coastal | Early Mesolithic-Late Mesolithic | Meso | 0.5 | 12.0 | 6.3 | 82 | 5125 | https://www.nutritionvalue.org/Fish%2C\_raw%2C\_Atlantic%2C\_cod\_nutritional\_value.html?size=100+g | 40 | 7688 | 2 | 2 | NA |
| Common Buzzard | Buteo buteo | Accipitridae | Buzzards | Bird | Raptors | Land | wild | medium | small | Syltholm X (MLF00936) | Syltholm X | settlement | coastal | Late Mesolithic-early Neolithic | MesoNeo | 0.5 | 1.5 | 1.0 | 142 | 1420 | Morin et al 2022 | 30 | 2840 | 1 | 1 | NA |
| Common Eider | Somateria mollissima | Anatidae | Eiders | Bird | Waterfowl | Coastal | wild | medium | small | Strandholm I (MLF00909-II) | Strandholm I | settlement | coastal | Neolithic | Neo | 1.0 | 3.0 | 2.0 | 121 | 2420 | Morin et al 2022 | 90 | 1613 | 7 | 2 | NA |
| Common Wood Pigeon | Columba palumbus | Columbidae | Pigeons | Bird | Landbirds | Forest | wild | small | small | Syltholm II (MLF00906-II) | Syltholm II | settlement | coastal | Mesolithic-Neolithic | MesoNeo | 0.3 | 0.6 | 0.5 | 142 | 639 | Morin et al 2022 | 15 | 2556 | 1 | 1 | NA |
| Crab | Brachyura sp. | Decapoda | Crabs | Crustacean | Crustaceans | Coastal | wild | small | small | Syltholm XIII (MLF00939-I) | Syltholm XIII | settlement | coastal | Late Mesolithic-early Neolithic | MesoNeo | 0.1 | 0.1 | 0.1 | 57 | 40 | http://traditionalanimalfoods.org/marine-invertebrates/crustaceans/ (Kuhnlein & Humphries 2017) | 10 | 239 | 1 | 1 | NA |
| Dabbling Duck | Anas sp. | Anatidae | Ducks | Bird | Waterfowl | Wetland | wild | small | small | Annas Minde II (MLF01352) | Annasminde II | settlement | coastal | Neolithic-Bronze age | NeoBA | 0.5 | 1.5 | 1.0 | 227 | 2270 | http://traditionalanimalfoods.org/birds/waterfowl/page.aspx?id=6456 (Kuhnlein & Humphries 2017) | 30 | 4540 | 3 | indet | NA |
| Dabbling Duck | Anas sp. | Anatidae | Ducks | Bird | Waterfowl | Wetland | wild | small | small | Annasminde IV (MLF01354) | Annasminde IV | settlement | coastal | Neolithic-Bronze age | NeoBA | 0.5 | 1.5 | 1.0 | 227 | 2270 | http://traditionalanimalfoods.org/birds/waterfowl/page.aspx?id=6456 (Kuhnlein & Humphries 2017) | 30 | 4540 | 1 | 1 | NA |
| Dabbling Duck | Anas sp. | Anatidae | Ducks | Bird | Waterfowl | Wetland | wild | small | small | Strandholm I (MLF00909-II) | Strandholm I | settlement | coastal | Neolithic | Neo | 0.5 | 1.5 | 1.0 | 227 | 2270 | http://traditionalanimalfoods.org/birds/waterfowl/page.aspx?id=6456 (Kuhnlein & Humphries 2017) | 30 | 4540 | 6 | 2 | NA |
| Dabbling Duck | Anas sp. | Anatidae | Ducks | Bird | Waterfowl | Wetland | wild | small | small | Syltholm II (MLF00906-III) | Syltholm II | settlement | coastal | Mesolithic-Neolithic | MesoNeo | 0.5 | 1.5 | 1.0 | 227 | 2270 | http://traditionalanimalfoods.org/birds/waterfowl/page.aspx?id=6456 (Kuhnlein & Humphries 2017) | 30 | 4540 | 92 | 14 | Analysis: Pernille Bangsgaard |
| Dabbling Duck | Anas sp. | Anatidae | Ducks | Bird | Waterfowl | Wetland | wild | small | small | Syltholm X (MLF00936) | Syltholm X | settlement | coastal | Late Mesolithic-early Neolithic | MesoNeo | 0.5 | 1.5 | 1.0 | 227 | 2270 | http://traditionalanimalfoods.org/birds/waterfowl/page.aspx?id=6456 (Kuhnlein & Humphries 2017) | 30 | 4540 | 15 | 3 | NA |
| Dabbling Duck | Anas sp. | Anatidae | Ducks | Bird | Waterfowl | Wetland | wild | small | small | Syltholm XIII (MLF00939-I+II) | Syltholm XIII | settlement | coastal | Late Mesolithic-early Neolithic | MesoNeo | 0.5 | 1.5 | 1.0 | 227 | 2270 | http://traditionalanimalfoods.org/birds/waterfowl/page.aspx?id=6456 (Kuhnlein & Humphries 2017) | 30 | 4540 | 22 | 4 | NA |
| Dabbling Duck | Anas sp. | Anatidae | Ducks | Bird | Waterfowl | Wetland | wild | small | small | Syltholm XIV (MLF00940) | Syltholm XIV | settlement | coastal | Early Mesolithic-Late Mesolithic | Meso | 0.5 | 1.5 | 1.0 | 227 | 2270 | http://traditionalanimalfoods.org/birds/waterfowl/page.aspx?id=6456 (Kuhnlein & Humphries 2017) | 30 | 4540 | 2 | 2 | NA |
| Dog | Canis familiaris | Canidae | Dogs | Mammal | Carnivores | Mixed Land | domestic | medium | small | Annas Minde II (MLF01352) | Annasminde II | settlement | coastal | Neolithic-Bronze age | NeoBA | 10.0 | 30.0 | 20.0 | 138 | 27600 | Morin et al 2022 & http://traditionalanimalfoods.org/mammals/domesticated/#dog-e (Kuhnlein & Humphries 2017) | 450 | 3680 | 2 | 1 | NA |
| Dog | Canis familiaris | Canidae | Dogs | Mammal | Carnivores | Mixed Land | domestic | medium | small | Annas Minde III (MLF01353) | Annasminde III | settlement | coastal | Neolithic-Bronze age | BA | 10.0 | 30.0 | 20.0 | 138 | 27600 | Morin et al 2022 & http://traditionalanimalfoods.org/mammals/domesticated/#dog-e (Kuhnlein & Humphries 2017) | 450 | 3680 | 17 | 1 | NA |
| Dog | Canis familiaris | Canidae | Dogs | Mammal | Carnivores | Mixed Land | domestic | medium | small | Annasminde IV (MLF01354) | Annasminde IV | settlement | coastal | Neolithic-Bronze age | NeoBA | 10.0 | 30.0 | 20.0 | 138 | 27600 | Morin et al 2022 & http://traditionalanimalfoods.org/mammals/domesticated/#dog-e (Kuhnlein & Humphries 2017) | 450 | 3680 | 31 | 2 | NA |
| Dog | Canis familiaris | Canidae | Dogs | Mammal | Carnivores | Mixed Land | domestic | medium | small | Annasminde V (MLF01355) | Annasminde V | settlement | coastal | Bronze age | BA | 10.0 | 30.0 | 20.0 | 138 | 27600 | Morin et al 2022 & http://traditionalanimalfoods.org/mammals/domesticated/#dog-e (Kuhnlein & Humphries 2017) | 450 | 3680 | 7 | 1 | NA |
| Dog | Canis familiaris | Canidae | Dogs | Mammal | Carnivores | Mixed Land | domestic | medium | small | Finlandsvej II (MLF02548) | Finlandsvej II | settlement | coastal | Neolithic-Iron Age | NeoBA | 10.0 | 30.0 | 20.0 | 138 | 27600 | Morin et al 2022 & http://traditionalanimalfoods.org/mammals/domesticated/#dog-e (Kuhnlein & Humphries 2017) | 450 | 3680 | 2 | 1 | NA |
| Dog | Canis familiaris | Canidae | Dogs | Mammal | Carnivores | Mixed Land | domestic | medium | small | RGS90 (MLF00952) | RGS90 | settlement | coastal | Neolithic-Bronze age | BA | 10.0 | 30.0 | 20.0 | 138 | 27600 | Morin et al 2022 & http://traditionalanimalfoods.org/mammals/domesticated/#dog-e (Kuhnlein & Humphries 2017) | 450 | 3680 | 1 | 1 | Bangsgaard 2016 |
| Dog | Canis familiaris | Canidae | Dogs | Mammal | Carnivores | Mixed Land | domestic | medium | small | Strandholm I (MLF00909-I+II) | Strandholm I | settlement | coastal | Neolithic | Neo | 10.0 | 30.0 | 20.0 | 138 | 27600 | Morin et al 2022 & http://traditionalanimalfoods.org/mammals/domesticated/#dog-e (Kuhnlein & Humphries 2017) | 450 | 3680 | 91 | 2 | NA |
| Dog | Canis familiaris | Canidae | Dogs | Mammal | Carnivores | Mixed Land | domestic | medium | small | Syltholm II (MLF00906-I+II+III) | Syltholm II | settlement | coastal | Mesolithic-Neolithic | MesoNeo | 10.0 | 30.0 | 20.0 | 138 | 27600 | Morin et al 2022 & http://traditionalanimalfoods.org/mammals/domesticated/#dog-e (Kuhnlein & Humphries 2017) | 450 | 3680 | 326 | 13 | Analysis + MNI: Pernille Bangsgaard |
| Dog Genus | Canis sp. | Canidae | Dogs/Foxes/Wolves | Mammal | Carnivores | Mixed Land | indet | medium | small | Strandholm V (MLF01182) | Strandholm V | settlement | coastal | Neolithic-Bronze age | Neo | 10.0 | 30.0 | 20.0 | 138 | 27600 | Morin et al 2022 & http://traditionalanimalfoods.org/mammals/domesticated/#dog-e (Kuhnlein & Humphries 2017) | 450 | 3680 | 1 | 1 | NA |
| Dog Genus | Canis sp. | Canidae | Dogs/Foxes/Wolves | Mammal | Carnivores | Mixed Land | indet | medium | small | Syltholm II (MLF00906-I+II+III) | Syltholm II | settlement | coastal | Mesolithic-Neolithic | MesoNeo | 10.0 | 30.0 | 20.0 | 138 | 27600 | Morin et al 2022 & http://traditionalanimalfoods.org/mammals/domesticated/#dog-e (Kuhnlein & Humphries 2017) | 450 | 3680 | 29 | 3 | Analysis: Pernille Bangsgaard |
| Domestic Cat | Felis catus | Felidae | Cats | Mammal | Carnivores | Land | domestic | small | small | Annasminde IV (MLF01354) | Annasminde IV | settlement | coastal | Neolithic-Bronze age | NeoBA | 3.0 | 6.0 | 4.5 | 115 | 5175 | Morin et al 2022 | 200 | 1553 | 1 | 1 | NA |
| Domestic Goat | Capra hircus | Bovidae | Domestic goats | Mammal | Ungulates | Mixed Land | domestic | medium | medium | Annas Minde III (MLF01353) | Annasminde III | settlement | coastal | Neolithic-Bronze age | BA | 45.0 | 100.0 | 72.5 | 109 | 79025 | https://www.nutritionvalue.org/Game\_meat%2C\_raw%2C\_goat\_nutritional\_value.html?size=100+g | 700 | 6774 | 2 | 1 | NA |
| Domestic Goat | Capra hircus | Bovidae | Domestic goats | Mammal | Ungulates | Mixed Land | domestic | medium | medium | Annasminde IV (MLF01354) | Annasminde IV | settlement | coastal | Neolithic-Bronze age | NeoBA | 45.0 | 100.0 | 72.5 | 109 | 79025 | https://www.nutritionvalue.org/Game\_meat%2C\_raw%2C\_goat\_nutritional\_value.html?size=100+g | 700 | 6774 | 3 | 2 | NA |
| Domestic Goat | Capra hircus | Bovidae | Domestic goats | Mammal | Ungulates | Mixed Land | domestic | medium | medium | Annasminde V (MLF01355) | Annasminde V | settlement | coastal | Bronze age | BA | 45.0 | 100.0 | 72.5 | 109 | 79025 | https://www.nutritionvalue.org/Game\_meat%2C\_raw%2C\_goat\_nutritional\_value.html?size=100+g | 700 | 6774 | 5 | 1 | NA |
| Domestic Goat | Capra hircus | Bovidae | Domestic goats | Mammal | Ungulates | Mixed Land | domestic | medium | medium | Finlandsvej II (MLF02548) | Finlandsvej II | settlement | coastal | Neolithic-Iron Age | NeoBA | 45.0 | 100.0 | 72.5 | 109 | 79025 | https://www.nutritionvalue.org/Game\_meat%2C\_raw%2C\_goat\_nutritional\_value.html?size=100+g | 700 | 6774 | 2 | 1 | NA |
| Domestic Goat | Capra hircus | Bovidae | Domestic goats | Mammal | Ungulates | Mixed Land | domestic | medium | medium | Strandholm I (MLF00909-II) | Strandholm I | settlement | coastal | Neolithic | Neo | 45.0 | 100.0 | 72.5 | 109 | 79025 | https://www.nutritionvalue.org/Game\_meat%2C\_raw%2C\_goat\_nutritional\_value.html?size=100+g | 700 | 6774 | 2 | 1 | NA |
| Domestic Goat | Capra hircus | Bovidae | Domestic goats | Mammal | Ungulates | Mixed Land | domestic | medium | medium | Syltholm II (MLF00906-II) | Syltholm II | settlement | coastal | Mesolithic-Neolithic | MesoNeo | 45.0 | 100.0 | 72.5 | 109 | 79025 | https://www.nutritionvalue.org/Game\_meat%2C\_raw%2C\_goat\_nutritional\_value.html?size=100+g | 700 | 6774 | 2 | 1 | NA |
| Domestic Pig | Sus domesticus | Suidae | Domestic pigs | Mammal | Ungulates | Mixed Land | domestic | medium | large | Annas Minde III (MLF01353) | Annasminde III | settlement | coastal | Neolithic-Bronze age | BA | 50.0 | 200.0 | 125.0 | 122 | 152500 | https://www.nutritionvalue.org/Game\_meat%2C\_raw%2C\_wild%2C\_boar\_nutritional\_value.html?size=100+g | 900 | 10167 | 81 | 2 | NA |
| Domestic Pig | Sus domesticus | Suidae | Domestic pigs | Mammal | Ungulates | Mixed Land | domestic | medium | large | Annasminde IV (MLF01354) | Annasminde IV | settlement | coastal | Neolithic-Bronze age | NeoBA | 50.0 | 200.0 | 125.0 | 122 | 152500 | https://www.nutritionvalue.org/Game\_meat%2C\_raw%2C\_wild%2C\_boar\_nutritional\_value.html?size=100+g | 900 | 10167 | 6 | 1 | NA |
| Domestic Pig | Sus domesticus | Suidae | Domestic pigs | Mammal | Ungulates | Mixed Land | domestic | medium | large | Strandholm I (MLF00909-II) | Strandholm I | settlement | coastal | Neolithic | Neo | 50.0 | 200.0 | 125.0 | 122 | 152500 | https://www.nutritionvalue.org/Game\_meat%2C\_raw%2C\_wild%2C\_boar\_nutritional\_value.html?size=100+g | 900 | 10167 | 1 | 1 | NA |
| Domestic Pig | Sus domesticus | Suidae | Domestic pigs | Mammal | Ungulates | Mixed Land | domestic | medium | large | Syltholm II (MLF00906-I+III) | Syltholm II | settlement | coastal | Mesolithic-Neolithic | MesoNeo | 50.0 | 200.0 | 125.0 | 122 | 152500 | https://www.nutritionvalue.org/Game\_meat%2C\_raw%2C\_wild%2C\_boar\_nutritional\_value.html?size=100+g | 900 | 10167 | 4 | 2 | Analysis: Pernille Bangsgaard |
| Domestic Sheep | Ovis aries | Bovidae | Domestic sheep | Mammal | Ungulates | Mixed Land | domestic | medium | medium | Annas Minde II (MLF01352) | Annasminde II | settlement | coastal | Neolithic-Bronze age | NeoBA | 45.0 | 100.0 | 72.5 | 109 | 79025 | https://www.nutritionvalue.org/Game\_meat%2C\_raw%2C\_goat\_nutritional\_value.html?size=100+g | 700 | 6774 | 16 | 3 | NA |
| Domestic Sheep | Ovis aries | Bovidae | Domestic sheep | Mammal | Ungulates | Mixed Land | domestic | medium | medium | Annas Minde III (MLF01353) | Annasminde III | settlement | coastal | Neolithic-Bronze age | BA | 45.0 | 100.0 | 72.5 | 109 | 79025 | https://www.nutritionvalue.org/Game\_meat%2C\_raw%2C\_goat\_nutritional\_value.html?size=100+g | 700 | 6774 | 5 | 1 | NA |
| Domestic Sheep | Ovis aries | Bovidae | Domestic sheep | Mammal | Ungulates | Mixed Land | domestic | medium | medium | Annasminde IV (MLF01354) | Annasminde IV | settlement | coastal | Neolithic-Bronze age | NeoBA | 45.0 | 100.0 | 72.5 | 109 | 79025 | https://www.nutritionvalue.org/Game\_meat%2C\_raw%2C\_goat\_nutritional\_value.html?size=100+g | 700 | 6774 | 7 | 1 | NA |
| Domestic Sheep | Ovis aries | Bovidae | Domestic sheep | Mammal | Ungulates | Mixed Land | domestic | medium | medium | Annasminde V (MLF01355) | Annasminde V | settlement | coastal | Bronze age | BA | 45.0 | 100.0 | 72.5 | 109 | 79025 | https://www.nutritionvalue.org/Game\_meat%2C\_raw%2C\_goat\_nutritional\_value.html?size=100+g | 700 | 6774 | 5 | 1 | NA |
| Domestic Sheep | Ovis aries | Bovidae | Domestic sheep | Mammal | Ungulates | Mixed Land | domestic | medium | medium | RGS90 (MLF00952) | RGS90 | settlement | coastal | Neolithic-Bronze age | BA | 45.0 | 100.0 | 72.5 | 109 | 79025 | https://www.nutritionvalue.org/Game\_meat%2C\_raw%2C\_goat\_nutritional\_value.html?size=100+g | 700 | 6774 | 4 | 1 | Bangsgaard 2016 |
| Domestic Sheep | Ovis aries | Bovidae | Domestic sheep | Mammal | Ungulates | Mixed Land | domestic | medium | medium | Strandholm I (MLF00909-I+II) | Strandholm I | settlement | coastal | Neolithic | Neo | 45.0 | 100.0 | 72.5 | 109 | 79025 | https://www.nutritionvalue.org/Game\_meat%2C\_raw%2C\_goat\_nutritional\_value.html?size=100+g | 700 | 6774 | 5 | 2 | NA |
| Domestic Sheep | Ovis aries | Bovidae | Domestic sheep | Mammal | Ungulates | Mixed Land | domestic | medium | medium | Syltholm II (MLF00906-I+II+III) | Syltholm II | settlement | coastal | Mesolithic-Neolithic | MesoNeo | 45.0 | 100.0 | 72.5 | 109 | 79025 | https://www.nutritionvalue.org/Game\_meat%2C\_raw%2C\_goat\_nutritional\_value.html?size=100+g | 700 | 6774 | 9 | 3 | NA |
| Domestic Sheep | Ovis aries | Bovidae | Domestic sheep | Mammal | Ungulates | Mixed Land | domestic | medium | medium | Syltholm IX (MLF00935-II+III) | Syltholm IX | settlement | coastal | Early Neolithic-Early Bronze Age | Neo | 45.0 | 100.0 | 72.5 | 109 | 79025 | https://www.nutritionvalue.org/Game\_meat%2C\_raw%2C\_goat\_nutritional\_value.html?size=100+g | 700 | 6774 | 12 | 3 | NA |
| Domestic Sheep | Ovis aries | Bovidae | Domestic sheep | Mammal | Ungulates | Mixed Land | domestic | medium | medium | Syltholm XIII (MLF00939-I) | Syltholm XIII | settlement | coastal | Late Mesolithic-early Neolithic | MesoNeo | 45.0 | 100.0 | 72.5 | 109 | 79025 | https://www.nutritionvalue.org/Game\_meat%2C\_raw%2C\_goat\_nutritional\_value.html?size=100+g | 700 | 6774 | 1 | 1 | NA |
| Duck | Anatini/aythya/somateria | Anatidae | Ducks | Bird | Waterfowl | Wetland | wild | small | small | Syltholm IX (MLF00935-I+II+III) | Syltholm IX | settlement | coastal | Early Neolithic-Early Bronze Age | Neo | 0.5 | 1.5 | 1.0 | 227 | 2270 | http://traditionalanimalfoods.org/birds/waterfowl/page.aspx?id=6456 (Kuhnlein & Humphries 2017) | 30 | 4540 | 6 | 5 | NA |
| Duck | Anas acceca | Anatidae | Ducks | Bird | Waterfowl | Wetland | wild | small | small | Syltholm XIII (MLF00939-I) | Syltholm XIII | settlement | coastal | Late Mesolithic-early Neolithic | MesoNeo | 0.5 | 1.5 | 1.0 | 227 | 2270 | http://traditionalanimalfoods.org/birds/waterfowl/page.aspx?id=6456 (Kuhnlein & Humphries 2017) | 30 | 4540 | 1 | 1 | NA |
| Eagle | Haliaeetus / Aquila | Accipitridae | Eagles | Bird | Raptors | Forest | wild | large | small | Syltholm II (MLF00906-I) | Syltholm II | settlement | coastal | Mesolithic-Neolithic | MesoNeo | 2.6 | 7.0 | 4.8 | 142 | 6816 | Morin et al 2022 & http://traditionalanimalfoods.org/birds/birds-of-prey/ (Kuhnlein & Humphries 2017) | 110 | 3718 | 1 | 1 | Analysis: Pernille Bangsgaard |
| Earless Seal | Phocidea | Phocoidea | Seals | Marine Mammal | True seals | Marine | wild | medium | medium | Strandholm I (MLF00909-I) | Strandholm I | settlement | coastal | Neolithic | Neo | 55.0 | 170.0 | 112.5 | 150 | 168750 | An approximate number based on multiple species and body parts in raw form from http://traditionalanimalfoods.org/mammals/seals-sealions-walrus/page.aspx?id=6392#harp-e (Kuhnlein & Humphries 2017) | 900 | 11250 | 1 | 1 | NA |
| Earless Seal | Phocidea | Phocoidea | Seals | Marine Mammal | True seals | Marine | wild | medium | medium | Syltholm X (MLF00936) | Syltholm X | settlement | coastal | Late Mesolithic-early Neolithic | MesoNeo | 55.0 | 170.0 | 112.5 | 150 | 168750 | An approximate number based on multiple species and body parts in raw form from http://traditionalanimalfoods.org/mammals/seals-sealions-walrus/page.aspx?id=6392#harp-e (Kuhnlein & Humphries 2017) | 900 | 11250 | 18 | 2 | NA |
| Eider | Somateria sp. | Anatidae | Eiders | Bird | Waterfowl | Coastal | wild | medium | small | Syltholm II (MLF00906-III) | Syltholm II | settlement | coastal | Mesolithic-Neolithic | MesoNeo | 1.0 | 3.0 | 2.0 | 121 | 2420 | Morin et al 2022 | 90 | 1613 | 3 | 2 | NA |
| Eurasian Otter | Lutra lutra | Mustelidae | Otters | Mammal | Carnivores | Wetland | wild | small | small | Syltholm II (MLF00906-I+II+III) | Syltholm II | settlement | coastal | Mesolithic-Neolithic | MesoNeo | 7.0 | 12.0 | 9.5 | 130 | 12350 | Used badger value from Morin et al 2022 | 300 | 2470 | 111 | 8 | Analysis + MNI: Pernille Bangsgaard |
| Eurasian Otter | Lutra lutra | Mustelidae | Otters | Mammal | Carnivores | Wetland | wild | small | small | Syltholm V (MLF00910) | Syltholm V | settlement | coastal | Late Mesolithic-Neolithic | MesoNeo | 7.0 | 12.0 | 9.5 | 130 | 12350 | Used badger value from Morin et al 2022 | 300 | 2470 | 1 | 1 | NA |
| European Eel | Anguilla anguilla | Anguillidae | Eels | Fish | Fish | Mixed water | wild | medium | small | Annas Minde III (MLF01353) | Annasminde III | settlement | coastal | Neolithic-Bronze age | BA | 0.5 | 3.0 | 1.8 | 184 | 3220 | https://www.nutritionvalue.org/Fish%2C\_raw%2C\_mixed\_species%2C\_eel\_nutritional\_value.html?size=100+g | 20 | 9660 | 3 | indet | NA |
| European Eel | Anguilla anguilla | Anguillidae | Eels | Fish | Fish | Mixed water | wild | medium | small | Annasminde IV (MLF01354) | Annasminde IV | settlement | coastal | Neolithic-Bronze age | NeoBA | 0.5 | 3.0 | 1.8 | 184 | 3220 | https://www.nutritionvalue.org/Fish%2C\_raw%2C\_mixed\_species%2C\_eel\_nutritional\_value.html?size=100+g | 20 | 9660 | 662 | indet | NA |
| European Eel | Anguilla anguilla | Anguillidae | Eels | Fish | Fish | Mixed water | wild | medium | small | Gokartbane (MLF01333) | Gokartbane | settlement | coastal | Middle Neolithic | Neo | 0.5 | 3.0 | 1.8 | 184 | 3220 | https://www.nutritionvalue.org/Fish%2C\_raw%2C\_mixed\_species%2C\_eel\_nutritional\_value.html?size=100+g | 20 | 9660 | 606 | indet | NA |
| European Eel | Anguilla anguilla | Anguillidae | Eels | Fish | Fish | Mixed water | wild | medium | small | Strandholm VI (MLF01232-I) | Strandholm VI | settlement | coastal | Bronze age | BA | 0.5 | 3.0 | 1.8 | 184 | 3220 | https://www.nutritionvalue.org/Fish%2C\_raw%2C\_mixed\_species%2C\_eel\_nutritional\_value.html?size=100+g | 20 | 9660 | 57 | 2 | NA |
| European Eel | Anguilla anguilla | Anguillidae | Eels | Fish | Fish | Mixed water | wild | medium | small | Syltholm II (MLF00906-II+III) | Syltholm II | settlement | coastal | Mesolithic-Neolithic | MesoNeo | 0.5 | 3.0 | 1.8 | 184 | 3220 | https://www.nutritionvalue.org/Fish%2C\_raw%2C\_mixed\_species%2C\_eel\_nutritional\_value.html?size=100+g | 20 | 9660 | 105 | NA | NA |
| European Eel | Anguilla anguilla | Anguillidae | Eels | Fish | Fish | Mixed water | wild | medium | small | Syltholm IX (MLF00935-II) | Syltholm IX | settlement | coastal | Early Neolithic-Early Bronze Age | Neo | 0.5 | 3.0 | 1.8 | 184 | 3220 | https://www.nutritionvalue.org/Fish%2C\_raw%2C\_mixed\_species%2C\_eel\_nutritional\_value.html?size=100+g | 20 | 9660 | 1 | indet | NA |
| European Eel | Anguilla anguilla | Anguillidae | Eels | Fish | Fish | Mixed water | wild | medium | small | Syltholm V (MLF00910) | Syltholm V | settlement | coastal | Late Mesolithic-Neolithic | MesoNeo | 0.5 | 3.0 | 1.8 | 184 | 3220 | https://www.nutritionvalue.org/Fish%2C\_raw%2C\_mixed\_species%2C\_eel\_nutritional\_value.html?size=100+g | 20 | 9660 | 12 | indet | NA |
| European Eel | Anguilla anguilla | Anguillidae | Eels | Fish | Fish | Mixed water | wild | medium | small | Syltholm X (MLF00936) | Syltholm X | settlement | coastal | Late Mesolithic-early Neolithic | MesoNeo | 0.5 | 3.0 | 1.8 | 184 | 3220 | https://www.nutritionvalue.org/Fish%2C\_raw%2C\_mixed\_species%2C\_eel\_nutritional\_value.html?size=100+g | 20 | 9660 | 1 | 1 | NA |
| European Eelpout | Zoarces viviparus | Zoarcidae | Eelpouts | Fish | Fish | Marine | wild | medium | small | Annasminde IV (MLF01354) | Annasminde IV | settlement | coastal | Neolithic-Bronze age | NeoBA | 0.1 | 0.5 | 0.3 | 90 | 252 | https://www.nutritionvalue.org/Fish%2C\_raw%2C\_burbot\_nutritional\_value.html?size=100+g | 15 | 1008 | 99 | indet | NA |
| European Eelpout | Zoarces viviparus | Zoarcidae | Eelpouts | Fish | Fish | Marine | wild | medium | small | Gokartbane (MLF01333) | Gokartbane | settlement | coastal | Middle Neolithic | Neo | 0.1 | 0.5 | 0.3 | 90 | 252 | https://www.nutritionvalue.org/Fish%2C\_raw%2C\_burbot\_nutritional\_value.html?size=100+g | 15 | 1008 | 3 | indet | NA |
| European Eelpout | Zoarces viviparus | Zoarcidae | Eelpouts | Fish | Fish | Marine | wild | medium | small | Syltholm II (MLF00906-II+III) | Syltholm II | settlement | coastal | Mesolithic-Neolithic | MesoNeo | 0.1 | 0.5 | 0.3 | 90 | 252 | https://www.nutritionvalue.org/Fish%2C\_raw%2C\_burbot\_nutritional\_value.html?size=100+g | 15 | 1008 | 3 | indet | NA |
| European Flounder | Platichthys flesus | Pleuronectidae | Flounders | Fish | Fish | Mixed saltwater | wild | small | small | Annasminde IV (MLF01354) | Annasminde IV | settlement | coastal | Neolithic-Bronze age | NeoBA | 2.0 | 5.0 | 3.5 | 70 | 2450 | https://www.nutritionvalue.org/Fish%2C\_raw%2C\_flatfish\_%28flounder\_and\_sole\_species%29\_nutritional\_value.html?size=100 | 25 | 5880 | 12 | indet | NA |
| European Flounder | Platichthys flesus | Pleuronectidae | Flounders | Fish | Fish | Mixed saltwater | wild | small | small | Strandholm VI (MLF01232-I) | Strandholm VI | settlement | coastal | Bronze age | BA | 2.0 | 5.0 | 3.5 | 70 | 2450 | https://www.nutritionvalue.org/Fish%2C\_raw%2C\_flatfish\_%28flounder\_and\_sole\_species%29\_nutritional\_value.html?size=100 | 25 | 5880 | 21 | 1 | NA |
| European Flounder | Platichthys flesus | Pleuronectidae | Flounders | Fish | Fish | Mixed saltwater | wild | small | small | Syltholm II (MLF00906-II+III) | Syltholm II | settlement | coastal | Mesolithic-Neolithic | MesoNeo | 2.0 | 5.0 | 3.5 | 70 | 2450 | https://www.nutritionvalue.org/Fish%2C\_raw%2C\_flatfish\_%28flounder\_and\_sole\_species%29\_nutritional\_value.html?size=100 | 25 | 5880 | 334 | indet | NA |
| European Flounder | Platichthys flesus | Pleuronectidae | Flounders | Fish | Fish | Mixed saltwater | wild | small | small | Syltholm IX (MLF00935-II) | Syltholm IX | settlement | coastal | Early Neolithic-Early Bronze Age | Neo | 2.0 | 5.0 | 3.5 | 70 | 2450 | https://www.nutritionvalue.org/Fish%2C\_raw%2C\_flatfish\_%28flounder\_and\_sole\_species%29\_nutritional\_value.html?size=100 | 25 | 5880 | 26 | indet | NA |
| European Hedgehog | Erinaceus europeaus | Erinaceidae | Hedgehogs | Mammal | Shrews | Mixed Land | wild | small | small | Syltholm II (MLF00906-III) | Syltholm II | settlement | coastal | Mesolithic-Neolithic | MesoNeo | 0.1 | 1.6 | 0.9 | 160 | 1360 | Morin et al 2022 | 10 | 8160 | 1 | 1 | NA |
| European Perch | Perca fluviatilis | Percidae | Perches | Fish | Fish | Freshwater | wild | medium | small | Annasminde IV (MLF01354) | Annasminde IV | settlement | coastal | Neolithic-Bronze age | NeoBA | 2.0 | 5.0 | 3.5 | 91 | 3185 | https://www.nutritionvalue.org/Fish%2C\_raw%2C\_mixed\_species%2C\_perch\_nutritional\_value.html?size=100+g | 25 | 7644 | 5 | indet | NA |
| European Perch | Perca fluviatilis | Percidae | Perches | Fish | Fish | Freshwater | wild | medium | small | Strandholm VI (MLF01232-I) | Strandholm VI | settlement | coastal | Bronze age | BA | 2.0 | 5.0 | 3.5 | 91 | 3185 | https://www.nutritionvalue.org/Fish%2C\_raw%2C\_mixed\_species%2C\_perch\_nutritional\_value.html?size=100+g | 25 | 7644 | 2 | 1 | NA |
| European Perch | Perca fluviatilis | Percidae | Perches | Fish | Fish | Freshwater | wild | medium | small | Syltholm IX (MLF00935-II) | Syltholm IX | settlement | coastal | Early Neolithic-Early Bronze Age | Neo | 2.0 | 5.0 | 3.5 | 91 | 3185 | https://www.nutritionvalue.org/Fish%2C\_raw%2C\_mixed\_species%2C\_perch\_nutritional\_value.html?size=100+g | 25 | 7644 | 2 | indet | NA |
| European Perch | Perca fluviatilis | Percidae | Perches | Fish | Fish | Freshwater | wild | medium | small | Syltholm X (MLF00936) | Syltholm X | settlement | coastal | Late Mesolithic-early Neolithic | MesoNeo | 2.0 | 5.0 | 3.5 | 91 | 3185 | https://www.nutritionvalue.org/Fish%2C\_raw%2C\_mixed\_species%2C\_perch\_nutritional\_value.html?size=100+g | 25 | 7644 | 5 | indet | NA |
| European Pine Marten | Martes martes | Mustelidae | Pine martens | Mammal | Carnivores | Forest | wild | small | small | Syltholm II (MLF00906-III) | Syltholm II | settlement | coastal | Mesolithic-Neolithic | MesoNeo | 1.5 | 1.7 | 1.6 | 115 | 1840 | Morin et al 2022 | 80 | 1380 | 1 | 1 | NA |
| European Wild Cat | Felis silvestris | Felidae | Cats | Mammal | Carnivores | Forest | wild | small | small | Annasminde IV (MLF01354) | Annasminde IV | settlement | coastal | Neolithic-Bronze age | NeoBA | 3.0 | 6.0 | 4.5 | 115 | 5175 | Morin et al 2022 | 200 | 1553 | 6 | 1 | NA |
| European Wild Cat | Felis silvestris | Felidae | Cats | Mammal | Carnivores | Forest | wild | small | small | Syltholm II (MLF00906-I+II+III) | Syltholm II | settlement | coastal | Mesolithic-Neolithic | MesoNeo | 3.0 | 6.0 | 4.5 | 115 | 5175 | Morin et al 2022 | 200 | 1553 | 240 | 12 | Analysis + MNI: Pernille Bangsgaard |
| European Wild Cat | Felis silvestris | Felidae | Cats | Mammal | Carnivores | Forest | wild | small | small | Syltholm VII (MLF00933-II) | Syltholm VII | settlement | coastal | Late Palaeolithic-middle Neolithic | Neo | 3.0 | 6.0 | 4.5 | 115 | 5175 | Morin et al 2022 | 200 | 1553 | 1 | 1 | NA |
| Falcon/Caracara | Falconiformes sp. | Falconidae | Falcons/caracaras | Bird | Raptors | Land | wild | small | small | Syltholm II (MLF00906-II) | Syltholm II | settlement | coastal | Mesolithic-Neolithic | MesoNeo | 0.3 | 1.5 | 0.9 | 142 | 1278 | Morin et al 2022 & http://traditionalanimalfoods.org/birds/birds-of-prey/ (Kuhnlein & Humphries 2017) | 30 | 2556 | 2 | 1 | NA |
| Fin Whale? | Cetacea sp. (maybe Balaenoptera physalus) | Balaenopteridae | Finwhales | Marine Mammal | Cetaceans | Marine | wild | large | large | Syltholm IX (MLF00935-II) | Syltholm IX | settlement | coastal | Early Neolithic-Early Bronze Age | Neo | 40000.0 | 80000.0 | 60000.0 | 111 | 66600000 | https://www.nutritionvalue.org/Whale%2C\_raw\_%28Alaska\_Native%29%2C\_meat%2C\_beluga\_nutritional\_value.html | 7000 | 570857 | 1 | 1 | NA |
| Flatfish | P. platessa/P. flesus/L. limanda | Pleuronectiformes | Flatfishes | Fish | Fish | Marine | wild | small | small | Annas Minde II (MLF01352) | Annasminde II | settlement | coastal | Neolithic-Bronze age | NeoBA | 0.1 | 3.0 | 1.6 | 70 | 1085 | https://www.nutritionvalue.org/Fish%2C\_raw%2C\_flatfish\_%28flounder\_and\_sole\_species%29\_nutritional\_value.html?size=100 | 20 | 3255 | 5 | indet | NA |
| Flatfish | P. platessa/P. flesus/L. limanda | Pleuronectiformes | Flatfishes | Fish | Fish | Marine | wild | small | small | Annas Minde III (MLF01353) | Annasminde III | settlement | coastal | Neolithic-Bronze age | BA | 0.1 | 3.0 | 1.6 | 70 | 1085 | https://www.nutritionvalue.org/Fish%2C\_raw%2C\_flatfish\_%28flounder\_and\_sole\_species%29\_nutritional\_value.html?size=100 | 20 | 3255 | 3 | indet | NA |
| Flatfish | P. platessa/P. flesus/L. limanda | Pleuronectiformes | Flatfishes | Fish | Fish | Marine | wild | small | small | Annasminde IV (MLF01354) | Annasminde IV | settlement | coastal | Neolithic-Bronze age | NeoBA | 0.1 | 3.0 | 1.6 | 70 | 1085 | https://www.nutritionvalue.org/Fish%2C\_raw%2C\_flatfish\_%28flounder\_and\_sole\_species%29\_nutritional\_value.html?size=100 | 20 | 3255 | 687 | indet | NA |
| Flatfish | P. platessa/P. flesus/L. limanda | Pleuronectiformes | Flatfishes | Fish | Fish | Marine | wild | small | small | Annasminde V (MLF01355) | Annasminde V | settlement | coastal | Bronze age | BA | 0.1 | 3.0 | 1.6 | 70 | 1085 | https://www.nutritionvalue.org/Fish%2C\_raw%2C\_flatfish\_%28flounder\_and\_sole\_species%29\_nutritional\_value.html?size=100 | 20 | 3255 | 6 | indet | NA |
| Flatfish | P. platessa/P. flesus/L. limanda | Pleuronectiformes | Flatfishes | Fish | Fish | Marine | wild | small | small | Finlandsvej II (MLF02548) | Finlandsvej II | settlement | coastal | Neolithic-Iron Age | NeoBA | 0.1 | 3.0 | 1.6 | 70 | 1085 | https://www.nutritionvalue.org/Fish%2C\_raw%2C\_flatfish\_%28flounder\_and\_sole\_species%29\_nutritional\_value.html?size=100 | 20 | 3255 | 7 | indet | NA |
| Flatfish | P. platessa/P. flesus/L. limanda | Pleuronectiformes | Flatfishes | Fish | Fish | Marine | wild | small | small | Gokartbane (MLF01333) | Gokartbane | settlement | coastal | Middle Neolithic | Neo | 0.1 | 3.0 | 1.6 | 70 | 1085 | https://www.nutritionvalue.org/Fish%2C\_raw%2C\_flatfish\_%28flounder\_and\_sole\_species%29\_nutritional\_value.html?size=100 | 20 | 3255 | 3 | indet | NA |
| Flatfish | P. platessa/P. flesus/L. limanda | Pleuronectiformes | Flatfishes | Fish | Fish | Marine | wild | small | small | Strandholm I (MLF00909-II) | Strandholm I | settlement | coastal | Neolithic | Neo | 0.1 | 3.0 | 1.6 | 70 | 1085 | https://www.nutritionvalue.org/Fish%2C\_raw%2C\_flatfish\_%28flounder\_and\_sole\_species%29\_nutritional\_value.html?size=100 | 20 | 3255 | 9 | indet | NA |
| Flatfish | P. platessa/P. flesus/L. limanda | Pleuronectiformes | Flatfishes | Fish | Fish | Marine | wild | small | small | Strandholm VI (MLF01232-I) | Strandholm VI | settlement | coastal | Bronze age | BA | 0.1 | 3.0 | 1.6 | 70 | 1085 | https://www.nutritionvalue.org/Fish%2C\_raw%2C\_flatfish\_%28flounder\_and\_sole\_species%29\_nutritional\_value.html?size=100 | 20 | 3255 | 568 | 4 | NA |
| Flatfish | P. platessa/P. flesus/L. limanda | Pleuronectiformes | Flatfishes | Fish | Fish | Marine | wild | small | small | Syltholm II (MLF00906-II+III) | Syltholm II | settlement | coastal | Mesolithic-Neolithic | MesoNeo | 0.1 | 3.0 | 1.6 | 70 | 1085 | https://www.nutritionvalue.org/Fish%2C\_raw%2C\_flatfish\_%28flounder\_and\_sole\_species%29\_nutritional\_value.html?size=100 | 20 | 3255 | 456 | indet | NA |
| Flatfish | P. platessa/P. flesus/L. limanda | Pleuronectiformes | Flatfishes | Fish | Fish | Marine | wild | small | small | Syltholm IX (MLF00935-II) | Syltholm IX | settlement | coastal | Early Neolithic-Early Bronze Age | Neo | 0.1 | 3.0 | 1.6 | 70 | 1085 | https://www.nutritionvalue.org/Fish%2C\_raw%2C\_flatfish\_%28flounder\_and\_sole\_species%29\_nutritional\_value.html?size=100 | 20 | 3255 | 61 | indet | NA |
| Flatfish | P. platessa/P. flesus/L. limanda | Pleuronectiformes | Flatfishes | Fish | Fish | Marine | wild | small | small | Syltholm V (MLF00910) | Syltholm V | settlement | coastal | Late Mesolithic-Neolithic | MesoNeo | 0.1 | 3.0 | 1.6 | 70 | 1085 | https://www.nutritionvalue.org/Fish%2C\_raw%2C\_flatfish\_%28flounder\_and\_sole\_species%29\_nutritional\_value.html?size=100 | 20 | 3255 | 8 | indet | NA |
| Flatfish | P. platessa/P. flesus/L. limanda | Pleuronectiformes | Flatfishes | Fish | Fish | Marine | wild | small | small | Syltholm VII (MLF00933-II+III) | Syltholm VII | settlement | coastal | Late Palaeolithic-middle Neolithic | Neo | 0.1 | 3.0 | 1.6 | 70 | 1085 | https://www.nutritionvalue.org/Fish%2C\_raw%2C\_flatfish\_%28flounder\_and\_sole\_species%29\_nutritional\_value.html?size=100 | 20 | 3255 | 2 | 1 | NA |
| Flatfish | P. platessa/P. flesus/L. limanda | Pleuronectiformes | Flatfishes | Fish | Fish | Marine | wild | small | small | Syltholm X (MLF00936) | Syltholm X | settlement | coastal | Late Mesolithic-early Neolithic | MesoNeo | 0.1 | 3.0 | 1.6 | 70 | 1085 | https://www.nutritionvalue.org/Fish%2C\_raw%2C\_flatfish\_%28flounder\_and\_sole\_species%29\_nutritional\_value.html?size=100 | 20 | 3255 | 17 | indet | NA |
| Flatfish | P. platessa/P. flesus/L. limanda | Pleuronectiformes | Flatfishes | Fish | Fish | Marine | wild | small | small | Syltholm XIII (MLF00939-I+II) | Syltholm XIII | settlement | coastal | Late Mesolithic-early Neolithic | MesoNeo | 0.1 | 3.0 | 1.6 | 70 | 1085 | https://www.nutritionvalue.org/Fish%2C\_raw%2C\_flatfish\_%28flounder\_and\_sole\_species%29\_nutritional\_value.html?size=100 | 20 | 3255 | 16 | 1 | NA |
| Frog | Anura indet. | Anura | Frogs/toads | Amphibians | Amphibian | Wetland | wild | small | small | Annasminde IV (MLF01354) | Annasminde IV | settlement | coastal | Neolithic-Bronze age | NeoBA | 0.0 | 0.1 | 0.0 | 90 | 27 | Morin et al 2022 | 15 | 108 | 8 | indet | NA |
| Frog | Anura indet. | Anura | Frogs/toads | Amphibians | Amphibian | Wetland | wild | small | small | Finlandsvej II (MLF02548) | Finlandsvej II | settlement | coastal | Neolithic-Iron Age | NeoBA | 0.0 | 0.1 | 0.0 | 90 | 27 | Morin et al 2022 | 15 | 108 | 2 | 1 | NA |
| Frog | Anura sp. | Anura | Frogs/toads | Amphibians | Amphibian | Wetland | wild | small | small | Syltholm IX (MLF00935-II) | Syltholm IX | settlement | coastal | Early Neolithic-Early Bronze Age | Neo | 0.0 | 0.1 | 0.0 | 90 | 27 | Morin et al 2022 | 15 | 108 | 1 | 1 | NA |
| Frog | Anura sp. | Anura | Frogs/toads | Amphibians | Amphibian | Wetland | wild | small | small | Syltholm X (MLF00936) | Syltholm X | settlement | coastal | Late Mesolithic-early Neolithic | MesoNeo | 0.0 | 0.1 | 0.0 | 90 | 27 | Morin et al 2022 | 15 | 108 | 1 | 1 | NA |
| Frog | Anura sp. | Anura | Frogs/toads | Amphibians | Amphibian | Wetland | wild | small | small | Syltholm XIII (MLF00939-I) | Syltholm XIII | settlement | coastal | Late Mesolithic-early Neolithic | MesoNeo | 0.0 | 0.1 | 0.0 | 90 | 27 | Morin et al 2022 | 15 | 108 | 4 | 1 | NA |
| Garfish | Belone belone | Belonidae | Garfishes | Fish | Fish | Mixed saltwater | wild | small | small | Annasminde IV (MLF01354) | Annasminde IV | settlement | coastal | Neolithic-Bronze age | NeoBA | 0.5 | 2.0 | 1.3 | 107 | 1338 | https://www.matvaretabellen.no/en/garfish-raw/ | 40 | 2006 | 1 | 1 | NA |
| Garfish | Belone belone | Belonidae | Garfishes | Fish | Fish | Mixed saltwater | wild | small | small | Annasminde V (MLF01355) | Annasminde V | settlement | coastal | Bronze age | BA | 0.5 | 2.0 | 1.3 | 107 | 1338 | https://www.matvaretabellen.no/en/garfish-raw/ | 40 | 2006 | 1 | 1 | NA |
| Garfish | Belone belone | Belonidae | Garfishes | Fish | Fish | Mixed saltwater | wild | small | small | Syltholm II (MLF00906-II+III) | Syltholm II | settlement | coastal | Mesolithic-Neolithic | MesoNeo | 0.5 | 2.0 | 1.3 | 107 | 1338 | https://www.matvaretabellen.no/en/garfish-raw/ | 40 | 2006 | 3 | indet | NA |
| Garfish | Belone belone | Belonidae | Garfishes | Fish | Fish | Mixed saltwater | wild | small | small | Syltholm IX (MLF00935-III) | Syltholm IX | settlement | coastal | Early Neolithic-Early Bronze Age | Neo | 0.5 | 2.0 | 1.3 | 107 | 1338 | https://www.matvaretabellen.no/en/garfish-raw/ | 40 | 2006 | 3 | indet | NA |
| Goosander | Mergus merganser | Anatidae | Goosanders | Bird | Waterfowl | Freshwater | wild | medium | small | Syltholm XIII (MLF00939-I) | Syltholm XIII | settlement | coastal | Late Mesolithic-early Neolithic | MesoNeo | 1.0 | 2.0 | 1.5 | 130 | 1950 | Morin et al 2022 | 50 | 2340 | 1 | 1 | NA |
| Goose | Anser sp. | Anatidae | Geese | Bird | Waterfowl | Wetland | wild | large | small | Syltholm XIII (MLF00939-II) | Syltholm XIII | settlement | coastal | Late Mesolithic-early Neolithic | MesoNeo | 1.5 | 4.2 | 2.9 | 140 | 3990 | Morin et al 2022 | 100 | 2394 | 1 | 1 | NA |
| Grass Snake | Natrix natrix | Serpentes | Snakes | Reptile | Reptiles | Land | wild | small | small | Annasminde IV (MLF01354) | Annasminde IV | settlement | coastal | Neolithic-Bronze age | NeoBA | 0.1 | 2.0 | 1.0 | 108 | 1112 | https://www.nutritionvalue.org/Game\_meat%2C\_raw%2C\_deer\_nutritional\_value.html?size=100%20g | 20 | 3337 | 2 | 1 | NA |
| Green Shore Crab | Carcinus maenas | Decapoda | Crabs | Crustacean | Crustaceans | Coastal | wild | small | small | Syltholm II (MLF00906-II) | Syltholm II | settlement | coastal | Mesolithic-Neolithic | MesoNeo | 0.1 | 0.1 | 0.1 | 57 | 40 | http://traditionalanimalfoods.org/marine-invertebrates/crustaceans/ (Kuhnlein & Humphries 2017) | 10 | 239 | 1 | indet | NA |
| Grey Gurnard | Eutrigla gurnardus | Triglidae | Gurnards | Fish | Fish | Marine | wild | small | small | Syltholm II (MLF00906-II) | Syltholm II | settlement | coastal | Mesolithic-Neolithic | MesoNeo | 0.1 | 1.0 | 0.6 | 100 | 550 | NA | 15 | 2200 | 1 | indet | NA |
| Grey Seal | Halichoerus grypus | Phocoidea | Seals | Marine Mammal | True seals | Marine | wild | medium | medium | Annasminde V (MLF01355) | Annasminde V | settlement | coastal | Bronze age | BA | 100.0 | 300.0 | 200.0 | 150 | 300000 | An approximate number based on multiple species and body parts in raw form from http://traditionalanimalfoods.org/mammals/seals-sealions-walrus/page.aspx?id=6392#harp-e (Kuhnlein & Humphries 2017) | 1100 | 16364 | 2 | 1 | NA |
| Grey Seal | Halichoerus grypus | Phocoidea | Seals | Marine Mammal | True seals | Marine | wild | medium | medium | Strandholm I (MLF00909-II) | Strandholm I | settlement | coastal | Neolithic | Neo | 100.0 | 300.0 | 200.0 | 150 | 300000 | An approximate number based on multiple species and body parts in raw form from http://traditionalanimalfoods.org/mammals/seals-sealions-walrus/page.aspx?id=6392#harp-e (Kuhnlein & Humphries 2017) | 1100 | 16364 | 1 | 1 | NA |
| Grey Seal | Halichoerus grypus | Phocoidea | Seals | Marine Mammal | True seals | Marine | wild | medium | medium | Syltholm II (MLF00906-III) | Syltholm II | settlement | coastal | Mesolithic-Neolithic | MesoNeo | 100.0 | 300.0 | 200.0 | 150 | 300000 | An approximate number based on multiple species and body parts in raw form from http://traditionalanimalfoods.org/mammals/seals-sealions-walrus/page.aspx?id=6392#harp-e (Kuhnlein & Humphries 2017) | 1100 | 16364 | 1 | 1 | NA |
| Gull | Laridae sp. | Alcidae | Gulls | Bird | Waders/Shorebirds | Coastal | wild | large | small | Strandholm I (MLF00909-II) | Strandholm I | settlement | coastal | Neolithic | Neo | 0.7 | 2.4 | 1.6 | 127 | 1969 | Morin et al 2022 | 50 | 2362 | 1 | 1 | NA |
| Gull | Larus sp. | Alcidae | Gulls | Bird | Waders/Shorebirds | Coastal | wild | large | small | Syltholm II (MLF00906-I+II+III) | Syltholm II | settlement | coastal | Mesolithic-Neolithic | MesoNeo | 0.7 | 2.4 | 1.6 | 127 | 1969 | Morin et al 2022 | 50 | 2362 | 4 | 3 | NA |
| Gull | Larus sp. | Alcidae | Gulls | Bird | Waders/Shorebirds | Coastal | wild | large | small | Syltholm VII (MLF00933-III) | Syltholm VII | settlement | coastal | Late Palaeolithic-middle Neolithic | Neo | 0.7 | 2.4 | 1.6 | 127 | 1969 | Morin et al 2022 | 50 | 2362 | 1 | 1 | NA |
| Gull | Laridae sp. | Alcidae | Gulls | Bird | Waders/Shorebirds | Coastal | wild | large | small | Syltholm X (MLF00936) | Syltholm X | settlement | coastal | Late Mesolithic-early Neolithic | MesoNeo | 0.7 | 2.4 | 1.6 | 127 | 1969 | Morin et al 2022 | 50 | 2362 | 1 | 1 | NA |
| Gull | Larus sp. | Alcidae | Gulls | Bird | Waders/Shorebirds | Coastal | wild | large | small | Syltholm XIII (MLF00939-I) | Syltholm XIII | settlement | coastal | Late Mesolithic-early Neolithic | MesoNeo | 0.7 | 2.4 | 1.6 | 127 | 1969 | Morin et al 2022 | 50 | 2362 | 1 | 1 | NA |
| Harbour Porpoise | Phocoena phocoena | Phocoena | Porpoises | Marine Mammal | Cetaceans | Marine | wild | medium | medium | Strandholm V (MLF01182) | Strandholm V | settlement | coastal | Neolithic-Bronze age | Neo | 50.0 | 70.0 | 60.0 | 316 | 189600 | Kuhnlein & Humphries 2017 | 600 | 18960 | 1 | 1 | NA |
| Harbour Porpoise | Phocoena phocoena | Phocoena | Porpoises | Marine Mammal | Cetaceans | Marine | wild | medium | medium | Syltholm II (MLF00906-II+III) | Syltholm II | settlement | coastal | Mesolithic-Neolithic | MesoNeo | 50.0 | 70.0 | 60.0 | 316 | 189600 | Kuhnlein & Humphries 2017 | 600 | 18960 | 9 | 2 | NA |
| Harbour Porpoise | Phocoena phocoena | Phocoena | Porpoises | Marine Mammal | Cetaceans | Marine | wild | medium | medium | Syltholm XIII (MLF00939-I) | Syltholm XIII | settlement | coastal | Late Mesolithic-early Neolithic | MesoNeo | 50.0 | 70.0 | 60.0 | 316 | 189600 | Kuhnlein & Humphries 2017 | 1100 | 10342 | 1 | 1 | NA |
| Harbour Seal | Phoca vitulina | Phocoidea | Seals | Marine Mammal | True seals | Marine | wild | medium | medium | Syltholm II (MLF00906-III) | Syltholm II | settlement | coastal | Mesolithic-Neolithic | MesoNeo | 55.0 | 170.0 | 112.5 | 150 | 168750 | An approximate number based on multiple species and body parts in raw form from http://traditionalanimalfoods.org/mammals/seals-sealions-walrus/page.aspx?id=6392#harp-e (Kuhnlein & Humphries 2017) | 900 | 11250 | 1 | 1 | NA |
| Harp Seal | Phoca groenlandicus | Phocoidea | Seals | Marine Mammal | True seals | Marine | wild | medium | medium | Syltholm XIII (MLF00939-I) | Syltholm XIII | settlement | coastal | Late Mesolithic-early Neolithic | MesoNeo | 100.0 | 150.0 | 125.0 | 150 | 187500 | An approximate number based on multiple species and body parts in raw form from http://traditionalanimalfoods.org/mammals/seals-sealions-walrus/page.aspx?id=6392#harp-e (Kuhnlein & Humphries 2017) | 900 | 12500 | 1 | 1 | NA |
| Hen Harrier | Circus cyaneus | Accipitridae | Harriers | Bird | Raptors | Wetland | wild | medium | small | Syltholm II (MLF00906-III) | Syltholm II | settlement | coastal | Mesolithic-Neolithic | MesoNeo | 0.2 | 1.0 | 0.6 | 142 | 852 | Morin et al 2022 & http://traditionalanimalfoods.org/birds/birds-of-prey/ (Kuhnlein & Humphries 2017) | 30 | 1704 | 1 | 1 | NA |
| Heron | Ardeidae sp. | Arderidae | Herons | Bird | Waterfowl | Wetland | wild | large | small | Annas Minde II (MLF01352) | Annasminde II | settlement | coastal | Neolithic-Bronze age | NeoBA | 0.4 | 2.2 | 1.3 | 94 | 1222 | NA | 40 | 1833 | 1 | 1 | NA |
| Horse | Equus caballus | Equidae | Horses | Mammal | Ungulates | Mixed Land | indet | large | large | Annas Minde III (MLF01353) | Annasminde III | settlement | coastal | Neolithic-Bronze age | BA | 300.0 | 500.0 | 400.0 | 133 | 532000 | Morin et al 2022 & http://traditionalanimalfoods.org/mammals/domesticated/#horse-e (Kuhnlein & Humphries 2017) | 2000 | 15960 | 86 | 3 | NA |
| Horse | Equus sp. | Equidae | Horses | Mammal | Ungulates | Mixed Land | indet | large | large | Annasminde IV (MLF01354) | Annasminde IV | settlement | coastal | Neolithic-Bronze age | NeoBA | 300.0 | 500.0 | 400.0 | 133 | 532000 | Morin et al 2022 & http://traditionalanimalfoods.org/mammals/domesticated/#horse-e (Kuhnlein & Humphries 2017) | 2000 | 15960 | 167 | 2 | NA |
| Horse | Equus sp. | Equidae | Horses | Mammal | Ungulates | Mixed Land | indet | large | large | Annasminde V (MLF01355) | Annasminde V | settlement | coastal | Bronze age | BA | 300.0 | 500.0 | 400.0 | 133 | 532000 | Morin et al 2022 & http://traditionalanimalfoods.org/mammals/domesticated/#horse-e (Kuhnlein & Humphries 2017) | 2000 | 15960 | 58 | 3 | NA |
| Horse | Equus sp. | Equidae | Horses | Mammal | Ungulates | Mixed Land | indet | large | large | Finlandsvej II (MLF02548) | Finlandsvej II | settlement | coastal | Neolithic-Iron Age | NeoBA | 300.0 | 500.0 | 400.0 | 133 | 532000 | Morin et al 2022 & http://traditionalanimalfoods.org/mammals/domesticated/#horse-e (Kuhnlein & Humphries 2017) | 2000 | 15960 | 28 | 2 | NA |
| Horse | Equus ferus | Equidae | Horses | Mammal | Ungulates | Mixed Land | indet | large | large | RGS90 (MLF00952) | RGS90 | settlement | coastal | Neolithic-Bronze age | BA | 300.0 | 500.0 | 400.0 | 133 | 532000 | Morin et al 2022 & http://traditionalanimalfoods.org/mammals/domesticated/#horse-e (Kuhnlein & Humphries 2017) | 2000 | 15960 | 1 | 1 | Bangsgaard 2016 |
| Horse | Equus ferus | Equidae | Horses | Mammal | Ungulates | Mixed Land | indet | large | large | Strandholm VI (MLF01232-I) | Strandholm VI | settlement | coastal | Bronze age | BA | 300.0 | 500.0 | 400.0 | 133 | 532000 | Morin et al 2022 & http://traditionalanimalfoods.org/mammals/domesticated/#horse-e (Kuhnlein & Humphries 2017) | 2000 | 15960 | 2 | 1 | NA |
| Horse | Equus sp. | Equidae | Horses | Mammal | Ungulates | Mixed Land | indet | large | large | Syltholm IX (MLF00935-III) | Syltholm IX | settlement | coastal | Early Neolithic-Early Bronze Age | Neo | 300.0 | 500.0 | 400.0 | 133 | 532000 | Morin et al 2022 & http://traditionalanimalfoods.org/mammals/domesticated/#horse-e (Kuhnlein & Humphries 2017) | 2000 | 15960 | 1 | 1 | NA |
| Lark | Alaudidae sp. | Alaudidae | Larks | Bird | Landbirds | Land | wild | small | small | Syltholm XIII (MLF00939-I) | Syltholm XIII | settlement | coastal | Late Mesolithic-early Neolithic | MesoNeo | 0.0 | 0.8 | 0.4 | 94 | 357 | NA | 15 | 1429 | 1 | 1 | NA |
| Long-Tailed Duck | Clangula hyemalis | Anatidae | Ducks | Bird | Waterfowl | Coastal | wild | small | small | Syltholm II (MLF00906-III) | Syltholm II | settlement | coastal | Mesolithic-Neolithic | MesoNeo | 0.5 | 1.5 | 1.0 | 227 | 2270 | http://traditionalanimalfoods.org/birds/waterfowl/page.aspx?id=6456 (Kuhnlein & Humphries 2017) | 30 | 4540 | 3 | 1 | NA |
| Mackerel Shark | Lamniformes | Lamniformes | Mackerel sharks | Fish | Saltwater fish | Marine | wild | large | medium | Syltholm IX (MLF00935-I) | Syltholm IX | settlement | coastal | Early Neolithic-Early Bronze Age | Neo | 60.0 | 140.0 | 100.0 | 130 | 130000 | https://www.nutritionvalue.org/Fish%2C\_raw%2C\_mixed\_species%2C\_shark\_nutritional\_value.html?size=100+g | 800 | 9750 | 3 | 1 | NA |
| Mouse | Muridae | Muridae | Small rodents | Mammal | Rodents | Forest | wild | small | small | Annas Minde III (MLF01353) | Annasminde III | settlement | coastal | Neolithic-Bronze age | BA | 0.0 | 0.1 | 0.1 | 123 | 74 | Morin et al 2022 | 15 | 295 | 2 | 1 | NA |
| Mouse | Muridae | Muridae | Small rodents | Mammal | Rodents | Forest | wild | small | small | Annasminde IV (MLF01354) | Annasminde IV | settlement | coastal | Neolithic-Bronze age | NeoBA | 0.0 | 0.1 | 0.1 | 123 | 74 | Morin et al 2022 | 15 | 295 | 1 | 1 | NA |
| Mouse | Muridae | Muridae | Small rodents | Mammal | Rodents | Forest | wild | small | small | Syltholm II (MLF00906-I+II+III) | Syltholm II | settlement | coastal | Mesolithic-Neolithic | MesoNeo | 0.0 | 0.1 | 0.1 | 123 | 74 | Morin et al 2022 | 15 | 295 | 16 | 5 | NA |
| Mouse | Muridae | Muridae | Small rodents | Mammal | Rodents | Forest | wild | small | small | Syltholm VII (MLF00933-II) | Syltholm VII | settlement | coastal | Late Palaeolithic-middle Neolithic | Neo | 0.0 | 0.1 | 0.1 | 123 | 74 | Morin et al 2022 | 15 | 295 | 5 | 1 | NA |
| Mouse | Muridae | Muridae | Small rodents | Mammal | Rodents | Forest | wild | small | small | Syltholm X (MLF00936) | Syltholm X | settlement | coastal | Late Mesolithic-early Neolithic | MesoNeo | 0.0 | 0.1 | 0.1 | 123 | 74 | Morin et al 2022 | 15 | 295 | 1 | 1 | NA |
| Northern Gannet | Morus bassanus | Sulidae | Gannets | Bird | Seabirds | Coastal | wild | large | small | Syltholm II (MLF00906-I) | Syltholm II | settlement | coastal | Mesolithic-Neolithic | MesoNeo | 2.3 | 3.6 | 3.0 | 142 | 4189 | Morin et al 2022 estimate from various bird species of similar size and biome | 100 | 2513 | 5 | 1 | Analysis: Pernille Bangsgaard |
| Old World Flycatcher | Muscicapidae | Muscicapidae | Flycatchers | Bird | Landbirds | Forest | wild | small | small | Syltholm XIII (MLF00939-I) | Syltholm XIII | settlement | coastal | Late Mesolithic-early Neolithic | MesoNeo | 0.0 | 0.0 | 0.0 | 94 | 19 | NA | 15 | 75 | 1 | 1 | NA |
| Orca | Orcinus orca | Delphinidae | Orcas | Marine Mammal | Cetaceans | Marine | wild | large | large | Syltholm IX (MLF00935-III) | Syltholm IX | settlement | coastal | Early Neolithic-Early Bronze Age | Neo | 1300.0 | 10000.0 | 5650.0 | 150 | 8475000 | Used value for seals from Kuhnlein & Humphries 2017 | 5000 | 101700 | 1 | 1 | NA |
| Passerine | Passeriformes | Passeriformes | Passerines | Bird | Landbirds | Forest | wild | small | small | Syltholm II (MLF00906-II+III) | Syltholm II | settlement | coastal | Mesolithic-Neolithic | MesoNeo | 0.3 | 2.5 | 1.4 | 127 | 1778 | Morin et al 2022 | 50 | 2134 | 13 | 3 | NA |
| Passerine | Passeriformes | Passeriformes | Passerines | Bird | Landbirds | Forest | wild | small | small | Syltholm XIII (MLF00939-I+II) | Syltholm XIII | settlement | coastal | Late Mesolithic-early Neolithic | MesoNeo | 0.3 | 2.5 | 1.4 | 127 | 1778 | Morin et al 2022 | 50 | 2134 | 3 | 2 | NA |
| Pig | Sus sp. | Suidae | Wild boars/domestic pigs | Mammal | Ungulates | Mixed Land | indet | medium | medium | Annas Minde II (MLF01352) | Annasminde II | settlement | coastal | Neolithic-Bronze age | NeoBA | 50.0 | 200.0 | 125.0 | 122 | 152500 | https://www.nutritionvalue.org/Game\_meat%2C\_raw%2C\_wild%2C\_boar\_nutritional\_value.html?size=100+g | 900 | 10167 | 93 | 1 | NA |
| Pig | Sus sp. | Suidae | Wild boars/domestic pigs | Mammal | Ungulates | Mixed Land | indet | medium | medium | Annasminde IV (MLF01354) | Annasminde IV | settlement | coastal | Neolithic-Bronze age | NeoBA | 50.0 | 200.0 | 125.0 | 122 | 152500 | https://www.nutritionvalue.org/Game\_meat%2C\_raw%2C\_wild%2C\_boar\_nutritional\_value.html?size=100+g | 900 | 10167 | 128 | 2 | NA |
| Pig | Sus sp. | Suidae | Wild boars/domestic pigs | Mammal | Ungulates | Mixed Land | indet | medium | medium | Annasminde V (MLF01355) | Annasminde V | settlement | coastal | Bronze age | BA | 50.0 | 200.0 | 125.0 | 122 | 152500 | https://www.nutritionvalue.org/Game\_meat%2C\_raw%2C\_wild%2C\_boar\_nutritional\_value.html?size=100+g | 900 | 10167 | 44 | 1 | NA |
| Pig | Sus sp. | Suidae | Wild boars/domestic pigs | Mammal | Ungulates | Mixed Land | indet | medium | medium | Finlandsvej II (MLF02548) | Finlandsvej II | settlement | coastal | Neolithic-Iron Age | NeoBA | 50.0 | 200.0 | 125.0 | 122 | 152500 | https://www.nutritionvalue.org/Game\_meat%2C\_raw%2C\_wild%2C\_boar\_nutritional\_value.html?size=100+g | 900 | 10167 | 10 | 1 | NA |
| Pig | Sus sp. | Suidae | Wild boars/domestic pigs | Mammal | Ungulates | Mixed Land | indet | medium | medium | Gokartbane (MLF01333) | Gokartbane | settlement | coastal | Middle Neolithic | Neo | 50.0 | 200.0 | 125.0 | 122 | 152500 | https://www.nutritionvalue.org/Game\_meat%2C\_raw%2C\_wild%2C\_boar\_nutritional\_value.html?size=100+g | 900 | 10167 | 43 | 2 | NA |
| Pig | Sus sp. | Suidae | Wild boars/domestic pigs | Mammal | Ungulates | Mixed Land | indet | medium | medium | RGS90 (MLF00952) | RGS90 | settlement | coastal | Neolithic-Bronze age | BA | 50.0 | 200.0 | 125.0 | 122 | 152500 | https://www.nutritionvalue.org/Game\_meat%2C\_raw%2C\_wild%2C\_boar\_nutritional\_value.html?size=100+g | 900 | 10167 | 6 | 1 | Bangsgaard 2016 |
| Pig | Sus sp. | Suidae | Wild boars/domestic pigs | Mammal | Ungulates | Mixed Land | indet | medium | medium | Strandholm I (MLF00909-I+II) | Strandholm I | settlement | coastal | Neolithic | Neo | 50.0 | 200.0 | 125.0 | 122 | 152500 | https://www.nutritionvalue.org/Game\_meat%2C\_raw%2C\_wild%2C\_boar\_nutritional\_value.html?size=100+g | 900 | 10167 | 36 | 3 | NA |
| Pig | Sus sp. | Suidae | Wild boars/domestic pigs | Mammal | Ungulates | Mixed Land | indet | medium | medium | Strandholm V (MLF01182) | Strandholm V | settlement | coastal | Neolithic-Bronze age | Neo | 50.0 | 200.0 | 125.0 | 122 | 152500 | https://www.nutritionvalue.org/Game\_meat%2C\_raw%2C\_wild%2C\_boar\_nutritional\_value.html?size=100+g | 900 | 10167 | 13 | 2 | NA |
| Pig | Sus sp. | Suidae | Wild boars/domestic pigs | Mammal | Ungulates | Mixed Land | indet | medium | medium | Strandholm VI (MLF01232-I) | Strandholm VI | settlement | coastal | Bronze age | BA | 50.0 | 200.0 | 125.0 | 122 | 152500 | https://www.nutritionvalue.org/Game\_meat%2C\_raw%2C\_wild%2C\_boar\_nutritional\_value.html?size=100+g | 900 | 10167 | 4 | 2 | NA |
| Pig | Sus sp. | Suidae | Wild boars/domestic pigs | Mammal | Ungulates | Mixed Land | indet | medium | medium | Syltholm II (MLF00906-I+II+III) | Syltholm II | settlement | coastal | Mesolithic-Neolithic | MesoNeo | 50.0 | 200.0 | 125.0 | 122 | 152500 | https://www.nutritionvalue.org/Game\_meat%2C\_raw%2C\_wild%2C\_boar\_nutritional\_value.html?size=100+g | 900 | 10167 | 310 | 13 | Analysis + MNI: Pernille Bangsgaard |
| Pig | Sus sp. | Suidae | Wild boars/domestic pigs | Mammal | Ungulates | Mixed Land | indet | medium | medium | Syltholm IX (MLF00935-I+II+III) | Syltholm IX | settlement | coastal | Early Neolithic-Early Bronze Age | Neo | 50.0 | 200.0 | 125.0 | 122 | 152500 | https://www.nutritionvalue.org/Game\_meat%2C\_raw%2C\_wild%2C\_boar\_nutritional\_value.html?size=100+g | 900 | 10167 | 63 | 6 | NA |
| Pig | Sus sp. | Suidae | Wild boars/domestic pigs | Mammal | Ungulates | Mixed Land | indet | medium | medium | Syltholm V (MLF00910) | Syltholm V | settlement | coastal | Late Mesolithic-Neolithic | MesoNeo | 50.0 | 200.0 | 125.0 | 122 | 152500 | https://www.nutritionvalue.org/Game\_meat%2C\_raw%2C\_wild%2C\_boar\_nutritional\_value.html?size=100+g | 900 | 10167 | 1 | 1 | NA |
| Pig | Sus sp. | Suidae | Wild boars/domestic pigs | Mammal | Ungulates | Mixed Land | indet | medium | medium | Syltholm VII (MLF00933-II) | Syltholm VII | settlement | coastal | Late Palaeolithic-middle Neolithic | Neo | 50.0 | 200.0 | 125.0 | 122 | 152500 | https://www.nutritionvalue.org/Game\_meat%2C\_raw%2C\_wild%2C\_boar\_nutritional\_value.html?size=100+g | 900 | 10167 | 8 | 1 | NA |
| Pig | Sus sp. | Suidae | Wild boars/domestic pigs | Mammal | Ungulates | Mixed Land | indet | medium | medium | Syltholm X (MLF00936) | Syltholm X | settlement | coastal | Late Mesolithic-early Neolithic | MesoNeo | 50.0 | 200.0 | 125.0 | 122 | 152500 | https://www.nutritionvalue.org/Game\_meat%2C\_raw%2C\_wild%2C\_boar\_nutritional\_value.html?size=100+g | 900 | 10167 | 66 | 3 | NA |
| Pig | Sus sp. | Suidae | Wild boars/domestic pigs | Mammal | Ungulates | Mixed Land | indet | medium | medium | Syltholm XIII (MLF00939-I+II) | Syltholm XIII | settlement | coastal | Late Mesolithic-early Neolithic | MesoNeo | 50.0 | 200.0 | 125.0 | 122 | 152500 | https://www.nutritionvalue.org/Game\_meat%2C\_raw%2C\_wild%2C\_boar\_nutritional\_value.html?size=100+g | 900 | 10167 | 14 | 2 | NA |
| Pig | Sus sp. | Suidae | Wild boars/domestic pigs | Mammal | Ungulates | Mixed Land | indet | medium | medium | Syltholm XIV (MLF00940) | Syltholm XIV | settlement | coastal | Early Mesolithic-Late Mesolithic | Meso | 50.0 | 200.0 | 125.0 | 122 | 152500 | https://www.nutritionvalue.org/Game\_meat%2C\_raw%2C\_wild%2C\_boar\_nutritional\_value.html?size=100+g | 900 | 10167 | 24 | 3 | NA |
| Pike | Esox lucius | Esocidae | Pikes | Fish | Fish | Freshwater | wild | large | small | Strandholm I (MLF00909-II) | Strandholm I | settlement | coastal | Neolithic | Neo | 2.0 | 20.0 | 11.0 | 85 | 9350 | https://www.nutritionvalue.org/Fish%2C\_raw%2C\_northern%2C\_pike\_nutritional\_value.html?size=100+g OR https://www.nutritionvalue.org/Fish%2C\_raw%2C\_northern%2C\_pike\_nutritional\_value.html?size=100+g | 50 | 11220 | 1 | 1 | NA |
| Pike | Esox lucius | Esocidae | Pikes | Fish | Fish | Freshwater | wild | large | small | Strandholm VI (MLF01232-I) | Strandholm VI | settlement | coastal | Bronze age | BA | 2.0 | 20.0 | 11.0 | 85 | 9350 | https://www.nutritionvalue.org/Fish%2C\_raw%2C\_northern%2C\_pike\_nutritional\_value.html?size=100+g OR https://www.nutritionvalue.org/Fish%2C\_raw%2C\_northern%2C\_pike\_nutritional\_value.html?size=100+g | 50 | 11220 | 1 | 1 | NA |
| Pike | Esox lucius | Esocidae | Pikes | Fish | Fish | Freshwater | wild | large | small | Syltholm IX (MLF00935-II) | Syltholm IX | settlement | coastal | Early Neolithic-Early Bronze Age | Neo | 2.0 | 20.0 | 11.0 | 85 | 9350 | https://www.nutritionvalue.org/Fish%2C\_raw%2C\_northern%2C\_pike\_nutritional\_value.html?size=100+g OR https://www.nutritionvalue.org/Fish%2C\_raw%2C\_northern%2C\_pike\_nutritional\_value.html?size=100+g | 50 | 11220 | 1 | indet | NA |
| Plover/Dotterel/Lapwing | Charadriidae sp. | Charadriidae | Plovers/dotterels/lapwings | Bird | Waders/Shorebirds | Coastal | wild | small | small | Syltholm II (MLF00906-I) | Syltholm II | settlement | coastal | Mesolithic-Neolithic | MesoNeo | 0.1 | 0.5 | 0.3 | 94 | 263 | NA | 15 | 1053 | 1 | 1 | Analysis: Pernille Bangsgaard |
| Rat | Rattus sp. | Muridae | Small rodents | Mammal | Rodents | Land | wild | medium | small | Annasminde IV (MLF01354) | Annasminde IV | settlement | coastal | Neolithic-Bronze age | NeoBA | 0.0 | 0.1 | 0.1 | 123 | 74 | Morin et al 2022 | 15 | 295 | 1 | 1 | NA |
| Rat | Rattus sp. | Muridae | Small rodents | Mammal | Rodents | Land | wild | medium | small | Syltholm IX (MLF00935-I) | Syltholm IX | settlement | coastal | Early Neolithic-Early Bronze Age | Neo | 0.0 | 0.1 | 0.1 | 123 | 74 | Morin et al 2022 | 15 | 295 | 1 | 1 | NA |
| Red Deer | Cervus elaphus | Cervidae | Red deer | Mammal | Ungulates | Mixed Land | wild | large | large | Annas Minde II (MLF01352) | Annasminde II | settlement | coastal | Neolithic-Bronze age | NeoBA | 120.0 | 240.0 | 180.0 | 120 | 216000 | https://www.nutritionvalue.org/Game\_meat%2C\_raw%2C\_deer\_nutritional\_value.html?size=100%20g | 950 | 13642 | 2 | 1 | NA |
| Red Deer | Cervus elaphus | Cervidae | Red deer | Mammal | Ungulates | Mixed Land | wild | large | large | Annasminde IV (MLF01354) | Annasminde IV | settlement | coastal | Neolithic-Bronze age | NeoBA | 120.0 | 240.0 | 180.0 | 120 | 216000 | https://www.nutritionvalue.org/Game\_meat%2C\_raw%2C\_deer\_nutritional\_value.html?size=100%20g | 950 | 13642 | 5 | 1 | NA |
| Red Deer | Cervus elaphus | Cervidae | Red deer | Mammal | Ungulates | Mixed Land | wild | large | large | Annasminde V (MLF01355) | Annasminde V | settlement | coastal | Bronze age | BA | 120.0 | 240.0 | 180.0 | 120 | 216000 | https://www.nutritionvalue.org/Game\_meat%2C\_raw%2C\_deer\_nutritional\_value.html?size=100%20g | 950 | 13642 | 2 | 1 | NA |
| Red Deer | Cervus elaphus | Cervidae | Red deer | Mammal | Ungulates | Mixed Land | wild | large | large | Finlandsvej II (MLF02548) | Finlandsvej II | settlement | coastal | Neolithic-Iron Age | NeoBA | 120.0 | 240.0 | 180.0 | 120 | 216000 | https://www.nutritionvalue.org/Game\_meat%2C\_raw%2C\_deer\_nutritional\_value.html?size=100%20g | 950 | 13642 | 1 | 1 | NA |
| Red Deer | Cervus elaphus | Cervidae | Red deer | Mammal | Ungulates | Mixed Land | wild | large | large | Strandholm I (MLF00909-I+II) | Strandholm I | settlement | coastal | Neolithic | Neo | 120.0 | 240.0 | 180.0 | 120 | 216000 | https://www.nutritionvalue.org/Game\_meat%2C\_raw%2C\_deer\_nutritional\_value.html?size=100%20g | 950 | 13642 | 21 | 2 | NA |
| Red Deer | Cervus elaphus | Cervidae | Red deer | Mammal | Ungulates | Mixed Land | wild | large | large | Syltholm II (MLF00906-I+II+III) | Syltholm II | settlement | coastal | Mesolithic-Neolithic | MesoNeo | 120.0 | 240.0 | 180.0 | 120 | 216000 | https://www.nutritionvalue.org/Game\_meat%2C\_raw%2C\_deer\_nutritional\_value.html?size=100%20g | 950 | 13642 | 190 | 10 | Analysis + MNI: Pernille Bangsgaard |
| Red Deer | Cervus elaphus | Cervidae | Red deer | Mammal | Ungulates | Mixed Land | wild | large | large | Syltholm IX (MLF00935-I+II+III) | Syltholm IX | settlement | coastal | Early Neolithic-Early Bronze Age | Neo | 120.0 | 240.0 | 180.0 | 120 | 216000 | https://www.nutritionvalue.org/Game\_meat%2C\_raw%2C\_deer\_nutritional\_value.html?size=100%20g | 950 | 13642 | 15 | 3 | NA |
| Red Deer | Cervus elaphus | Cervidae | Red deer | Mammal | Ungulates | Mixed Land | wild | large | large | Syltholm VII (MLF00933-II+III) | Syltholm VII | settlement | coastal | Late Palaeolithic-middle Neolithic | Neo | 120.0 | 240.0 | 180.0 | 120 | 216000 | https://www.nutritionvalue.org/Game\_meat%2C\_raw%2C\_deer\_nutritional\_value.html?size=100%20g | 950 | 13642 | 28 | 2 | NA |
| Red Deer | Cervus elaphus | Cervidae | Red deer | Mammal | Ungulates | Mixed Land | wild | large | large | Syltholm X (MLF00936) | Syltholm X | settlement | coastal | Late Mesolithic-early Neolithic | MesoNeo | 120.0 | 240.0 | 180.0 | 120 | 216000 | https://www.nutritionvalue.org/Game\_meat%2C\_raw%2C\_deer\_nutritional\_value.html?size=100%20g | 950 | 13642 | 3 | 1 | NA |
| Red Deer | Cervus elaphus | Cervidae | Red deer | Mammal | Ungulates | Mixed Land | wild | large | large | Syltholm XIII (MLF00939-I) | Syltholm XIII | settlement | coastal | Late Mesolithic-early Neolithic | MesoNeo | 120.0 | 240.0 | 180.0 | 120 | 216000 | https://www.nutritionvalue.org/Game\_meat%2C\_raw%2C\_deer\_nutritional\_value.html?size=100%20g | 950 | 13642 | 7 | 1 | NA |
| Red Deer | Cervus elaphus | Cervidae | Red deer | Mammal | Ungulates | Mixed Land | wild | large | large | Syltholm XIV (MLF00940) | Syltholm XIV | settlement | coastal | Early Mesolithic-Late Mesolithic | Meso | 120.0 | 240.0 | 180.0 | 120 | 216000 | https://www.nutritionvalue.org/Game\_meat%2C\_raw%2C\_deer\_nutritional\_value.html?size=100%20g | 950 | 13642 | 36 | 4 | NA |
| Red Fox | Vulpes vulpes | Canidae | Red foxes | Mammal | Carnivores | Land | wild | small | small | Annasminde IV (MLF01354) | Annasminde IV | settlement | coastal | Neolithic-Bronze age | NeoBA | 4.5 | 11.0 | 7.8 | 118 | 9145 | Morin et al 2022 & http://traditionalanimalfoods.org/mammals/furbearers/page.aspx?id=6367 (Kuhnlein & Humphries 2017) | 300 | 1829 | 28 | 1 | NA |
| Red Fox | Vulpes vulpes | Canidae | Red foxes | Mammal | Carnivores | Land | wild | small | small | Syltholm II (MLF00906-I+III) | Syltholm II | settlement | coastal | Mesolithic-Neolithic | MesoNeo | 4.5 | 11.0 | 7.8 | 118 | 9145 | Morin et al 2022 & http://traditionalanimalfoods.org/mammals/furbearers/page.aspx?id=6367 (Kuhnlein & Humphries 2017) | 300 | 1829 | 18 | 3 | Analysis + MNI: Pernille Bangsgaard |
| Red Squirrel | Sciurus vulgaris | Sciuridae | Squirrels | Mammal | Rodents | Forest | wild | small | small | Syltholm II (MLF00906-II) | Syltholm II | settlement | coastal | Mesolithic-Neolithic | MesoNeo | 0.0 | 0.1 | 0.1 | 117 | 70 | Morin et al 2022 | 15 | 281 | 1 | 1 | NA |
| Red Squirrel | Sciurus vulgaris | Sciuridae | Squirrels | Mammal | Rodents | Forest | wild | small | small | Syltholm VII (MLF00933-II) | Syltholm VII | settlement | coastal | Late Palaeolithic-middle Neolithic | Neo | 0.0 | 0.1 | 0.1 | 117 | 70 | Morin et al 2022 | 15 | 281 | 2 | 1 | NA |
| Red-Throated Diver | Gavia stellata | Gaviidae | Divers | Bird | Waterfowl | Coastal | wild | medium | small | Syltholm II (MLF00906-III) | Syltholm II | settlement | coastal | Mesolithic-Neolithic | MesoNeo | 1.0 | 2.7 | 1.9 | 100 | 1850 |  | 70 | 1586 | 1 | 1 | NA |
| Ringed Seal | Phoca hispida | Phocoidea | Seals | Marine Mammal | True seals | Marine | wild | medium | medium | Syltholm II (MLF00906-III) | Syltholm II | settlement | coastal | Mesolithic-Neolithic | MesoNeo | 55.0 | 170.0 | 112.5 | 150 | 168750 | An approximate number based on multiple species and body parts in raw form from http://traditionalanimalfoods.org/mammals/seals-sealions-walrus/page.aspx?id=6392#harp-e (Kuhnlein & Humphries 2017) | 900 | 11250 | 3 | 1 | NA |
| Ringed Seal | Phoca hispida | Phocoidea | Seals | Marine Mammal | True seals | Marine | wild | medium | medium | Syltholm IX (MLF00935-I) | Syltholm IX | settlement | coastal | Early Neolithic-Early Bronze Age | Neo | 55.0 | 170.0 | 112.5 | 150 | 168750 | An approximate number based on multiple species and body parts in raw form from http://traditionalanimalfoods.org/mammals/seals-sealions-walrus/page.aspx?id=6392#harp-e (Kuhnlein & Humphries 2017) | 900 | 11250 | 1 | 1 | NA |
| Roe Deer | Capreolus capreolus | Cervidae | Roe deer | Mammal | Ungulates | Mixed Land | wild | medium | medium | Annas Minde II (MLF01352) | Annasminde II | settlement | coastal | Neolithic-Bronze age | NeoBA | 19.0 | 34.0 | 26.5 | 120 | 31800 | https://www.nutritionvalue.org/Game\_meat%2C\_raw%2C\_deer\_nutritional\_value.html?size=100%20g | 500 | 3816 | 2 | 1 | NA |
| Roe Deer | Capreolus capreolus | Cervidae | Roe deer | Mammal | Ungulates | Mixed Land | wild | medium | medium | Annas Minde III (MLF01353) | Annasminde III | settlement | coastal | Neolithic-Bronze age | BA | 19.0 | 34.0 | 26.5 | 120 | 31800 | https://www.nutritionvalue.org/Game\_meat%2C\_raw%2C\_deer\_nutritional\_value.html?size=100%20g | 500 | 3816 | 5 | 1 | NA |
| Roe Deer | Capreolus capreolus | Cervidae | Roe deer | Mammal | Ungulates | Mixed Land | wild | medium | medium | Annasminde IV (MLF01354) | Annasminde IV | settlement | coastal | Neolithic-Bronze age | NeoBA | 19.0 | 34.0 | 26.5 | 120 | 31800 | https://www.nutritionvalue.org/Game\_meat%2C\_raw%2C\_deer\_nutritional\_value.html?size=100%20g | 500 | 3816 | 3 | 2 | NA |
| Roe Deer | Capreolus capreolus | Cervidae | Roe deer | Mammal | Ungulates | Mixed Land | wild | medium | medium | Gokartbane (MLF01333) | Gokartbane | settlement | coastal | Middle Neolithic | Neo | 19.0 | 34.0 | 26.5 | 120 | 31800 | https://www.nutritionvalue.org/Game\_meat%2C\_raw%2C\_deer\_nutritional\_value.html?size=100%20g | 500 | 3816 | 2 | 1 | NA |
| Roe Deer | Capreolus capreolus | Cervidae | Roe deer | Mammal | Ungulates | Mixed Land | wild | medium | medium | Strandholm I (MLF00909-II) | Strandholm I | settlement | coastal | Neolithic | Neo | 19.0 | 34.0 | 26.5 | 120 | 31800 | https://www.nutritionvalue.org/Game\_meat%2C\_raw%2C\_deer\_nutritional\_value.html?size=100%20g | 500 | 3816 | 8 | 1 | NA |
| Roe Deer | Capreolus capreolus | Cervidae | Roe deer | Mammal | Ungulates | Mixed Land | wild | medium | medium | Syltholm II (MLF00906-I+II+III) | Syltholm II | settlement | coastal | Mesolithic-Neolithic | MesoNeo | 19.0 | 34.0 | 26.5 | 120 | 31800 | https://www.nutritionvalue.org/Game\_meat%2C\_raw%2C\_deer\_nutritional\_value.html?size=100%20g | 500 | 3816 | 112 | 7 | Analysis + MNI: Pernille Bangsgaard |
| Roe Deer | Capreolus capreolus | Cervidae | Roe deer | Mammal | Ungulates | Mixed Land | wild | medium | medium | Syltholm IX (MLF00935-I+II+III) | Syltholm IX | settlement | coastal | Early Neolithic-Early Bronze Age | Neo | 19.0 | 34.0 | 26.5 | 120 | 31800 | https://www.nutritionvalue.org/Game\_meat%2C\_raw%2C\_deer\_nutritional\_value.html?size=100%20g | 500 | 3816 | 10 | 3 | NA |
| Roe Deer | Capreolus capreolus | Cervidae | Roe deer | Mammal | Ungulates | Mixed Land | wild | medium | medium | Syltholm VII (MLF00933-II) | Syltholm VII | settlement | coastal | Late Palaeolithic-middle Neolithic | Neo | 19.0 | 34.0 | 26.5 | 120 | 31800 | https://www.nutritionvalue.org/Game\_meat%2C\_raw%2C\_deer\_nutritional\_value.html?size=100%20g | 500 | 3816 | 8 | 1 | NA |
| Roe Deer | Capreolus capreolus | Cervidae | Roe deer | Mammal | Ungulates | Mixed Land | wild | medium | medium | Syltholm X (MLF00936) | Syltholm X | settlement | coastal | Late Mesolithic-early Neolithic | MesoNeo | 19.0 | 34.0 | 26.5 | 120 | 31800 | https://www.nutritionvalue.org/Game\_meat%2C\_raw%2C\_deer\_nutritional\_value.html?size=100%20g | 500 | 3816 | 4 | 1 | NA |
| Roe Deer | Capreolus capreolus | Cervidae | Roe deer | Mammal | Ungulates | Mixed Land | wild | medium | medium | Syltholm XIII (MLF00939-I+II) | Syltholm XIII | settlement | coastal | Late Mesolithic-early Neolithic | MesoNeo | 19.0 | 34.0 | 26.5 | 120 | 31800 | https://www.nutritionvalue.org/Game\_meat%2C\_raw%2C\_deer\_nutritional\_value.html?size=100%20g | 500 | 3816 | 6 | 2 | NA |
| Roe Deer | Capreolus capreolus | Cervidae | Roe deer | Mammal | Ungulates | Mixed Land | wild | medium | medium | Syltholm XIV (MLF00940) | Syltholm XIV | settlement | coastal | Early Mesolithic-Late Mesolithic | Meso | 19.0 | 34.0 | 26.5 | 120 | 31800 | https://www.nutritionvalue.org/Game\_meat%2C\_raw%2C\_deer\_nutritional\_value.html?size=100%20g | 500 | 3816 | 18 | 3 | NA |
| Scoter | Melanitta sp | Anatidae | Scoters | Bird | Waterfowl | Coastal | wild | small | small | Syltholm II (MLF00906-III) | Syltholm II | settlement | coastal | Mesolithic-Neolithic | MesoNeo | 0.9 | 1.1 | 1.0 | 84 | 840 | Morin et al 2022 | 30 | 1680 | 1 | 1 | NA |
| Seahorse/Pipefish/Seadragon | Syngnathidae | Syngnathidae | Seahorses/pipefishes/seadragons | Fish | Fish | Marine | wild | small | small | Syltholm VII (MLF00933-II) | Syltholm VII | settlement | coastal | Late Palaeolithic-middle Neolithic | Neo | 0.1 | 0.2 | 0.1 | 50 | 65 | Inferred value | 15 | 260 | 5 | indet | NA |
| Seal | Phoca/Halichoerus sp | Phocoidea | Seals | Marine Mammal | True seals | Marine | wild | medium | medium | Strandholm I (MLF00909-II) | Strandholm I | settlement | coastal | Neolithic | Neo | 90.0 | 300.0 | 195.0 | 150 | 292500 | An approximate number based on multiple species and body parts in raw form from http://traditionalanimalfoods.org/mammals/seals-sealions-walrus/page.aspx?id=6392#harp-e (Kuhnlein & Humphries 2017) | 1100 | 15955 | 3 | 2 | NA |
| Seal | Phoca sp. | Phocoidea | Seals | Marine Mammal | True seals | Marine | wild | medium | medium | Strandholm V (MLF01182) | Strandholm V | settlement | coastal | Neolithic-Bronze age | Neo | 90.0 | 300.0 | 195.0 | 150 | 292500 | An approximate number based on multiple species and body parts in raw form from http://traditionalanimalfoods.org/mammals/seals-sealions-walrus/page.aspx?id=6392#harp-e (Kuhnlein & Humphries 2017) | 1100 | 15955 | 1 | 1 | NA |
| Seal | Phoca sp. | Phocoidea | Seals | Marine Mammal | True seals | Marine | wild | medium | medium | Syltholm II (MLF00906-II+III) | Syltholm II | settlement | coastal | Mesolithic-Neolithic | MesoNeo | 90.0 | 300.0 | 195.0 | 150 | 292500 | An approximate number based on multiple species and body parts in raw form from http://traditionalanimalfoods.org/mammals/seals-sealions-walrus/page.aspx?id=6392#harp-e (Kuhnlein & Humphries 2017) | 1100 | 15955 | 13 | 2 | NA |
| Seal | Phoca/Halichoerus sp | Phocoidea | Seals | Marine Mammal | True seals | Marine | wild | medium | medium | Syltholm IX (MLF00935-II) | Syltholm IX | settlement | coastal | Early Neolithic-Early Bronze Age | Neo | 90.0 | 300.0 | 195.0 | 150 | 292500 | An approximate number based on multiple species and body parts in raw form from http://traditionalanimalfoods.org/mammals/seals-sealions-walrus/page.aspx?id=6392#harp-e (Kuhnlein & Humphries 2017) | 1100 | 15955 | 1 | 1 | NA |
| Seal | Phoca sp. | Phocoidea | Seals | Marine Mammal | True seals | Marine | wild | medium | medium | Syltholm XIII (MLF00939-I+II) | Syltholm XIII | settlement | coastal | Late Mesolithic-early Neolithic | MesoNeo | 90.0 | 300.0 | 195.0 | 150 | 292500 | An approximate number based on multiple species and body parts in raw form from http://traditionalanimalfoods.org/mammals/seals-sealions-walrus/page.aspx?id=6392#harp-e (Kuhnlein & Humphries 2017) | 1100 | 15955 | 13 | 2 | NA |
| Sheep/Goat | Ovis/capra | Bovidae | Sheep/goats | Mammal | Ungulates | Mixed Land | domestic | medium | medium | Annas Minde II (MLF01352) | Annasminde II | settlement | coastal | Neolithic-Bronze age | NeoBA | 45.0 | 100.0 | 72.5 | 109 | 79025 | https://www.nutritionvalue.org/Game\_meat%2C\_raw%2C\_goat\_nutritional\_value.html?size=100+g | 700 | 6774 | 87 | 3 | NA |
| Sheep/Goat | Ovis/capra | Bovidae | Sheep/goats | Mammal | Ungulates | Mixed Land | domestic | medium | medium | Annas Minde III (MLF01353) | Annasminde III | settlement | coastal | Neolithic-Bronze age | BA | 45.0 | 100.0 | 72.5 | 109 | 79025 | https://www.nutritionvalue.org/Game\_meat%2C\_raw%2C\_goat\_nutritional\_value.html?size=100+g | 700 | 6774 | 56 | 2 | NA |
| Sheep/Goat | Ovis/capra | Bovidae | Sheep/goats | Mammal | Ungulates | Mixed Land | domestic | medium | medium | Annasminde IV (MLF01354) | Annasminde IV | settlement | coastal | Neolithic-Bronze age | NeoBA | 45.0 | 100.0 | 72.5 | 109 | 79025 | https://www.nutritionvalue.org/Game\_meat%2C\_raw%2C\_goat\_nutritional\_value.html?size=100+g | 700 | 6774 | 67 | 2 | NA |
| Sheep/Goat | Ovis/capra | Bovidae | Sheep/goats | Mammal | Ungulates | Mixed Land | domestic | medium | medium | Annasminde V (MLF01355) | Annasminde V | settlement | coastal | Bronze age | BA | 45.0 | 100.0 | 72.5 | 109 | 79025 | https://www.nutritionvalue.org/Game\_meat%2C\_raw%2C\_goat\_nutritional\_value.html?size=100+g | 700 | 6774 | 43 | 1 | NA |
| Sheep/Goat | Ovis/capra | Bovidae | Sheep/goats | Mammal | Ungulates | Mixed Land | domestic | medium | medium | Finlandsvej II (MLF02548) | Finlandsvej II | settlement | coastal | Neolithic-Iron Age | NeoBA | 45.0 | 100.0 | 72.5 | 109 | 79025 | https://www.nutritionvalue.org/Game\_meat%2C\_raw%2C\_goat\_nutritional\_value.html?size=100+g | 700 | 6774 | 26 | 1 | NA |
| Sheep/Goat | Ovis/capra | Bovidae | Sheep/goats | Mammal | Ungulates | Mixed Land | domestic | medium | medium | Gokartbane (MLF01333) | Gokartbane | settlement | coastal | Middle Neolithic | Neo | 45.0 | 100.0 | 72.5 | 109 | 79025 | https://www.nutritionvalue.org/Game\_meat%2C\_raw%2C\_goat\_nutritional\_value.html?size=100+g | 700 | 6774 | 11 | 1 | NA |
| Sheep/Goat | Ovis/capra | Bovidae | Sheep/goats | Mammal | Ungulates | Mixed Land | domestic | medium | medium | RGS90 (MLF00952) | RGS90 | settlement | coastal | Neolithic-Bronze age | BA | 45.0 | 100.0 | 72.5 | 109 | 79025 | https://www.nutritionvalue.org/Game\_meat%2C\_raw%2C\_goat\_nutritional\_value.html?size=100+g | 700 | 6774 | 1 | 1 | Bangsgaard 2016 |
| Sheep/Goat | Ovis/capra | Bovidae | Sheep/goats | Mammal | Ungulates | Mixed Land | domestic | medium | medium | Strandholm I (MLF00909-I+II) | Strandholm I | settlement | coastal | Neolithic | Neo | 45.0 | 100.0 | 72.5 | 109 | 79025 | https://www.nutritionvalue.org/Game\_meat%2C\_raw%2C\_goat\_nutritional\_value.html?size=100+g | 700 | 6774 | 23 | 3 | NA |
| Sheep/Goat | Ovis/capra | Bovidae | Sheep/goats | Mammal | Ungulates | Mixed Land | domestic | medium | medium | Strandholm V (MLF01182) | Strandholm V | settlement | coastal | Neolithic-Bronze age | Neo | 45.0 | 100.0 | 72.5 | 109 | 79025 | https://www.nutritionvalue.org/Game\_meat%2C\_raw%2C\_goat\_nutritional\_value.html?size=100+g | 700 | 6774 | 1 | 1 | NA |
| Sheep/Goat | Ovis/capra | Bovidae | Domestic sheep | Mammal | Ungulates | Mixed Land | domestic | medium | medium | Strandholm VI (MLF01232-I) | Strandholm VI | settlement | coastal | Bronze age | BA | 45.0 | 100.0 | 72.5 | 109 | 79025 | https://www.nutritionvalue.org/Game\_meat%2C\_raw%2C\_goat\_nutritional\_value.html?size=100+g | 700 | 6774 | 11 | 3 | NA |
| Sheep/Goat | Ovis/capra | Bovidae | Sheep/goats | Mammal | Ungulates | Mixed Land | domestic | medium | medium | Syltholm II (MLF00906-I+II+III) | Syltholm II | settlement | coastal | Mesolithic-Neolithic | MesoNeo | 45.0 | 100.0 | 72.5 | 109 | 79025 | https://www.nutritionvalue.org/Game\_meat%2C\_raw%2C\_goat\_nutritional\_value.html?size=100+g | 700 | 6774 | 41 | 3 | Analysis + MNI: Pernille Bangsgaard |
| Sheep/Goat | Ovis/capra | Bovidae | Sheep/goats | Mammal | Ungulates | Mixed Land | domestic | medium | medium | Syltholm IX (MLF00935-I+II+III) | Syltholm IX | settlement | coastal | Early Neolithic-Early Bronze Age | Neo | 45.0 | 100.0 | 72.5 | 109 | 79025 | https://www.nutritionvalue.org/Game\_meat%2C\_raw%2C\_goat\_nutritional\_value.html?size=100+g | 700 | 6774 | 26 | 4 | NA |
| Sheep/Goat | Ovis/capra | Bovidae | Sheep/goats | Mammal | Ungulates | Mixed Land | domestic | medium | medium | Syltholm X (MLF00936) | Syltholm X | settlement | coastal | Late Mesolithic-early Neolithic | MesoNeo | 45.0 | 100.0 | 72.5 | 109 | 79025 | https://www.nutritionvalue.org/Game\_meat%2C\_raw%2C\_goat\_nutritional\_value.html?size=100+g | 700 | 6774 | 3 | 1 | NA |
| Sheep/Goat | Ovis/capra | Bovidae | Sheep/goats | Mammal | Ungulates | Mixed Land | domestic | medium | medium | Syltholm XIII (MLF00939-I) | Syltholm XIII | settlement | coastal | Late Mesolithic-early Neolithic | MesoNeo | 45.0 | 100.0 | 72.5 | 109 | 79025 | https://www.nutritionvalue.org/Game\_meat%2C\_raw%2C\_goat\_nutritional\_value.html?size=100+g | 700 | 6774 | 3 | 1 | NA |
| Shorthorn Sculpin | Myoxocephalus scorpius | Cottidae | Shortern sculpins | Fish | Saltwater fish | Marine | wild | small | small | Annasminde IV (MLF01354) | Annasminde IV | settlement | coastal | Neolithic-Bronze age | NeoBA | 0.1 | 0.5 | 0.3 | 97 | 291 | http://traditionalanimalfoods.org/fish/saltwater/page.aspx?id=6438 (Kuhnlein & Humphries 2017) | 15 | 1164 | 2 | indet | NA |
| Shorthorn Sculpin | Myoxocephalus scorpius | Cottidae | Shortern sculpins | Fish | Saltwater fish | Marine | wild | small | small | Strandholm VI (MLF01232-I) | Strandholm VI | settlement | coastal | Bronze age | BA | 0.1 | 0.5 | 0.3 | 97 | 291 | http://traditionalanimalfoods.org/fish/saltwater/page.aspx?id=6438 (Kuhnlein & Humphries 2017) | 15 | 1164 | 1 | 1 | NA |
| Shorthorn Sculpin | Myoxocephalus scorpius | Cottidae | Shortern sculpins | Fish | Saltwater fish | Marine | wild | small | small | Syltholm II (MLF00906-II+III) | Syltholm II | settlement | coastal | Mesolithic-Neolithic | MesoNeo | 0.1 | 0.5 | 0.3 | 97 | 291 | http://traditionalanimalfoods.org/fish/saltwater/page.aspx?id=6438 (Kuhnlein & Humphries 2017) | 15 | 1164 | 67 | indet | NA |
| Shorthorn Sculpin | Myoxocephalus scorpius | Cottidae | Shortern sculpins | Fish | Saltwater fish | Marine | wild | small | small | Syltholm VII (MLF00933-II) | Syltholm VII | settlement | coastal | Late Palaeolithic-middle Neolithic | Neo | 0.1 | 0.5 | 0.3 | 97 | 291 | http://traditionalanimalfoods.org/fish/saltwater/page.aspx?id=6438 (Kuhnlein & Humphries 2017) | 15 | 1164 | 11 | indet | NA |
| Shorthorn Sculpin | Myoxocephalus scorpius | Cottidae | Shortern sculpins | Fish | Saltwater fish | Marine | wild | small | small | Syltholm X (MLF00936) | Syltholm X | settlement | coastal | Late Mesolithic-early Neolithic | MesoNeo | 0.1 | 0.5 | 0.3 | 97 | 291 | http://traditionalanimalfoods.org/fish/saltwater/page.aspx?id=6438 (Kuhnlein & Humphries 2017) | 15 | 1164 | 1 | 1 | NA |
| Shorthorn Sculpin | Myoxocephalus scorpius | Cottidae | Shortern sculpins | Fish | Saltwater fish | Marine | wild | small | small | Syltholm XIII (MLF00939-I) | Syltholm XIII | settlement | coastal | Late Mesolithic-early Neolithic | MesoNeo | 0.1 | 0.5 | 0.3 | 97 | 291 | http://traditionalanimalfoods.org/fish/saltwater/page.aspx?id=6438 (Kuhnlein & Humphries 2017) | 15 | 1164 | 1 | 1 | NA |
| Snake | Serpentes | Serpentes | Snakes | Reptile | Reptiles | Land | wild | small | small | Syltholm II (MLF00906-II) | Syltholm II | settlement | coastal | Mesolithic-Neolithic | MesoNeo | 0.1 | 2.0 | 1.0 | 108 | 1112 | https://www.nutritionvalue.org/Game\_meat%2C\_raw%2C\_deer\_nutritional\_value.html?size=100%20g | 20 | 3337 | 1 | 1 | NA |
| Spiny Dogfish | Squalus acanthias | Squalidae | Spiny dogfish | Fish | Fish | Marine | wild | medium | small | Syltholm IX (MLF00935-I) | Syltholm IX | settlement | coastal | Early Neolithic-Early Bronze Age | Neo | 5.0 | 10.0 | 7.5 | 167 | 12525 | http://traditionalanimalfoods.org/fish.aspx (Kuhnlein & Humphries 2017) | 50 | 15030 | 1 | 1 | NA |
| Spiny Dogfish | Squalus acanthias | Squalidae | Spiny dogfish | Fish | Fish | Marine | wild | medium | small | Syltholm XIII (MLF00939-I) | Syltholm XIII | settlement | coastal | Late Mesolithic-early Neolithic | MesoNeo | 5.0 | 10.0 | 7.5 | 167 | 12525 | http://traditionalanimalfoods.org/fish.aspx (Kuhnlein & Humphries 2017) | 50 | 15030 | 2 | 1 | NA |
| Swan | Cygnus sp. | Anatidae | Swans | Bird | Waterfowl | Wetland | wild | large | small | Strandholm I (MLF00909-II) | Strandholm I | settlement | coastal | Neolithic | Neo | 7.0 | 15.0 | 11.0 | 130 | 14300 | Morin et al 2022 | 250 | 3432 | 1 | 1 | NA |
| Swan | Cygnus sp. | Anatidae | Swans | Bird | Waterfowl | Wetland | wild | large | small | Syltholm II (MLF00906-I+II+III) | Syltholm II | settlement | coastal | Mesolithic-Neolithic | MesoNeo | 7.0 | 15.0 | 11.0 | 130 | 14300 | Morin et al 2022 | 250 | 3432 | 25 | 5 | Analysis: Pernille Bangsgaard |
| Swan | Cygnus sp. | Anatidae | Swans | Bird | Waterfowl | Wetland | wild | large | small | Syltholm IX (MLF00935-I+II) | Syltholm IX | settlement | coastal | Early Neolithic-Early Bronze Age | Neo | 7.0 | 15.0 | 11.0 | 130 | 14300 | Morin et al 2022 | 250 | 3432 | 3 | 2 | NA |
| Swan | Cygnus sp. | Anatidae | Swans | Bird | Waterfowl | Wetland | wild | large | small | Syltholm X (MLF00936) | Syltholm X | settlement | coastal | Late Mesolithic-early Neolithic | MesoNeo | 7.0 | 15.0 | 11.0 | 130 | 14300 | Morin et al 2022 | 250 | 3432 | 3 | 1 | NA |
| Swan | Cygnus sp. | Anatidae | Swans | Bird | Waterfowl | Wetland | wild | large | small | Syltholm XIII (MLF00939-I) | Syltholm XIII | settlement | coastal | Late Mesolithic-early Neolithic | MesoNeo | 7.0 | 15.0 | 11.0 | 130 | 14300 | Morin et al 2022 | 250 | 3432 | 5 | 1 | NA |
| Tern | Sterninae | Laridae | Terns | Bird | Waders/Shorebirds | Coastal | wild | small | small | Syltholm X (MLF00936) | Syltholm X | settlement | coastal | Late Mesolithic-early Neolithic | MesoNeo | 0.1 | 0.2 | 0.1 | 132 | 172 | Morin et al 2022 | 15 | 686 | 1 | 1 | NA |
| Three-Spined Stickleback | Gasterosteus aculeatus | Gasteristeidae | Three-spined sticklebacks | Fish | Freshwater fish | Mixed water | wild | small | small | Strandholm VI (MLF01232-I) | Strandholm VI | settlement | coastal | Bronze age | BA | 0.0 | 0.0 | 0.0 | 50 | 10 | Inferred value | 10 | 60 | 1 | 1 | NA |
| Three-Spined Stickleback | Gasterosteus aculeatus | Gasteristeidae | Three-spined sticklebacks | Fish | Freshwater fish | Mixed water | wild | small | small | Syltholm II (MLF00906-II+III) | Syltholm II | settlement | coastal | Mesolithic-Neolithic | MesoNeo | 0.0 | 0.0 | 0.0 | 50 | 10 | Inferred value | 10 | 60 | 21 | indet | NA |
| Three-Spined Stickleback | Gasterosteus aculeatus | Gasteristeidae | Three-spined sticklebacks | Fish | Freshwater fish | Mixed water | wild | small | small | Syltholm VII (MLF00933-II+III) | Syltholm VII | settlement | coastal | Late Palaeolithic-middle Neolithic | Neo | 0.0 | 0.0 | 0.0 | 50 | 10 | Inferred value | 10 | 60 | 6 | 1 | NA |
| Toad | Bufo sp. | Anura | Frogs/toads | Amphibians | Amphibian | Wetland | wild | small | small | Syltholm II (MLF00906-II) | Syltholm II | settlement | coastal | Mesolithic-Neolithic | MesoNeo | 0.0 | 0.1 | 0.1 | 90 | 45 | Morin et al 2022 | 15 | 180 | 1 | 1 | NA |
| True Frog | Ranidae | Anura | Frogs/toads | Amphibians | Amphibian | Wetland | wild | small | small | Syltholm II (MLF00906-II) | Syltholm II | settlement | coastal | Mesolithic-Neolithic | MesoNeo | 0.0 | 0.1 | 0.0 | 69 | 21 | Morin et al 2022 | 15 | 83 | 1 | 1 | NA |
| True Thrush | Turdus sp | Turdidae | Thrushes | Bird | Landbirds | Forest | wild | small | small | Syltholm II (MLF00906-I+II) | Syltholm II | settlement | coastal | Mesolithic-Neolithic | MesoNeo | 0.1 | 0.1 | 0.1 | 94 | 94 | NA | 15 | 376 | 7 | 2 | Analysis: Pernille Bangsgaard |
| Tuna | Thunnus thynnus | Scombridae | Tunas | Fish | Fish | Marine | wild | large | medium | Syltholm II (MLF00906-III) | Syltholm II | settlement | coastal | Mesolithic-Neolithic | MesoNeo | 10.0 | 250.0 | 130.0 | 144 | 187200 | http://traditionalanimalfoods.org/fish/saltwater/page.aspx?id=6442 (Kuhnlein & Humphries 2017) | 1200 | 9360 | 1 | indet | NA |
| Turbot | Psetta maxima | Scophthalmidae | Turbots | Fish | Fish | Marine | wild | large | small | Syltholm II (MLF00906-II) | Syltholm II | settlement | coastal | Mesolithic-Neolithic | MesoNeo | 0.5 | 12.0 | 6.3 | 95 | 5938 | https://www.nutritionvalue.org/Fish%2C\_raw%2C\_european%2C\_turbot\_nutritional\_value.html?size=100+g | 40 | 8906 | 3 | indet | NA |
| Turbot/Brill | Psetta maxima/S. rhombus | Scophthalmidae | Turbots | Fish | Fish | Marine | wild | large | small | Syltholm II (MLF00906-II) | Syltholm II | settlement | coastal | Mesolithic-Neolithic | MesoNeo | 0.5 | 12.0 | 6.3 | 95 | 5938 | https://www.nutritionvalue.org/Fish%2C\_raw%2C\_european%2C\_turbot\_nutritional\_value.html?size=100+g | 40 | 8906 | 7 | indet | NA |
| Wader/Gull/Auk | Charadriiformes | Alcidae | Gulls | Bird | Waders/Shorebirds | Coastal | wild | large | small | Syltholm IX (MLF00935-III) | Syltholm IX | settlement | coastal | Early Neolithic-Early Bronze Age | Neo | 0.5 | 5.0 | 2.8 | 127 | 3493 | Morin et al 2022 | 100 | 2096 | 1 | 1 | NA |
| Wading Bird | Calidris sp. | Alcidae | Waders | Bird | Waders/Shorebirds | Wetland | wild | small | small | Syltholm IX (MLF00935-II) | Syltholm IX | settlement | coastal | Early Neolithic-Early Bronze Age | Neo | 0.0 | 1.2 | 0.6 | 94 | 573 | NA | 30 | 1147 | 1 | 1 | NA |
| Wagtail/Longclaw/Pipit | Motacillidae | Motacillidae | Wagtails/longclaws/pipits | Bird | Landbirds | Land | wild | small | small | Syltholm XIII (MLF00939-I) | Syltholm XIII | settlement | coastal | Late Mesolithic-early Neolithic | MesoNeo | 0.0 | 0.1 | 0.1 | 94 | 47 | NA | 15 | 188 | 1 | 1 | NA |
| Warbler/Babbler | Sylviidae | Sylviidae | Warblers/babblers | Bird | Landbirds | Forest | wild | small | small | Syltholm XIII (MLF00939-I) | Syltholm XIII | settlement | coastal | Late Mesolithic-early Neolithic | MesoNeo | 0.0 | 0.0 | 0.0 | 94 | 28 | NA | 15 | 113 | 1 | 1 | NA |
| Wild Boar | Sus scrofa | Suidae | Wild boars | Mammal | Ungulates | Mixed Land | wild | medium | medium | RGS90 (MLF00952) | RGS90 | settlement | coastal | Neolithic-Bronze age | BA | 50.0 | 200.0 | 125.0 | 122 | 152500 | https://www.nutritionvalue.org/Game\_meat%2C\_raw%2C\_wild%2C\_boar\_nutritional\_value.html?size=100+g | 900 | 10167 | 1 | 1 | Bangsgaard 2016 |
| Wild Boar | Sus scrofa | Suidae | Wild boars | Mammal | Ungulates | Mixed Land | wild | medium | medium | Syltholm II (MLF00906-I+II+III) | Syltholm II | settlement | coastal | Mesolithic-Neolithic | MesoNeo | 50.0 | 200.0 | 125.0 | 122 | 152500 | https://www.nutritionvalue.org/Game\_meat%2C\_raw%2C\_wild%2C\_boar\_nutritional\_value.html?size=100+g | 900 | 10167 | 17 | 5 | Analysis: Pernille Bangsgaard |
| Wild Boar | Sus scrofa | Suidae | Wild boars | Mammal | Ungulates | Mixed Land | wild | medium | medium | Syltholm IX (MLF00935-I) | Syltholm IX | settlement | coastal | Early Neolithic-Early Bronze Age | Neo | 50.0 | 200.0 | 125.0 | 122 | 152500 | https://www.nutritionvalue.org/Game\_meat%2C\_raw%2C\_wild%2C\_boar\_nutritional\_value.html?size=100+g | 900 | 10167 | 1 | 1 | NA |
| Wild Boar | Sus scrofa | Suidae | Wild boars | Mammal | Ungulates | Mixed Land | wild | medium | medium | Syltholm X (MLF00936) | Syltholm X | settlement | coastal | Late Mesolithic-early Neolithic | MesoNeo | 50.0 | 200.0 | 125.0 | 122 | 152500 | https://www.nutritionvalue.org/Game\_meat%2C\_raw%2C\_wild%2C\_boar\_nutritional\_value.html?size=100+g | 900 | 10167 | 1 | 1 | NA |
| Wild Boar | Sus scrofa | Suidae | Wild boars | Mammal | Ungulates | Mixed Land | wild | medium | medium | Syltholm XIII (MLF00939-II) | Syltholm XIII | settlement | coastal | Late Mesolithic-early Neolithic | MesoNeo | 50.0 | 200.0 | 125.0 | 122 | 152500 | https://www.nutritionvalue.org/Game\_meat%2C\_raw%2C\_wild%2C\_boar\_nutritional\_value.html?size=100+g | 900 | 10167 | 2 | 1 | NA |

---

## Annasminde (II, III, IV, V)

Here are the four Annasminde sites, which all date to the Neolithic
and Bronze Age. Annasminde II code chunks show how the models are
created.

```
Annasminde_II <- data[data$Site_name %in% "Annasminde II", ]

Annasminde_II$PNISP <- (Annasminde_II$NISP / sum(Annasminde_II$NISP)) * 100

Annasminde_II$PERR_rank <- rank(-Annasminde_II$PERR, ties.method = "min")
Annasminde_II$NISP_rank <- rank(-Annasminde_II$NISP, ties.method = "min") 

total_nisp <- sum(Annasminde_II$NISP)

Annasminde_IIa <- ggplot(Annasminde_II, aes(PERR, PNISP)) + 
  scale_x_continuous(breaks = seq(0, 18000, 3000), limits = c(0, 18001)) + 
  scale_y_continuous(breaks = seq(0, 100, 10), expand = c(0,0), limits = c(0, 101)) + 
  geom_label_repel(aes(label = Common_name, fill = Biome), size = 4, max.overlaps = 15, nudge_x = 0.05, nudge_y = 0.05) +
  #geom_smooth(method='lm', formula = my.formula, se=F, color= "black", size = 0.5) + 
  stat_poly_eq(formula = my.formula, label.x = "left", label.y = "top", aes(label = paste(after_stat(p.value.label),  sep = "~~~")), parse = TRUE, size = 3, color = "black", small.r = TRUE, small.p = TRUE) +
  theme_bw() + labs(x = 'PERR (Post encounter return rate)', y = 'NISP %') + theme(axis.ticks = element_line()) + 
  theme(axis.title.x = element_text(size = 10), axis.title.y = element_text(size = 10)) +
  labs(title = "Annasminde II", subtitle = glue("Neolithic to Bronze Age | Total NISP = ", total_nisp)) + theme(legend.title = element_blank()) + theme(legend.position="bottom") +
  theme(plot.title = element_text(hjust = 0, size = 15, face = "bold"), plot.subtitle = element_text(hjust = 0, size = 10, face = "italic")) +
  theme(panel.grid.major = element_blank(), panel.grid.minor = element_blank(), panel.background = element_blank(), axis.line = element_line(colour = "black"), 
  panel.border = element_rect(colour = "black", fill = NA, size = 0.5)) +
  scale_fill_manual(values = mlf_col_biome) + guides(fill = guide_legend(override.aes = list(label = "")))

Annasminde_IIb <- ggplot(Annasminde_II, aes(PERR_rank, NISP_rank)) +
  scale_x_continuous(breaks = seq(0, 10, 2), limits = c(0, 11)) + 
  scale_y_continuous(breaks = seq(0, 10, 2), expand = c(0,0), limits = c(0, 11)) + 
  geom_label_repel(aes(label = Common_name, fill = Biome), size = 4, max.overlaps = 15, nudge_x = 0.05, nudge_y = 0.05) +
  #geom_smooth(method='lm', formula = my.formula, se=F, color= "black", size = 0.5) + 
  stat_poly_eq(formula = my.formula, label.x = "left", label.y = "top", aes(label = paste(after_stat(p.value.label),  sep = "~~~")), parse = TRUE, size = 3, color = "black", small.r = TRUE, small.p = TRUE) +
  labs(x = 'PERR ranks', y = 'NISP ranks') + theme(axis.title.x = element_text(size = 10), axis.title.y = element_text(size = 10)) +
  labs(title = "Annasminde II", subtitle = glue("Neolithic to Bronze Age | Total NISP = ", total_nisp)) + theme(legend.title = element_blank()) + theme(legend.position="bottom") +
  theme(plot.title = element_text(hjust = 0, size = 15, face = "bold"), plot.subtitle = element_text(hjust = 0, size = 10, face = "italic")) +
  theme(panel.grid.major = element_blank(), panel.grid.minor = element_blank(), panel.background = element_blank(), axis.line = element_line(colour = "black"), 
  panel.border = element_rect(colour = "black", fill = NA, size = 0.5)) + 
  scale_fill_manual(values = mlf_col_biome) + guides(fill = guide_legend(override.aes = list(label = "")))
```

```
Annasminde_IIplot <- ggarrange(Annasminde_IIa, Annasminde_IIb, nrow=2, ncol=1, common.legend = T, legend = "bottom")
Annasminde_IIplot
```

```
Annasminde_IIIplot <- ggarrange(Annasminde_IIIa, Annasminde_IIIb, nrow=2, ncol=1, common.legend = T, legend = "bottom")
Annasminde_IIIplot
```

```
Annasminde_IVplot <- ggarrange(Annasminde_IVa, Annasminde_IVb, nrow=2, ncol=1, common.legend = T, legend = "bottom")
Annasminde_IVplot
```

```
Annasminde_Vplot <- ggarrange(Annasminde_Va, Annasminde_Vb, nrow=2, ncol=1, common.legend = T, legend = "bottom")
Annasminde_Vplot
```

---

## Syltholm II

The Syltholm II faunal assemblage is very large, resulting in the
model outputs looking slightly chaotic, and does not provide a good
overview of prey choice (see figures below)

### Syltholm II alternative version

To avoid the many overlaps of the data points in the models above, we
decided to subset the data points according to each species generally
preferred habitats (named biome in dataset).

Under here you will see the code chunk for the first group called
*marine*, which includes marine, mixed water and mixed saltwater
categories. The other groups are *coastal* (coastal, freshwater
and wetland) and *land* (land, forest, mixed land)

```
marine_group <- Syltholm_II %>%
  filter(Biome %in% c("Marine", "Mixed water", "Mixed saltwater"))

marine_SIIa <- ggplot(marine_group, aes(PERR, PNISP)) + 
  scale_x_continuous(breaks = seq(0, 20000,4000), limits = c(0, 20001)) + 
  scale_y_continuous(breaks = seq(0, 100, 10), expand = c(0,0), limits = c(0, 101)) + 
  geom_label_repel(aes(label = Common_name, fill = Biome), size = 2.5, max.overlaps = 50, nudge_x = 0.05, nudge_y = 0.05) + 
  #geom_smooth(method='lm', formula = my.formula, se=F, color= "black", size = 0.5) + 
  stat_poly_eq(formula = my.formula, label.x = "left", label.y = "top", aes(label = paste(after_stat(p.value.label),  sep = "~~~")), parse = TRUE, size = 3, color = "black", small.r = TRUE, small.p = TRUE) +
  theme_bw() + labs(x = 'PERR (Post encounter return rate)', y = 'NISP %') + theme(axis.ticks = element_line()) +
  theme(axis.title.x = element_text(size = 10), axis.title.y = element_text(size = 10)) +
  theme(legend.title = element_blank()) + theme(legend.position="bottom") +
  theme(panel.grid.major = element_blank(), panel.grid.minor = element_blank(), panel.background = element_blank(), 
  axis.line = element_line(colour = "black"), panel.border = element_rect(colour = "black", fill = NA, size = 0.5)) +
  scale_fill_manual(values = mlf_col_biome) + guides(fill = guide_legend(override.aes = list(label = "")))

marine_SIIb <- ggplot(marine_group, aes(PERR_rank, NISP_rank)) +
  scale_x_continuous(breaks = seq(0, 60, 5), limits = c(0, 61)) + 
  scale_y_continuous(breaks = seq(0, 60, 5), expand = c(0,0), limits = c(0, 61)) + 
  geom_label_repel(aes(label = Common_name, fill = Biome), size = 2.5, max.overlaps = 25, nudge_x = 0.05, nudge_y = 0.05) +
  #geom_smooth(method='lm', formula = my.formula, se=F, color= "black", size = 0.5) + 
  stat_poly_eq(formula = my.formula, label.x = "left", label.y = "top", aes(label = paste(after_stat(p.value.label),  sep = "~~~")), parse = TRUE, size = 3, color = "black", small.r = TRUE, small.p = TRUE) +
  labs(x = 'PERR ranks', y = 'NISP ranks') +
  theme(axis.title.x = element_text(size = 10), axis.title.y = element_text(size = 10)) +
  theme(legend.title = element_blank()) + theme(legend.position="bottom") +
  theme(panel.grid.major = element_blank(), panel.grid.minor = element_blank(), panel.background = element_blank(), 
  axis.line = element_line(colour = "black"), panel.border = element_rect(colour = "black", fill = NA, size = 0.5)) +
  scale_fill_manual(values = mlf_col_biome) + guides(fill = guide_legend(override.aes = list(label = "")))
```

Here you can see the figures the codes produced.

```
SII_biomes_a <- ggarrange(marine_SIIa, marine_SIIb, nrow=2, ncol=1, common.legend = T, legend = "bottom")
SII_biomes_a
```

```
SII_biomes_b <- ggarrange(coastal_SIIa, coastal_SIIb, nrow=2, ncol=1, common.legend = T, legend = "bottom") 
SII_biomes_b
```

```
SII_biomes_c <- ggarrange(land_SIIa, land_SIIb, nrow=2, ncol=1, common.legend = T, legend = "bottom") 
SII_biomes_c
```

---

## Syltholm V

We copied the code structure introduced with the Annasminde II
assemblage for the remaining sites:

```
SyltholmV <- ggarrange(Syltholm_Va, Syltholm_Vb, nrow=2, ncol=1, common.legend = T, legend = "bottom")

SyltholmV
```

---

## Syltholm VII

```
SyltholmVII <- ggarrange(Syltholm_VIIa, Syltholm_VIIb, nrow=2, ncol=1, common.legend = T, legend = "bottom")

SyltholmVII
```

---

## Syltholm IX

```
SyltholmIX <- ggarrange(Syltholm_IXa, Syltholm_IXb, nrow=2, ncol=1, common.legend = T, legend = "bottom")

SyltholmIX
```

---

## Syltholm X

```
SyltholmX <- ggarrange(Syltholm_Xa, Syltholm_Xb, nrow=2, ncol=1, common.legend = T, legend = "bottom")

SyltholmX
```

---

## Syltholm XIII

```
SyltholmXIII <- ggarrange(Syltholm_XIIIa, Syltholm_XIIIb, nrow=2, ncol=1, common.legend = T, legend = "bottom")

SyltholmXIII
```

---

## Syltholm XIV

```
SyltholmXIV <- ggarrange(Syltholm_XIVa, Syltholm_XIVb, nrow=2, ncol=1, common.legend = T, legend = "bottom")

SyltholmXIV
```

---

## Finlandsvej II

```
FinlandsvejII <- ggarrange(Finlandsvej_IIa, Finlandsvej_IIb, nrow=2, ncol=1, common.legend = T, legend = "bottom")

FinlandsvejII
```

---

## Gokartbane

```
Gokartbaneplot <- ggarrange(Gokartbanea, Gokartbaneb, nrow=2, ncol=1, common.legend = T, legend = "bottom")

Gokartbaneplot
```

---

## RGS90

```
RGS90plot <- ggarrange(RGS90a, RGS90b, nrow=2, ncol=1, common.legend = T, legend = "bottom")

RGS90plot
```

---

## Strandholm (I, V & VI)

```
StrandholmI <- ggarrange(Strandholm_Ia, Strandholm_Ib, nrow=2, ncol=1, common.legend = T, legend = "bottom")
StrandholmI
```

```
StrandholmV <- ggarrange(Strandholm_Va, Strandholm_Vb, nrow=2, ncol=1, common.legend = T, legend = "bottom")
StrandholmV
```

```
StrandholmVI <- ggarrange(Strandholm_VIa, Strandholm_VIb, nrow=2, ncol=1, common.legend = T, legend = "bottom")
StrandholmVI
```

## The End

Thank you for scrolling all the way down to the bottom of this
file!

Here are some bottom-feeders as a token of your dedication!

**><(((°>** ><(((((°>
**><(((°>** ><(((((°>
**><(((°>** ><(((((°>
**><(((°>** ><(((((°>
**><(((°>**
