# Supplementary material for: Fishing for millennia: Effects and impacts of prehistoric fishing in the Syltholm Fjord, Denmark
Source: PLoS One. 2026 May 13;21(5):e0347863. doi: 10.1371/journal.pone.0347863 (PMC13170857; doi:10.1371/journal.pone.0347863)
Supplement: S3 File — (DOCX) [file pone.0347863.s006.docx]

Bangsgaard, P., 2016. Lokalitet Strandholm MLF00652-A. Titel: Et knoglemateriale fra Strandholm, dateret primært til Neolitikum., ArchaeoScience. Statens Naturhistoriske Museum, Københaven.

Bennike, O., Philippsen, B., Groß, D., Jessen, C., 2022. Holocene shore-level changes, southern Lolland and Femern Belt, Denmark. Journal of Quaternary Science 38, 440–451. <https://doi.org/10.1002/jqs.3479>

Chaudesaigues-Clausen, S., 2023. Mesolithic persistence and Neolithic emergence at Syltholm II (MLF00906-III). Osseous artefacts before and after 4000 BCE on the coast of Lolland, Denmark, in: Groß, D., Rothstein, M. (Eds.), Changing Identity in a Changing World. Current Studies on the Stone Age in Northern Europe around 4000 Cal BC. Sidestone, Leiden, pp. 149–164.

Dekker, J., 2025. Burnt to a crisp. Disentangling the taxonomic composition of foodcrusts via palaeoproteomics (Dissertation). University of Copenhagen, Copenhagen.

Glykou, A., Lõugas, L., Piličiauskienė, G., Schmölcke, U., Eriksson, G., Lidén, K., 2021. Reconstructing the ecological history of the extinct harp seal population of the Baltic Sea. Quaternary Science Reviews 251, 106701. <https://doi.org/10.1016/j.quascirev.2020.106701>

Jensen, L.E., Jensen, S., Knöchel Christensen, A.V., Kring Mortensen, N.M., Deichmann, P.C.M., Måge, B., Mathiesen, A.-L.M., Stafseth, T., 2016. Syltholmudgravningerne - jagten på stenalderens jægere, fiskere og bønder i et druknet landskab. Aarbøger for Nordisk Oldkyndighed og Historie 2015, 33–62.

Philippsen, B., 2018. Reservoir Effects in a Stone Age Fjord on Lolland, Denmark. Radiocarbon 60, 653–665. <https://doi.org/10.1017/rdc.2018.6>

Koivisto, S., Robson, H.K., Philippsen, B., Stafseth, T., Brinch, M., Schmölcke, U., Astrup, P.M., Casati, C., Henriksen, M.B., Uldum, O., Lundbye, M., Maring, R., Kanstrup, M., Måge, B.T., Groß, D., 2024. Fishing with stationary wooden structures in Stone Age Denmark: new evidence from Syltholm Fjord, southern Lolland. Proceedings of the Prehistoric Society 90, 147–176. <https://doi.org/doi.org/10.1017/ppr.2024.15>

Måge, B., 2019. Syltholm VII: En atypisk beliggende Ahrensburglokalitet fra Syd-Lolland. Gefjon: arkæologi og nyere tid 4, 176–193.

Måge, B.T., Groß, D., Kanstrup, M., 2023. The Femern project: a large-scale excavation of a Stone Age landscape, in: Groß, D., Rothstein, M. (Eds.), Changing Identity in a Changing World. Current Studies on the Stone Age in Northern Europe around 4000 Cal BC. Sidestone, Leiden, pp. 21–32.

Sørensen, S.A., 2020. Ritual depositions in the coastal zone: A case from Syltholm, Denmark, in: Schülke, A. (Ed.), Coastal Landscapes of the Mesolithic. Human Engagement with the Coast from the Atlantic to the Baltic Sea. Routledge, London / New York, pp. 394–414.
